# Supplementary material for: An agent-based model to advance the science of collaborative learning health systems
Source: PLoS One. 2025 Sep 9;20(9):e0332054. doi: 10.1371/journal.pone.0332054 (PMC12419628; doi:10.1371/journal.pone.0332054)
Supplement: S1 Supplement — (PDF) [file pone.0332054.s001.pdf]

Overview, Design concepts, Details (ODD) Protocol for the Collaborative Learning Health System ABM

Seid, Hartley, Bridgeland

1. Purpose and Patterns 3
2. Entities, State Variables, and Scales 3
Patients 3
Clinicians 4
Care Centers 4
Commons 4
Environment 4
3. Process overview and scheduling 4
4. Design concepts 5
Basic Principles 5
Emergence 5
Adaptation 5
Objectives 6
Learning 6
Prediction 6
Sensing 7
Interaction 7
Stochasticity 7
Collectives 9
Observation 9
5. Initialization 10
Patients 11
Clinicians 15
Medical Condition 18
Care Centers 22
The Commons 23
The Enhanced Registry 24
6. Input Data 24
7. Submodels 24
Commons submodel 24
Update Patient submodel 25
Adjust Health Status sub-submodel 25
Update Individual Response Information sub-submodel 26
Patient Commons Contribution sub-submodel 31

Clinical Encounter sub-submodel 32
Phenotype response information 32
Praxis 35
Patient health status 38
Treatment regimen 40
Patient Transition sub-submodel 43
Patient Leave Cohort sub-submodel 47
Update Clinician submodel 48
Clinician Transition sub-submodel 48
Clinician Commmons Contribution sub-submodel 51
Enhanced Registry submodel 51
Patient Arrive submodel 52

1. Purpose and Patterns

The model description follows the ODD (Overview, Design concepts, Details) protocol for describing individual- and agent-based models (Grimm et al. 2006), as updated by Grimm et al. (2020).

The purpose of the CLHS ABM is to establish and assess hypotheses about the general behavior of CLHSs under a range of initial starting conditions and in consideration of key hypothesized mechanisms of action. The model is designed to enable careful thinking about how and why CLHSs work. It is not a tool for prediction: It is meant to help CLHS leaders make better-informed choices about strategies and tactics, and researchers make better-designed experiments. The CLHS ABM models the change in health status over time for a population of patients as a result of patient and clinician interactions under conditions thought to be important to the functioning of a Learning Health Network. The model produces several patterns suggested by the theory of CLHSs. In general, as patient and clinician engagement, influence among patients and clinicians, and ease of knowledge creation and sharing increases, so should the amount of knowledge available for treatment decision making and the health of the patient population increase.

2. Entities, State Variables, and Scales

Patients

Patients are agents representing either an individual with the given condition or the individual and their family. Patients are included as such in order to simplify the model to a dyadic interaction between patient units and clinician units, versus a more complex triadic interaction among patient units, family units, and clinician units. Patients are assigned to a care center and, within a care center, to a clinician.

Patient health status is a dynamic variable representing a patient's current health. It is a ratio variable ranging from 0 (worst) to 1 (best).

Each patient in the simulation has a phenotype, a static nominal variable denoting the degree to which the patient will respond to each of the available treatments.

Each patient has phenotype response information, modeling how much is known about treatment of the medical condition for patients with the same phenotype as the original patient. It is measured on a zero-to-one scale: zero means nothing is known, and one means that as much is known as can be.

Each patient has individual response information, modeling knowledge about their own response to the treatments. It is measured on a zero-to-one scale: zero means nothing is known, and one means that as much is known as can be.

Each patient is engaged with the learning network to some extent. **Engagement** is a dynamic variable, represented on a five-point ordinal scale with the following levels of engagement:

- **Unaware**: the patient is not aware of the existence of the learning network
- **Aware**: the patient is aware of the existence of the learning network, but not participating in it, not actively using the network to improve his care.
- **Participating**: the patient is using the resources in the learning network to improve their own care (e.g., reading a blog post), but not adding resources to it.

- **Contributing**: the patient is both using the learning network to improve their care and is improving the value of the resources in the network (e.g., writing a blog post).
- **Owning**: the patient is contributing new resources to the network (e.g., a tool kit), making it more useful for others

Clinicians

Clinicians are agents representing treating individuals. Clinician states include engagement, an ordinal variable denoted as unaware, aware, contributing, and owning, as with patients. Clinicians are assigned to a care center and have a panel of patients.

Care Centers

Care centers are collective entities (a group of agents) in which patients and clinicians interact. Care centers can be different clinical settings or different health systems. Each care center is typically a focus of quality improvement work or other interventions, and manifests a set of structures and processes for delivering health care. Care center state variables include the number of clinicians and number of patients (ratio variables from 0 to large) assigned to them. Pre-visit planning—a state variable—is the degree to which information about patients and treatments are available in the clinical encounter. Pre-visit planning is an ordinal variable of three levels: low, medium, or high. Enhanced registry is a care center state variable representing a care center's ability to upload, aggregate, and use clinical data collected as part of care. Sometimes referred to as a "triple-use registry" (used for clinical care, quality improvement, and research purposes), the enhanced registry is an important infrastructural element in CLHSs. Enhanced registry has an ordinal scale: low denotes a care center in which data is not uploaded automatically, medium denotes a care center that uploads clinical data automatically to the registry, and high denotes a care center that uploads clinical data and has it available for use at the clinical encounter. The enhanced registry also has a dynamic state variable, the number of enhanced registry records.

Commons

Commons is a singleton entity representing shared knowledge that is available to patients and clinicians. It is included because in CLHSs, patients and clinicians (and others) create and share resources so that more people can get what is needed, when it's needed to make decisions and act in ways that improve health and healthcare. The commons has one state variable, a dynamic variable denoting the number of items in the commons, which is a ratio scale.

Environment

The Environment is a singleton entity that controls the global variables and submodels and keeps track of time. One time step represents one week, long enough for treatment or conditions changes to manifest, but short enough to vary follow up duration. Simulations are run for 104 weeks, long enough to discern changes in the CLHS, but short enough to be meaningful in strategy and planning.

3. Process overview and scheduling

At each time step, the following schedule is executed:

1. The environment updates the time step.
2. The environment executes the **Commons** submodel, changing the number of items in the commons.
3. The patients each execute their **Update Patient** submodel and the clinicians each execute their **Update Clinician** submodel. The patients and clinicians do this in a random order, with a different shuffle of all agents (both patients and clinicians) on each time step.

    a. For each patient, the **Update Patient** submodel updates its health status, its individual response information, its phenotype response information, its contribution to shared knowledge (if any), its engagement, its possible departure from the cohort, and simulates its medical encounter with a clinician via the **Clinical Encounter** sub-submodel.
    b. For each patient, the **Clinical Encounter** sub-submodel determines whether a medical encounter occurs this step between the patient and its clinician. If an encounter does occur, the submodel updates the clinician's perception of the patient's health status, and potentially updates the patient's treatment regimen. The encounter also influences updates in the patient engagement and clinician engagement, although the actual update to the engagement occurs in different submodels.
    c. For each clinician, the **Update Clinician** submodel updates its engagement, and its contribution to shared knowledge (if any).
4. The environment executes the **Enhanced Registry** submodel, occasionally analyzing the records in the enhanced registry and adding items to the commons.
5. The environment executes the **Patient Arrive** submodel, occasionally adding newly created patient agents to the simulation
6. The trace of all patients, clinicians, and the commons are egested.

4. Design concepts

Basic Principles

The CLHS ABM model is based on the theory that CLHSs facilitate collaboration at scale via an actor-oriented architecture. That is, given sufficient actors (in this case, patients and clinicians) with the will and ability to self-organize (in this case, their level of engagement), a commons where actors create and share resources (in this case, the commons and the Enhanced Registry), and processes, protocols, and infrastructure to facilitate multi-actor collaboration (in this case, the clinical encounter, patient and clinician influence, and the degree to which patients and clinicians create, share, and use common resources), the match between needs and treatments should be optimized, leading to better health outcomes.

Emergence

The model's primary results—number of items in the commons, praxis (knowledge available for treatment decision making), and patient outcomes—emerge from patient and clinician engagement, patient and clinician influence, care center states, characteristics of the condition being treated, and the commons.

Adaptation

The following adaptive behaviors are indirect objective-seeking. Patients may leave the cohort if they age out, move away, get sufficiently better, or get sufficiently sicker. Patients and clinicians may become more engaged if they interact with another agent (clinician or patient) with a higher level of engagement. Patients may become less engaged if they interact with a clinician with a lower level of engagement. Clinicians may become less engaged if she suffers burnout. Patients and clinicians can contribute some amount of knowledge at some periodicity to the commons, depending on their level of engagement.

Direct objective-seeking behaviors occur in the context of the clinical encounter. At the first clinical encounter, a treatment is chosen from the full set of available treatments. At subsequent clinical encounters, the patient may change treatments if the patient's perceived health status has not improved since the last clinical encounter (see **Learning and Sensing**). If the patient changes treatments, the alternatives are chosen from the set of available treatments excluding the current treatment. The input driving treatment selection is selection

efficiency, which influences the likelihood that the best (and second best, and third, best, etc.) treatment for that patient's phenotype is chosen from among the alternatives (see **Learning and Prediction**).

Objectives

The objective is improved health status via matching the patient's phenotype to the best treatment for that phenotype. A correct match is one of several influences (along with relapse, condition natural history, condition variability, and treatment effectiveness) that influences patient health status. Calculation of the measure is described in the **Adjust Health Status** sub-submodel in Section 7. The rationale for calculating health status based on selection of the most effective treatment for that phenotype and disease characteristics is based on general, albeit simplified, principles of clinical medicine.

Learning

The adaptive behavior of patients and clinicians—deciding on a treatment—is modeled using an approach that includes learning. This submodel (explained below in the **Treatment Selection** sub-submodel) is based on the theory of reasoned action.[1] In this case, patients and clinicians learn from the current treatment's effect on patient health status (individual response information), as a patient may bring information to the clinical encounter about the effect of the current treatment. They also learn from information about patients with the same phenotype (phenotype response information) based on resources shared in the commons.

The effect of individual response information and phenotype response information are modified by the enhanced registry (see "Enhanced Registry submodel") to yield effective individual response information and effective phenotype response information. Both are measured on a zero-to-one scale. As illustrated in Diagram 1, effective individual response information and effective phenotype response information are combined, during the clinical encounter, to create praxis, a measure of how much is known about the condition of the patient for the purpose of making a treatment decision.

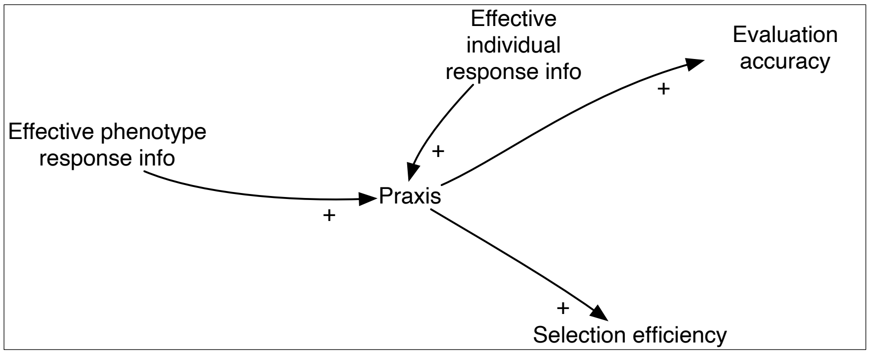

Diagram 1: praxis, selection efficiency, and evaluation accuracy

Prediction

The adaptive behavior of treatment selection is based on an explicit prediction that each treatment has a certain probability of improving the patient's health status. Prediction is modeled to represent how patients and clinicians actually make predictions. That is, if the patient's health status is not perceived as improved, a treatment will be selected from the other available treatments, based on the degree to which patients and clinicians are able to co-create and use knowledge for treatment selection. In the model, this use is referred to as selection efficiency.

Sensing

The true health status of the patient varies from week to week. During a clinical encounter, the clinician and patient evaluate the patient's health status, and whether it has improved since the prior clinical encounter. But the patient's health status is not entirely visible. The patient health status is perceived with some inaccuracy: The patient and clinician may perceive the patient's health status to be better than true health status or they may perceive it to be worse. The sensing of health status is described in more detail in the **Clinical Encounter** sub-submodel.

Interaction

There are three kinds of direct interactions between agents in the model: Interactions within a clinical encounter between patients and clinicians, interactions among patients, and interactions among clinicians. There are also indirect interactions, as when patients or clinicians contribute knowledge to the commons.

Within clinical encounters, as above, patients and clinicians share knowledge for the purpose of decision making. Patients and clinicians can also affect each other's level of engagement. A patient who is unaware in a particular week can become aware as a result of a clinical encounter. If an unaware patient has a clinical encounter with a clinician who is not unaware (i.e. who is aware, participating, contributing, or owning), the patient might become aware of the learning network. A clinical encounter between a patient who is less engaged and a clinician who is more engaged can cause an increase in patient engagement. A clinical encounter between a patient who is more engaged and a clinician who is less engaged can cause a decline in patient engagement.

Interactions among patients and among clinicians can also change engagement levels. Each patient has influence connections to some other patients. The influence network is denser within a care center than across care centers. Over time, an influence link between an unaware patient and a patient who is at least aware can result in the unaware patient becoming aware of the learning network. An influence link can also influence a patient that is already aware of the learning network, to a greater level of engagement, from aware to participating, from participating to contributing, or from contributing to owning. This is modeled in the same way for clinicians.

At each time step, patients and clinicians who are sufficiently engaged might contribute items to the commons. Other patients and their clinicians, if sufficiently engaged, might use those items (or other items in the commons) to increase phenotype response information or individual response information.

Stochasticity

The model uses stochasticity to model initial conditions, to make the agents different from each other. The ten processes that use stochasticity to model initial conditions are the following:

- **Patient initial health status**. Each patient agent starts with an initial health status, a random draw from [0.0, 1.0].
- **Patient phenotype**. Each patient has a phenotype. There are a small number of phenotypes in the model. When a patient agent is initialized, the patient is assigned a phenotype, a random draw from the possible phenotypes.
- **Response of each patient phenotype to each treatment regimen**. The model defines a small set of alternative treatment regimens. The effectiveness of a treatment regimen varies by phenotype: an effective treatment regimen for one phenotype may be ineffective for another. Regimen effectiveness for a phenotype is determined at model initialization via a random draw.

- **Patient care center**. Each patient is assigned to one of the care centers, as one of the initial conditions of the model. The assignment of a patient to a care center is via a random draw among the possible care centers.
- **Clinician care center**. Each clinician agent in the model provides care at one of the care centers, another initial condition of the model. The assignment of a clinician to a care center is via a random draw among the possible care centers.
- **Patient clinician**. Each patient is assigned a clinician agent, one of the clinicians who practices at the care center of the patient, another random draw.
- **Patient engagement**. Each patient begins the simulation with an initial level of engagement, one of the five possible engagement levels: unaware, aware, participating, contributing, or owning. The initial patient engagement is a random draw from this set.
- **Clinician engagement**. Each clinician begins the simulation with an initial level of engagement, one of the same five possible engagement levels. The initial clinician engagement is a random draw from this set.
- **Patient influence links**. There are bidirectional influence links between patients, a subset of the possible pairs of patients. The patient influence links are determined with the initial conditions of the model. Each influence link is a random sample of two elements from the set of patients.
- **Clinician influence links**. There are bidirectional influence links between clinicians, a subset of the possible pairs of clinicians. The clinician influence links are determined with the initial conditions of the model. Each influence link is a random sample of two elements from the set of clinicians.

The model uses stochasticity to simplify the modeling of subprocesses, to avoid modeling all the detail complexity of some subprocesses. The seven subprocesses that use stochasticity for simplification are the following:

- **Other changes in patient health status**. Each patient has a health status that varies from week to week. Some of the change in health status is due to the natural history of the medical condition, and some is due to the effect of the treatment package. But patient health status also varies for other reasons that are beyond the scope of our model. This other variation in health status is modeled as random variation, as a weekly draw on a statistical distribution for each patient.
- **Periodic relapse**. Occasionally a patient may relapse, seeing a sudden worsening of health status. We do not model all the reasons for a relapse, instead treating it as a stochastic process.
- **Perceived patient health status**. During a clinical encounter the clinician-patient team observes the current health status of the patient. This perception differs somewhat from the true (unobserved) health status. We do not model all the reasons for the difference, instead treating the perceived patient health as a draw from a distribution centered on the true health status.
- **Patient departure**. A patient may leave the cohort for reasons unrelated to their health. For example, an adolescent patient's family may move to another geography, beyond the care centers in the cohort. We do not model all the reasons for non-health patient departure, instead treating the departure as a stochastic process.
- **Patient leaving due to improved health status**. If a patient's health becomes sufficiently good, he or she may leave the cohort. The reasons for leaving the cohort are not modeled, except for the patient health. Instead, we simplify by treating patient departure on sufficiently good health as a stochastic process.
- **Patient leaving due to worsening health status**. If a patient's health becomes sufficiently poor, he or she may leave the cohort for more intensive treatment, or the patient may die. The reasons for leaving the cohort are not modeled, except for the patient health. Instead, we simplify by treating patient departure for poor health as a stochastic process.
- **Selection of patient treatment regimen**. The selection of a treatment package for a patient is a decision made by the patient-clinician team. The selection is influenced by how much is known about

the patient's condition, how much is known about the patient's phenotype, and the skill of the clinician. But the selection is modeled as a stochastic draw among the set of treatment regimens, with probabilities for each determined by skill and how much is known.

The model uses stochasticity to reflect the observed choices of actual agents. There are four subprocesses in which stochasticity is used in this way.

- **Change in patient engagement**. Every week a patient may become more engaged or less engaged, changing his or her engagement among the five levels of engagement. The change in patient engagement is influenced by both clinical encounters and by the influence network, the other patients her or she is connected to via bidirectional influence. The model uses stochasticity to reflect the observed pattern of change in patient engagement.
- **Change in clinician engagement**. Every week a clinician may become more engaged or less engaged, changing his or her engagement among the five levels. The change in clinician engagement is influenced by the influence network, the clinicians he or she is connected to via bidirectional influence. The model uses stochasticity to reflect the observed pattern of change in clinician engagement.
- **Patient contribution to the commons**. Every week a sufficiently engaged patient may contribute an item to the commons. The occasional contribution is modeled as a stochastic process, to reflect the observed contributions from patients.
- **Clinician contribution to the commons**. Every week a sufficiently engaged clinician may contribute an item to the commons. The occasional contribution is modeled as a stochastic process, to reflect the observed contributions from clinicians.

Collectives

Care centers are modeled as collectives. They are modeled as explicit entities. Influence networks among patients and among clinicians are denser within care centers than across care centers, so that agents are more likely to influence engagement of others in the same care center compared to agents in a different care center. Care centers state variables—pre-visit planning, enhance registry—also affect how much knowledge is available for use.

Influence networks are modeled as collectives. The influence network structure is specified; the effects of the influence networks are modeled as emerging entirely from agent behaviors, as above, in **Interaction**.

Observation

Key outputs from the model include median patient health status, praxis, and number of items in the commons. For each, values and change from previous value are plotted at each time step. Overall summaries include average median change in patient health status, total praxis, and total number of items in the commons.

The overall influence diagram is shown in Diagram 2. Starting in the southeast of the influence diagram, a patient's health status changes from week to week, influenced by the effectiveness of the treatment regimen, the possibility of occasional relapse, and random variation each week. The patient's health status is perceived by the clinician and patient during a clinical encounter, with some inaccuracy. The evaluation accuracy varies from one clinician-patient pair to another.

Based on the perceived health status, the treatment regimen may be changed during a clinical encounter. The selection of an effective treatment regimen depends on the selection efficiency of the clinician and patient, their ability to prefer an effective treatment regimen to of an ineffective one. Both selection efficiency and evaluation accuracy are driven by praxis, the knowledge available for treatment decision making. Praxis is the

result of the combination of phenotype response information—information about the phenotype of the patient—and individual response information—information about how well the patient is responding to the treatment regimen. Phenotype response information is affected by the engagement of the clinician, the engagement of the patient, the amount of shared knowledge in the commons, the quantity of records in the enhanced registry, and by whether the patient's care center practices previsit planning. Individual response information is also affected by the quantity of records in the enhanced registry and by previsit planning, and in addition is driven by the collection of individual response information by the patient. The collection of individual response information is determined by patient engagement, clinician engagement, and by the amount of shared knowledge in the commons.

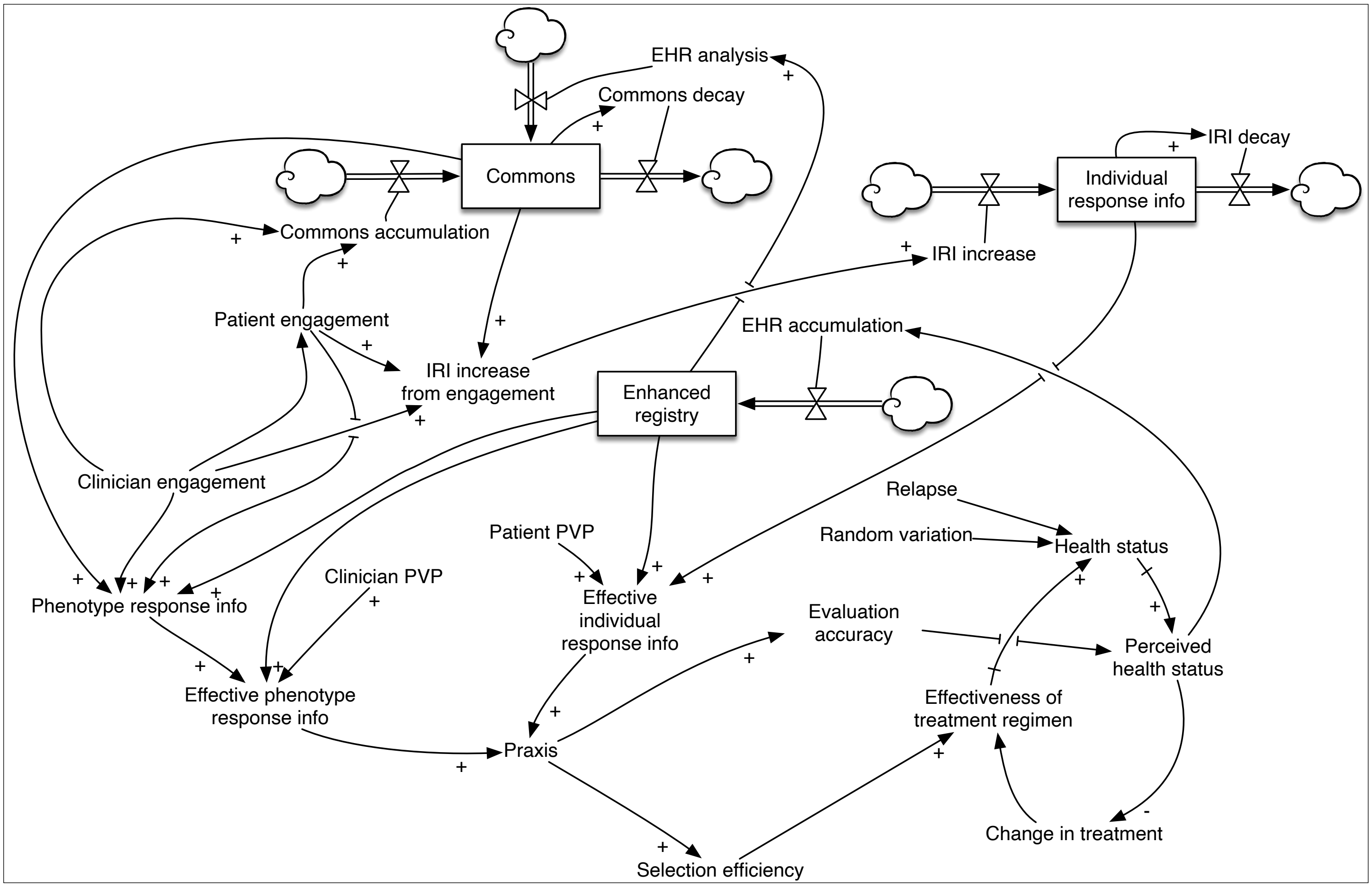

Diagram 2: the overall influence diagram

The enhanced registry accumulates records of clinican encounters. Shared knowledge accumulates in the commons as patients and clinicians contribute knowledge, and as the enhanced registry is periodically analyzed. Contribution to the commons varies, depending on the engagement of each patient and each clinician. The engagement of each patient changes over the course of the simulation, by his experiences during the clinical engagement, and by other patients he knows. The engagement of each clinician also changes over the course of the simulation, affected by other clinicians she knows.

5. Initialization

The model is designed to be generally applicable to multiple cases. Initialization is different for each scenario because the goal of simulation experiments is to understand the effects of initial conditions.

Patients

The learning network simulation can be used to model a learning network that does not yet exist, but that is planned or contemplated. Alternatively, the learning network simulation can be used to model an existing learning network: for example, when changes are being planned or contemplated. The configuration of new or existing is selected in the user interface, as shown in Screenshot 1. If the learning network does not yet exist, patients are enrolled over time as specified in the Patient Enrollment Submodel, described in Section 7.

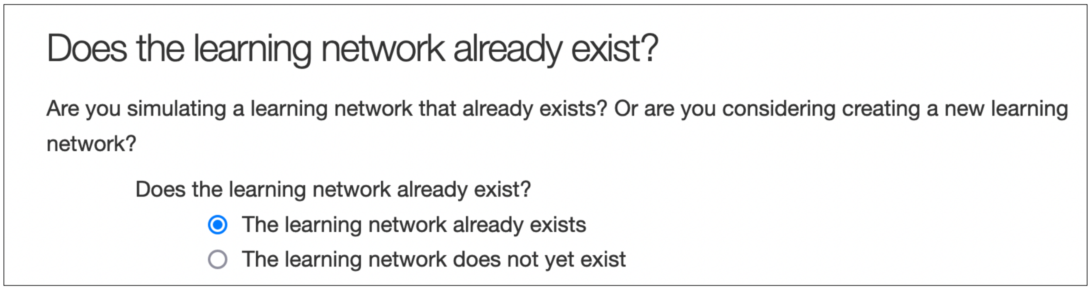

Screenshot 1: new learning network or existing one?

Every patient has an initial level of engagement with the learning network at the beginning of the simulation, one of unaware, aware, participating, contributing, and owning. The initial level of engagement of a patient is found by sampling from a categorical distribution, as shown in Equation 1.

$$PatientEngagement \sim Categorical(UnawareP, AwareP, ParticipatingP, ContributingP, OwningP) \quad (1)$$

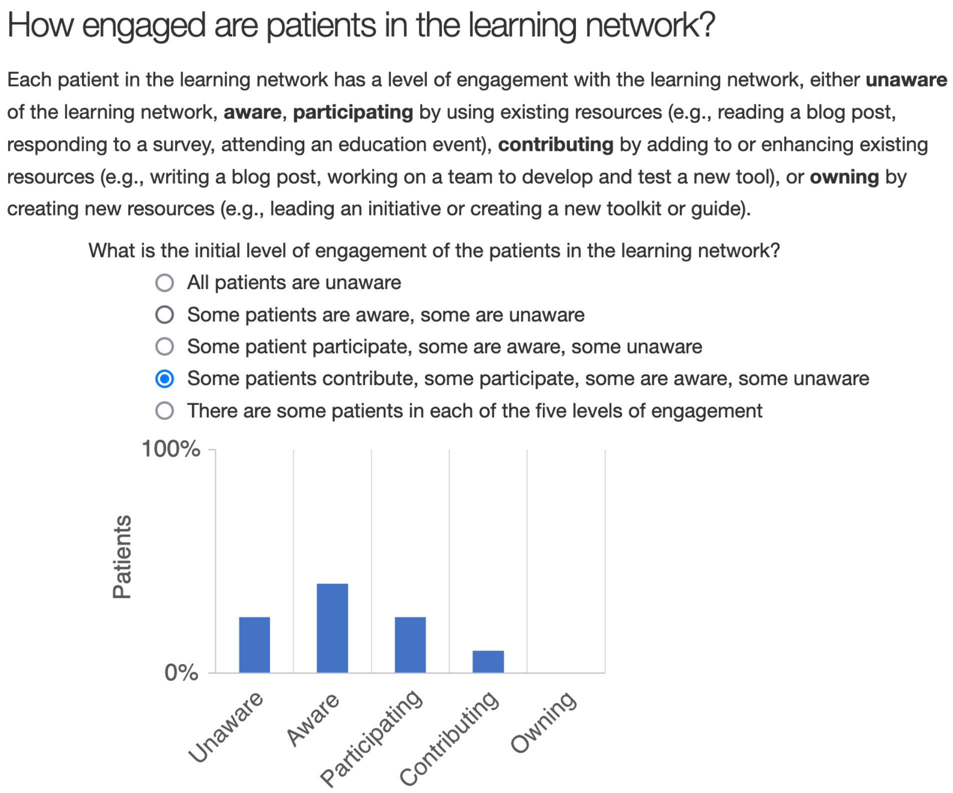

Screenshot 2: initial patient engagement

The probabilities of the initial patient engagements—the probability of unaware, aware, participating, contributing, or owning—are configured via the user interface, as shown in Screenshot 2.

Each patient has a phenotype, a random draw from the possible phenotypes. A patient's phenotype does not change over the course of the simulation. Screenshot 3 shows the initialization of the count of possible phenotypes.

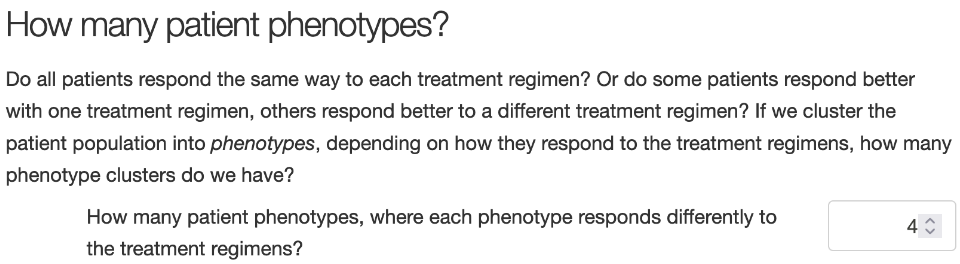

Screenshot 3: patient phenotypes

Each patient has an initial health status, modeled on a zero-to-one scale. Zero is a minimal state for the patient's health status. One is a maximal state, e.g., complete remission. Patient initial health status is drawn from a beta distribution. Equation 2 shows the initial health status for each patient.

$$InitialPHS \sim \begin{cases} Beta(InitialPHSalpha, InitialPHSbeta) \ if \ selected \\ Uniform(0,1) \ if \ selected \end{cases} \qquad (2)$$

The beta distribution of Equation 2 has the usual two shape parameters: alpha and beta, values common to all patients. Rather than directly specifying alpha and beta, the user controls the shape of the distribution by selecting a radio button option in the user interface, as shown in Screenshot 4. The radio button selected in Screenshot 4 determines the numeric value for initial health status alpha and initial health status beta of Equation 2, according to Table 1.

**Radio button**	**Initial health status alpha**	**Initial health status beta**
Mostly between 0.1 and 0.5 …	2.0	5.0
Mostly between 0.2 and 0.6 …	3.0	4.0
Mostly between 0.3 and 0.7 …	3.5	3.5
Mostly between 0.4 and 0.8 …	4.0	3.0
Mostly between 0.5 and 0.9 …	5.0	2.0

Table 1: possible values of alpha and beta for initial condition

There is a sixth radio button in Screenshot 4 that does not correspond to any of the entries in Table 1: *any condition between zero and one equally likely.* If the user selects that radio button, the lower condition of Equation 2 is used for the initial health status, a uniform distribution instead of a beta distribution. Every initial health status is drawn from a uniform distribution between zero and one.

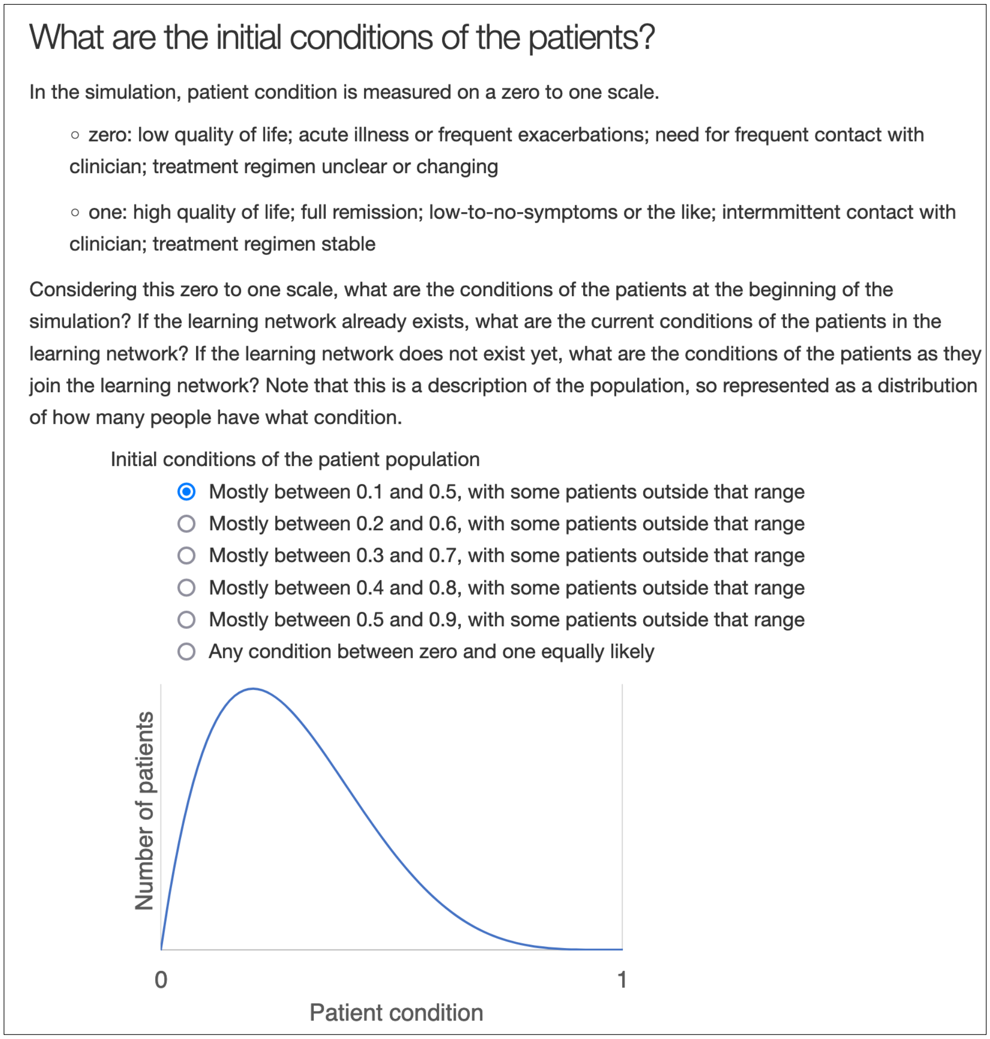

Screenshot 4: initial patient health status (labeled 'condition' in this screenshot)

Each patient has an initial level of individual response information, initial knowledge about their own response to possible treatments. The extent of a patient's knowledge about that response is modeled on a zero-to-one scale, with zero as no knowledge and one as perfect knowledge. For each patient, individual response information changes from week to week, with every simulation step. The initial level of individual response information for each patient is set in the user interface, as shown in Screenshot 5.

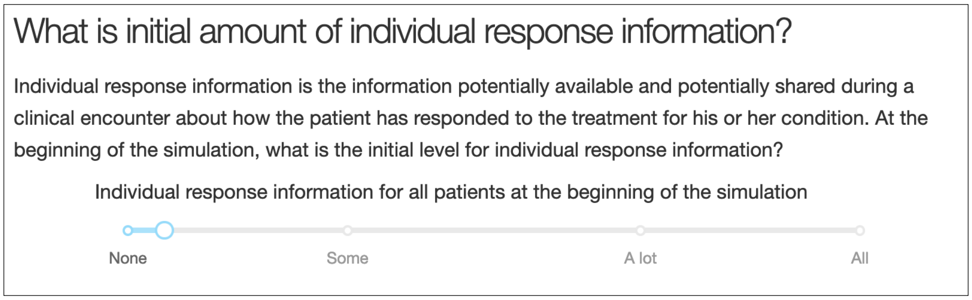

Screenshot 5: initial individual response information

Patients can affect engagement levels of other patients because influence: social connections. Each patient has influence connections to some other patients: connections because they are friends or enjoy some other personal relationships. Influenced connections are bidirectional: a connection between patients Zack and Giovanni will result in Zack influencing Giovanni, and also Giovanni influencing Zack.

The influence network is denser within a care center than across care centers. If Zack and Giovanni are patients of the same care center, and Joshua is a patient of a different care center, it is more likely that Zack and Giovanni share an influence link than do Zack and Joshua.

These influence connections do not change over the course of the simulation, except when patients enter or exit the learning network. For a static network—one in which no patients enter or exit the network during the simulation—the influence links are created at initialization, and remain constant over the course of the simulation. In other words, an influence link between two patients is a one-time draw from a Bernoulli distribution, as shown in Equation 3.

$$PatientInfluence_{p,q} \sim Bernoulli\left(PatientInfluenceProb_{p,q}\right) \tag{3}$$

The probability of an influence link between patients *p* and *q* depends on whether they receive care at the same care center, as shown in Equation 4.

$$PatientInfluenceProb_{p,q} \equiv \begin{cases} PatientInfluenceSameCC \; if \; CareCenter_p = CareCenter_q \\ PatientInfluenceDiffCC \; if \; CareCenter_p \neq CareCenter_q \\ 0 \; if \; p = q \end{cases} \tag{4}$$

The two probabilities in Equation 4—the probability of an influence link for patients at the same care center, and the probability of an influence link for patients at different care centers—are sufficient to configure patient influence links. But we found that these probabilities are not intuitive to users. So these two probabilities are specified indirectly in the user interface with the two dialogs shown in Screenshots 6 and 7.

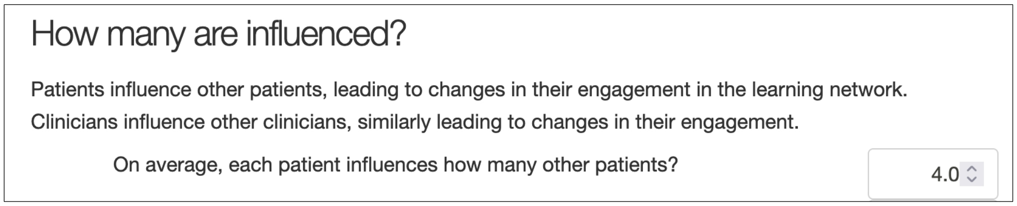

Screenshot 6: density of patient influence links

Screenshot 6 specifies the average numeric density of patient influence links. The average numeric density of patient influence links drives the total count of influence links as shown in Equation 5. The 2 in the denominator of Equation 5 reflects the fact that influence links are bidirectional.

$$PatientInfluenceCount \equiv \frac{PatientCount * PatientInfluenceDensity}{2} \tag{5}$$

There is a potential patient influence link between every pair of patients. Some of these potential patient influence links are within a care center, when the two patients receive care at the same care center. Some of these potential patient links cross from one care center to another, when the two patients receive care at different care centers. Given the partition of the patient population into care centers (described in Section 3), the model counts how many potential patient influence links are with a single care center, and how many potential patient influence links cross care center boundaries. These potential influence link counts are used as the denominators of Equations 6 and 7.

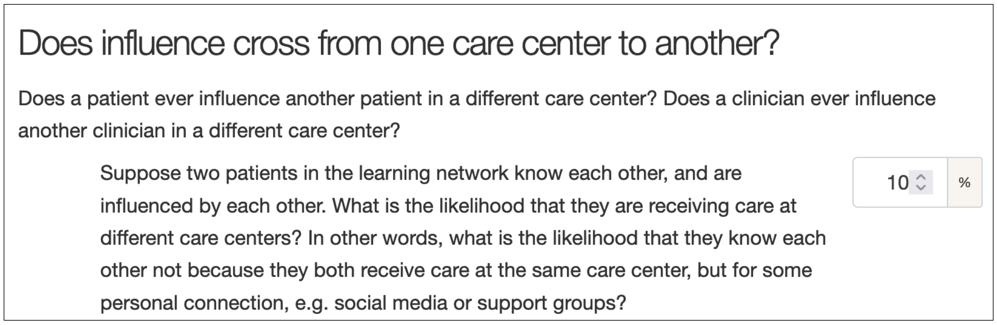

Screenshot 7: proportion of influence links that cross care center boundaries.

Screenshot 7 specifies what percentage of the patient influence links actually cross care center boundaries. Typically this percentage is less than would be expected from the relative counts of potential patient influence links, because influence is denser within a care center than across care centers

Equation 6 determines the probability of a patient influence link between two patients in the same care center. Similarly, Equation 7 determines the probability of a patient influence link between two patients at different care centers.

$$PatientInfluenceSameCC \equiv \frac{PatientInfluenceCount * (1 - PatientCrossCareCenterProp)}{PatientInfluenceLinkCountWithinCareCenter} \tag{6}$$

$$PatientInfluenceDiffCC \equiv \frac{PatientInfluenceCount * PatientCrossCareCenterProp}{PatientInfluenceLinkCountAcrossCareCenters} \tag{7}$$

It is possible to specify incoherent configuration values in Screenshots 6 and 7. For example, if a user enters an overly large value in the dialog of Screenshot 6, it could imply an influence network with more links than the potential links among all the patients. The model catches this error, and the user interface reports it.

As another example of incoherent configuration, suppose there is only one care center. An entry of any percentage less than 100% in the dialog of Screenshot 7 is incoherent. The model catches this error, and the user interface reports it.

The probability of an influence link between two patients is a bit more complex if the learning network is dynamic, if patients can arrive in the learning network later. That complexity is not detailed here. But the result is that the model determines probabilities for links among newly arriving patients such that the values specified in Screenshots 6 and 7 are valid.

Clinicians

As with patients, each clinician has an initial **level of engagement** with the learning network at the beginning of the simulation, one of unaware, aware, participating, contributing, and owning. The initial level of engagement of a clinician is found by sampling from a categorical distribution, as shown in Equation 8.

$$ClincianEngagement_{\square} \sim Categorical(UnawareC, AwareC, ParticipatingC, ContributingC, OwningC) \tag{8}$$

The probabilities of the initial clinician engagements—the probability of unaware, aware, etc.—are configured via the user interface, as shown in Screenshot 8.

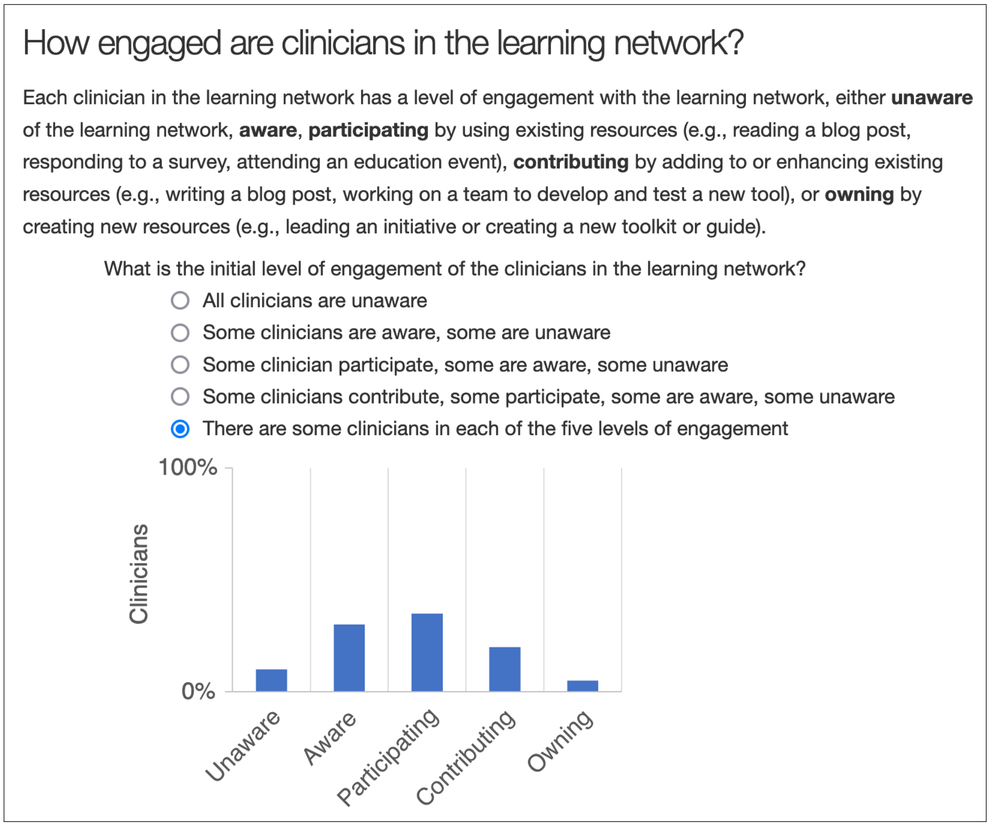

Screenshot 8: initial clinician engagement

There is also an influence network among clinicians, entirely separate from the patient influence network. Each clinician has influence connections to some other clinicians: colleagues, friends from medical school, or other personal relationships. As with patient influences, clinician influence connections are bidirectional: a connection between clinicians Dr. Morgan and Dr. Heath will result in Dr. Morgan influencing Dr. Heath, and also Dr. Heath influencing Dr. Morgan.

As with patient influence, the clinician influence network is denser within a care center than across care centers. If Dr. Morgan and Dr. Heath are clinicians practicing at the same care center, and Dr. Lurie is a clinician practicing at a different care center, it is more likely that Dr. Morgan and Dr. Heath share an influence link than do Dr. Morgan and Dr. Lurie. Note that we do not model the (perhaps common) situation of a clinician providing care at multiple care centers.

As with patient influence, these clinician influence connections do not change over the course of the simulation. The influence links are created at model initialization, and remain constant for the entire simulated time. In other words, an influence link between two clinicians is a one-time draw from a Bernoulli distribution, as shown in Equation 9.

$$ClinicianInfluence_{c,d} \sim Bernoulli(ClinicianInfluenceProb_{c,d}) \quad (9)$$

The probability of an influence link between clinicians *c* and *d* depends on whether they practice at the same care center, as shown in Equation 10.

$$ClinicianInfluenceProb_{c,d} \equiv \begin{cases} ClinicianInfluenceSameCC \; if \; CareCenter_c = CareCenter_d \\ ClinicianInfluenceDiffCC \; if \; CareCenter_c \neq CareCenter_d \\ 0 \; if \; c = d \end{cases} \quad (10)$$

The two probabilities *ClinicianInfluenceSameCC* and *ClinicianInfluenceDiffCC* are sufficient to configure clinician influence links. But as with their patient counterparts, we found that these probabilities are not intuitive to users. So these two probabilities are specified indirectly in the user interface, with the dialogs shown in Screenshots 9 and 10.

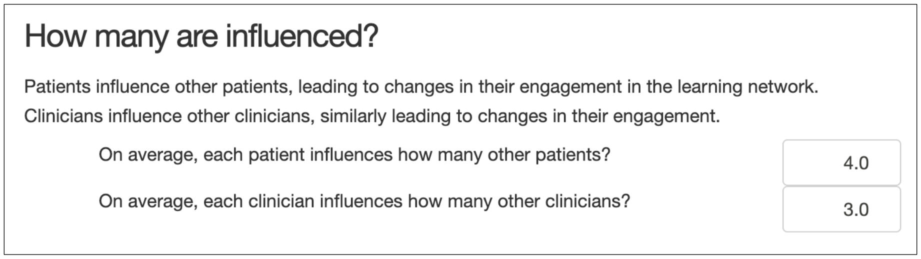

Screenshot 9: density of clinician influence links

Screenshot 9 specifies the numeric density of both patient influence links and clinician influence links: patient influence density (as described above), and clinician influence desnity. The total count of clinician influence links is determined in Equation 11. The 2 in the denominator reflects that fact that influence links are bidirectional.

$$ClinicianInfluenceCount \equiv \frac{ClinicianCount * ClinicianInfluenceDensity}{2} \quad (11)$$

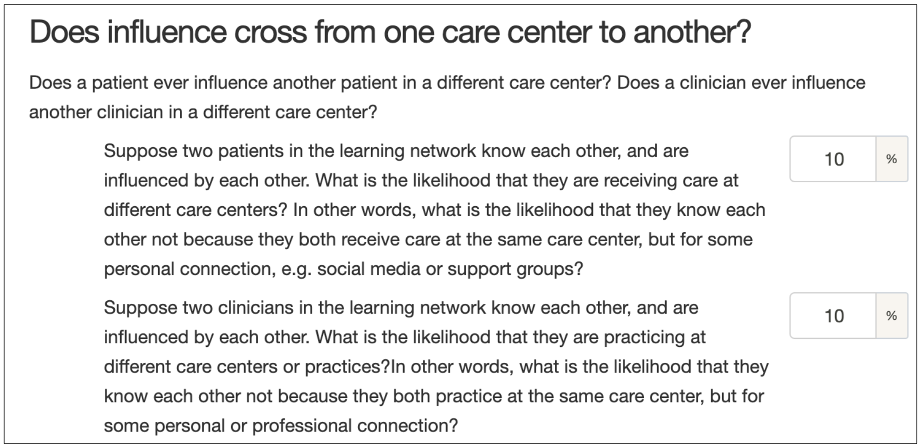

Screenshot 10: proportion of influence that crosses care center boundaries

Screenshot 10 specifies what percentage of the clinician links cross care center boundaries. (Also shown is the percentage of patient influence links that cross care centers, described above.)

As with patients, there is a potential clinician influence link between every pair of clinicians. Some of these potential links are within a care center, when the two clinicians practice at the same care center. Some of these potential links cross from one care center to another, when the two clinicians practice at different care centers. Given the partition of clinicians into care centers (described in Section 3), the model counts how

many potential clinician influence links are within a single care center, and how many potential clinician influence links cross care center boundaries.

Equation 12 determines the probability of a clinician influence link between two clinicians practicing in the same care center.

$$ClinicianInfluenceSameCC \equiv \frac{ClinicianInfluenceCount * (1 - ClinicianCrossCareCenterProp)}{ClinicianInfluenceLinkCountWithinCareCenter} \quad (12)$$

Similarly, Equation 13 determines the probability of a clinician influence link between two clinicians practicing at different care centers.

$$ClinicianInfluenceDiffCC \equiv \frac{ClinicianInfluenceCount * ClinicianCrossCareCenterProp}{ClinicianInfluenceLinkCountAcrossCareCenters} \quad (13)$$

As with patients, it is possible to specify incoherent values in Screenshots 9 and 10. The model catches any incoherent values, and the user interface reports the error.

Medical Condition

The medical condition under treatment has a natural history, a change from one week to the next, apart from the effects of treatment, random variation, or occasional relapse. Natural history change is a constant for the medical condition simulated. It is measured on a scale of -1.0 to 1.0 per week, although in practice a value of 0.02 is a rapid improvement and a value of -0.02 is a rapid decline. It is set by the user in the user interface with the dialog shown in Screenshot 11.

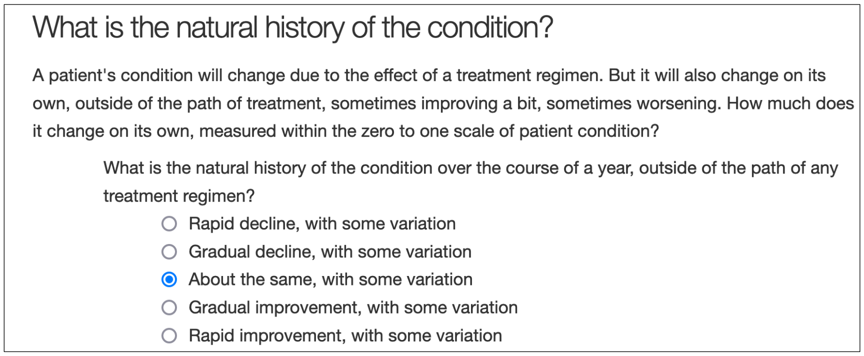
What is the natural history of the condition?

A patient's condition will change due to the effect of a treatment regimen. But it will also change on its own, outside of the path of treatment, sometimes improving a bit, sometimes worsening. How much does it change on its own, measured within the zero to one scale of patient condition?

What is the natural history of the condition over the course of a year, outside of the path of any treatment regimen?

- ○ Rapid decline, with some variation
- ○ Gradual decline, with some variation
- ◉ About the same, with some variation
- ○ Gradual improvement, with some variation
- ○ Rapid improvement, with some variation

Screenshot 11: natural history change

The five radio button settings in Screenshot 11 correspond to the values for natural history change shown in Table 2.

Radio button	Natural history change value
Rapid decline	-0.02
Gradual decline	-0.01
About the same	0.00
Gradual improvement	0.01

Radio button	Natural history change value
Rapid improvement	0.02

Table 2: possible values of natural history change

For any medical condition, there is an an inherent degree of randomness in change of patient health status, the normal ups and downs that any patient experiences, aside from the effect of the treatment and the natural history of the medical condition. Like the natural history change, random variation is measured on a scale from -1.0 to 1.0, with smallish typical values. Random variation for any particular patient is modeled by a weekly draw on a truncated normal distribution, a normal truncated to allow values no larger than 1.0 and no smaller than -1.0, as shown in Equation 14.

$$RandomVariation \sim TruncNormal(0, RandomVariationSD, -1, 1) \quad (14)$$

The standard deviation of random variation is set in the user interface, via the dialog shown in Screenshot 12.

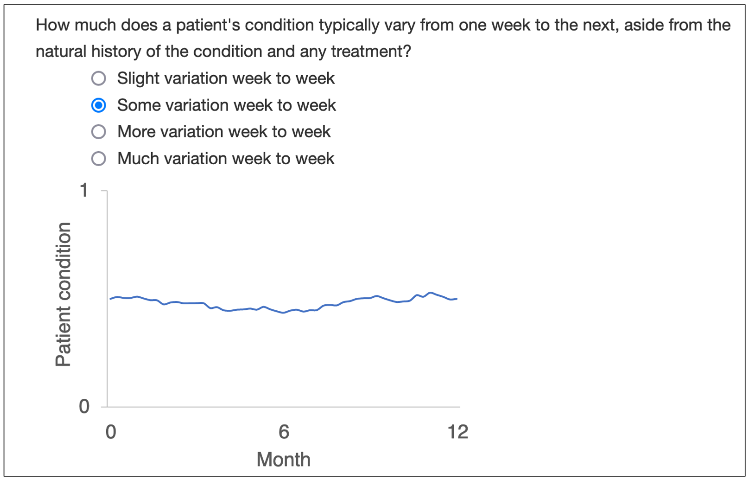

Screenshot 12: random variation standard deviation

The four radio button settings in Screenshot 12 correspond to the values for **Random variation standard deviation** shown in Table 3.

Radio button	Random variation SD value
Slight variation	0.005
Some variation	0.01
More variation	0.02
Much variation	0.04

Table 3: possible values of random variation standard deviation

Sometimes a patient will suffer a relapse, with a suddenly worsening condition. Relapse is modeled as a random variation that mostly does not occur but occasionally does, as shown in Equation 15.

$$Relapse \sim Bernoulli\left(\frac{1}{RelapsePeriod}\right) * RelapseExtent \quad (15)$$

Relapse period is the average duration between a patient relapsing. The period is only an average: it is quite possible for the relapse period to be 1 year, but for an unlucky patient to relapse one week, again a few weeks later, and once again a few weeks after that.

Relapse period is specified with a slider in the user interface, as shown in Screenshot 13.

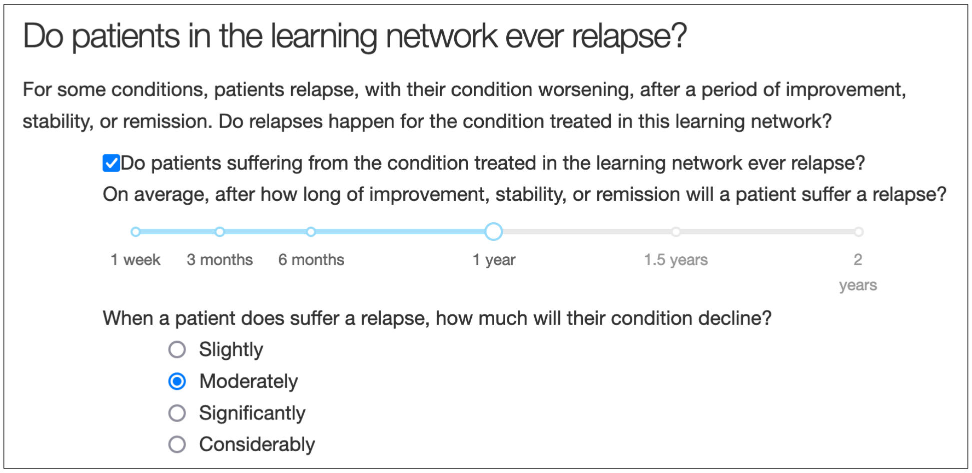

Screenshot 13: patient relapse

Relapse extent is the amount that a patient's health status relapses when a relapse occurs. It is measured on the health status zero-to-one scale. Extent is specified in the user interface via radio buttons, also shown in Screenshot 13. Each of four radio button settings corresponds to a numeric value for relapse extent, detailed in Table 4.

Radio button	Random extent value
Slightly	0.05
Moderately	0.1
Significantly	0.2
Considerably	0.5

Table 4: possible values of relapse extent

There are a small number of alternative treatment regimens. The effectiveness of a treatment regimen varies by phenotype: an effective treatment regimen for one phenotype may be ineffective for another. The count of treatment regimens is settable in the user interface, as shown in Screenshot 14.

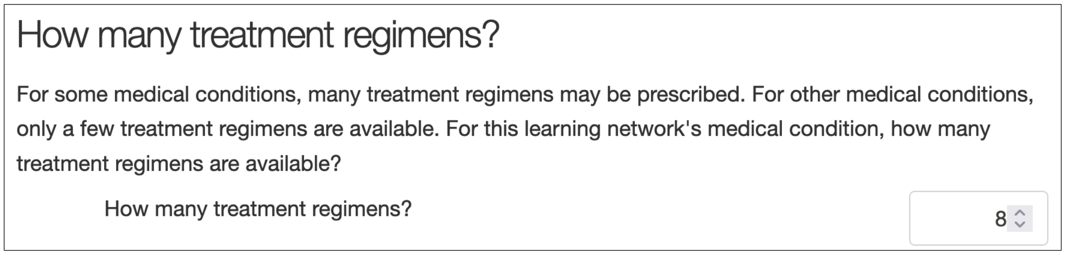

Screenshot 14: treatment regimens

On simulation initialization, each treatment regimen is assigned an effectiveness for each phenotype. The effectiveness is measured as the weekly increase in patient health status when a patient of that phenotype is treated with the treatment regimen. Since patient health status is measured on a zero-to-one scale, the effectiveness of a treatment regimen for a particular phenotype is a small quantity, typically something like 0.02 or maybe -0.01.

Regimen effectiveness is drawn from a normal distribution, with constant mean and standard deviation across every combination of treatment regimen and phenotype, as described in equation 16. The distribution is truncated to be no less than -1 and no more than 1, the maximum possible effectiveness.

$$Effectiveness \sim TruncNormal(EffectivenessMean, EffectivenessSD, 1, -1) \tag{16}$$

The mean and standard deviation of treatment regimen effectiveness are specified indirectly, in the user interface, as the effectiveness of the typical best and worst treatment regimen. See Screenshot 15.

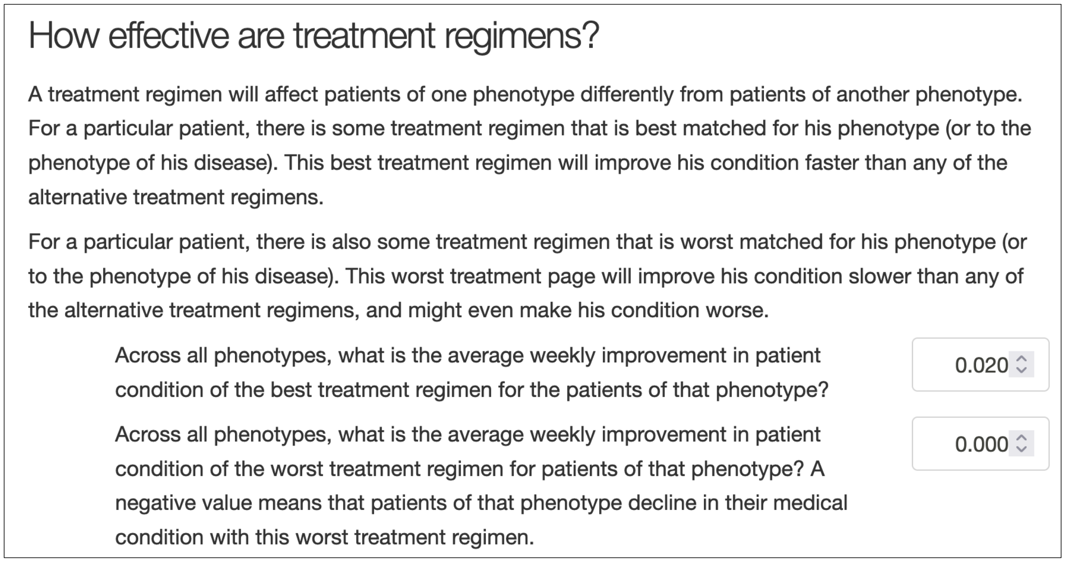

Screenshot 15: treatment regimen effectiveness

Effectiveness mean is the simple average of best case and worse case, as shown in Equation 17.

$$EffectivenessMean \equiv \frac{MeanBest + MeanWorst}{2} \tag{17}$$

Effectiveness standard deviation is estimated empirically at simulation start by drawing from a set of standard normal distributions, with the cardinality of the set as the count of treatment regimens, and then noting the maximum and minimum draw in that set. These set draws are repeated a large number of times, to estimate mean max and mean min, and then using max and min to scale the standard deviation appropriately.

Medical condition knowledge is how much is known about the condition treated in the learning network. For some medical conditions, much is known about effective treatments. For other medical conditions, less is known. The state of knowledge about the medical condition is modeled on a zero-to-one scale, with zero for ultra-rare diseases and other conditions for which little is known, and one for conditions like pneumonia where much is known. Medical condition knowledge is set as a slider in the user interface, as shown in Screenshot 16.

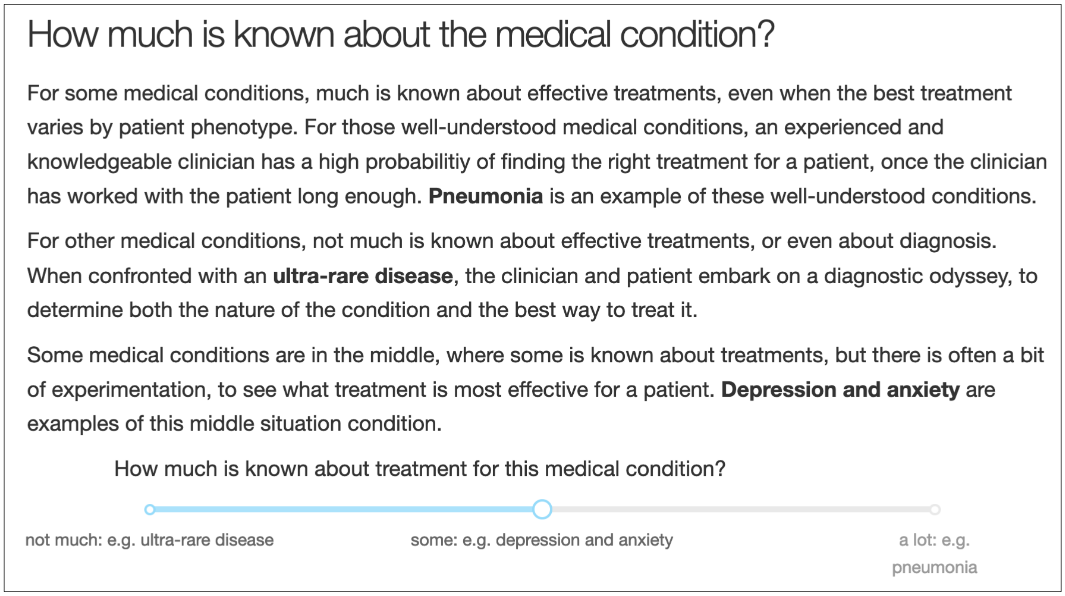

Screenshot 16: medical condition knowledge

Care Centers

A learning network generally spans multiple care centers. We model the learning network as having one or more care centers, each care center employing some number of clinicians, and treating some number of patients. The number of care centers—and the count of clinicians and patients in each—is configured in the user interface, as shown in Screenshot 17. As an alternative to filling out the table in the user interface, the user can create a spreadsheet with the same information, and then upload the spreadsheet.

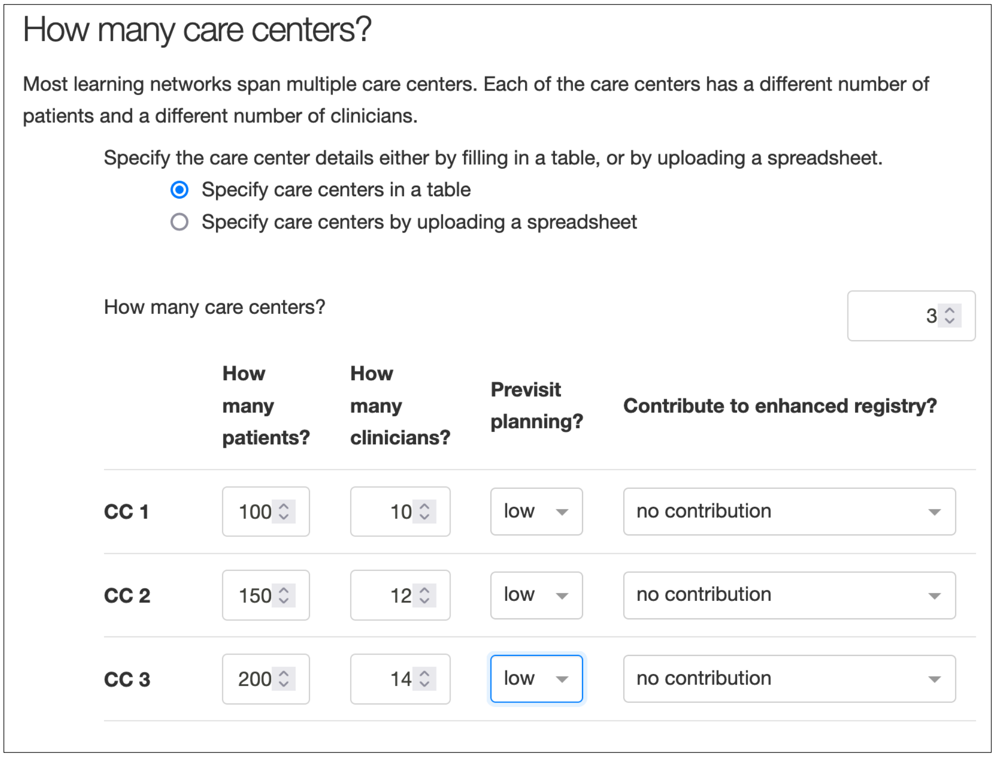

Screenshot 17: the care centers

In the simulation, when a patient enrolls, he or she is assigned a clinician. This clinician assignment is a simplification of reality. A newly enrolled patient may already be treated by a clinician for some time, long

before enrollment in the learning network. The model ignores these details of pre-existing clinician assignment.

In the model, patient assignment to a clinician occurs randomly: on enrollment, a patient is assigned to one of the clinicians in the care center, a random choice over all the clinicians practicing at the care center. Again, this random assignment is a simplification of the reality. For example, some clinicians may have no additional availability, and so are not currently taking any new patients. The model ignores these details.

As an alternative to random assignment, the user can specify that clinicians are assigned successively, so the first enrolled patient will be assigned to the first clinician, the second patient to the second clinician, and so on, wrapping back around to the first clinician after a patient has been assigned to the last clinician. The specification of random or successive is a choice in the user interface, shown in Screenshot 18.

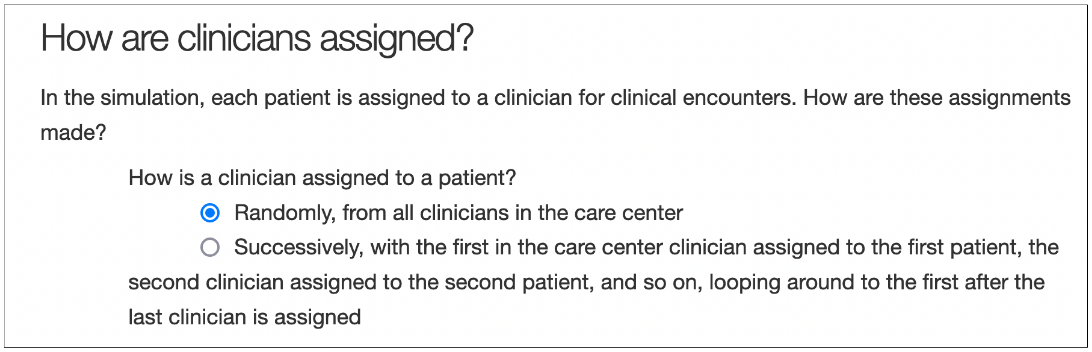

Screenshot 18: assigning clinicians to patients

We have never noticed a difference in model behavior from the choice of patient assignment mechanism.

The Commons

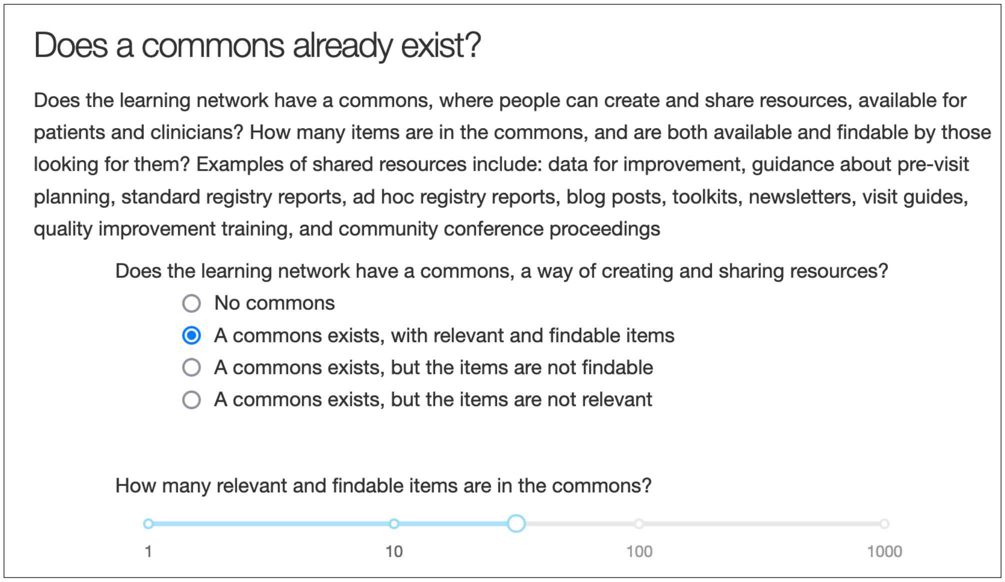

Screenshot 19: initial size of the commons

If the learning network simulation is modeling a learning network that is planned or contemplated, the commons may be initialized with nothing, no shared knowledge at the beginning. Or if an existing learning network is simulated, the commons may be simulated with an initial quantity of shared knowledge. Either situation can be set in the user interface, as shown in Screenshot 19.

The Enhanced Registry

A care center may employ an enhanced registry to upload, aggregate, and use clinical data collected as part of care. In the simulation, the enhanced registry may initially have no records, or it may have some quantity of records, determined in the user interface, as shown in Screenshot 20.

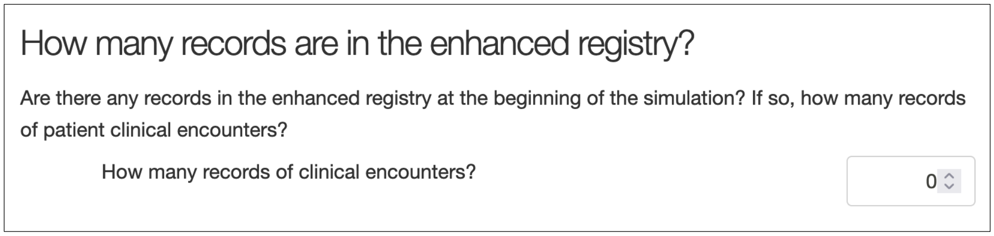

Screenshot 20: initial size of enhance registry

6. Input Data

The model does not use input data to represent time-varying processes.

7. Submodels

There are five submodels:

- Commons submodel
- Update Patient submodel
- Update Clinician submodel
- Enhanced Registry submodel
- Patient Arrive submodel

These five submodels are of very different sizes; the **Update Patient** submodel is larger than the other four combined, and includes several sub-submodels, as described below.

Commons submodel

The **Commons** submodel decreases the amount of shared knowledge in the commons by a constant decay proportion every week. The weekly decay proportion is determined from the annual decay proportion, via Equations 18 and 19.

$$DecayProp \equiv 1 - 0.5^{\frac{1}{SharedKnowledgeHalfLife}} \tag{18}$$

$$SharedKnowledgeHalfLife \equiv \frac{365.25 * \ln(0.5)}{7 * \ln(1 - SharedKnowledgeOneYearDecay)} \tag{19}$$

The annual decay proportion is set in the user interface, as shown in Screenshot 21.

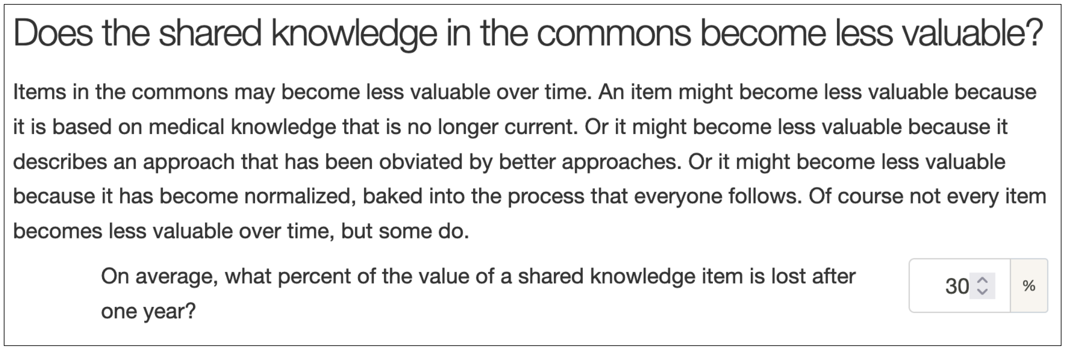

Screenshot 21: shared knowledge decay

Update Patient submodel

The **Update Patient** submodel consists of six sub-submodels, executed in order with each execution of the **Update Patient** submodel for a particular patient agent:

1. Adjust Health Status sub-submodel
2. Update Individual Response Information sub-submodel
3. Patient Commons Contribution sub-submodel
4. Clinical Encounter sub-submodel
5. Patient Transition sub-submodel
6. Patient Leave Cohort sub-submodel

Note that while all of these sub-submodels are executed every week, some of the sub-submodels only make a difference conditionally. For example, the **Patient Leave Cohort** sub-submodel is executed every week for each patient in the simulation, to see if the patient agent will leave the cohort, and end his care in the learning network. But a particular patient will actually leave the cohort at most once during a simulation run. Mostly **Patient Leave Cohort** has no effect.

Adjust Health Status sub-submodel

The **Adjust Health Status** sub-submodel changes the health status of a patient every week. The health status of a patient is modeled on a zero-to-one scale. The health status increases or decreases every week, and is constrained within the interval [0.0, 1.0]. The change in health status is the sum of four sources of change, as shown in Equation 20.

$$ChangeInHealthStatus \equiv NaturalHistoryChange + TreatmentEffect + RandomVariation + Relapse \quad (20)$$

Natural history change is the usual weekly change in health status for the medical condition simulated. It is modeled as a constant for a particular medical condition and simulation run. The constant has a value on the zero-to-one scale, typically a value close to zero, e.g. 0.01 or -0.02. Natural history change is described with the initialization parameters in Section 5.

Treatment effect models the effect of the treatment regimen on this particular patient. Patient-clinician dyads are attempting to apply a treatment regimen with a positive treatment effect, but doing so in the face of uncertainty about the true effect of a treatment regimen. Sometimes they are successful. Treatment effect is described in more detail in the **Clinical Encounter** sub-submodel.

Random variation models the normal ups and downs that any patient experiences, aside from the effect of the treatment and the natural history of the medical condition. Random variation is modeled on a zero-to-one

scale, with values that are typically close to zero. It is modeled by a weekly draw for each patient on a truncated normal distribution, a normal truncated to allow no values larger than 1.0 or smaller than -1.0, as shown in Equation 21.

$$RandomVariation \sim TruncNormal(0, RandomVariationSD, -1, 1) \tag{21}$$

The standard deviation of the random variation is an initialization parameter, described in Section 5.

Relapse models the situation when a patient suffers a relapse, a sudden worsening of his health status. Relapse is modeled as a random variation that mostly does not occur but occasionally does, as shown in Equation 22.

$$Relapse \sim Bernoulli\left(\frac{1}{RelapsePeriod}\right) * RelapseExtent \tag{22}$$

Relapse period is the average duration between a patient relapsing. The period is only an average: it is quite possible for the relapse period to be 1 year, but for a particular patient to relapse one week, again a few weeks later, and once again a few weeks after that. Relapse period is an initialization parameter, described in Section 5.

Relapse extent is the amount that a patient's health status declines during a relapse. It is measured on the health status zero-to-one scale. Relapse extend is an initialization parameter, described in Section 5.

Update Individual Response Information sub-submodel

Each patient in the model has some knowledge about their own response to the treatments. The extent of a patient's knowledge about that response is modeled as the individual response information, represented on a zero-to-one scale, with zero as no knowledge and one as perfect knowledge. For each patient, individual response information changes from week to week, with every simulation step. The **Update Individual Response Information** submodel changes the individual response information.

Individual response information changes each week for each patient in two directions, as shown in Equation 23. Individual response information increases, due to the enagement of the patient and clinician. And individual response information decays.

$$\begin{aligned} &ChangeInIndividualResponseInformation_{\square} \\ &\quad \equiv IndividualResponseInformationIncrease_{\square} \\ &\quad - IndividualResponseInformationDecay_{\square} \end{aligned} \tag{23}$$

The increase in individual response information is driven by the engagement of the patient and his or her clinician, and also by the amount of contents in the commons. Even with nothing in the commons, an engaged patient and engaged clinician will increase the individual response information. The amount of content in the commons serves to accelerate the increase, as shown in Equation 24.

$$\begin{aligned} &IndividualResponseInformationIncrease_{\square} \\ &\quad \equiv IRIincreaseDueToEngagement * (CommonsAccelerationIRI + 1) \end{aligned} \tag{24}$$

IRIincreaseDueToEngagement—the increase in individual response information due to the engagement of patient and clinician—is the product of the weighted mean of engagement and the maximum response

increase speed, as shown in Equation 25. Maximum response increase speed is a small constant of the fastest that individual response information can increase due to engagement alone.

$$IRIincreaseDueToEngagement \equiv EngagementWeightedMean * MaxIRIincreaseSpeed \tag{25}$$

The weighted mean of engagement for a patient is the mean of patient engagement and clinician engagement, both expressed as quantities on a zero-to-one scale. The mean is weighted by the constant of the degree of patient tilt, the degree to which the patient engagement is preferred to clinician engagement, as shown in Equation 26. Note that the clinician engagement amoun in Equation 26 is the engagement of the unique clinician who is providing care for the patient.

$$EngagementWeightedMean \equiv PatientEngagementAmount * IRIengagementTilt + ClinicianEngagementAmount * (1 - IRIengagementTilt) \tag{26}$$

The patient engagement amount in Equation 26 is the patient's categorical engagement level, translated into a numeric amount. The translation is set by in the user interface, as shown in Screenshot 22.

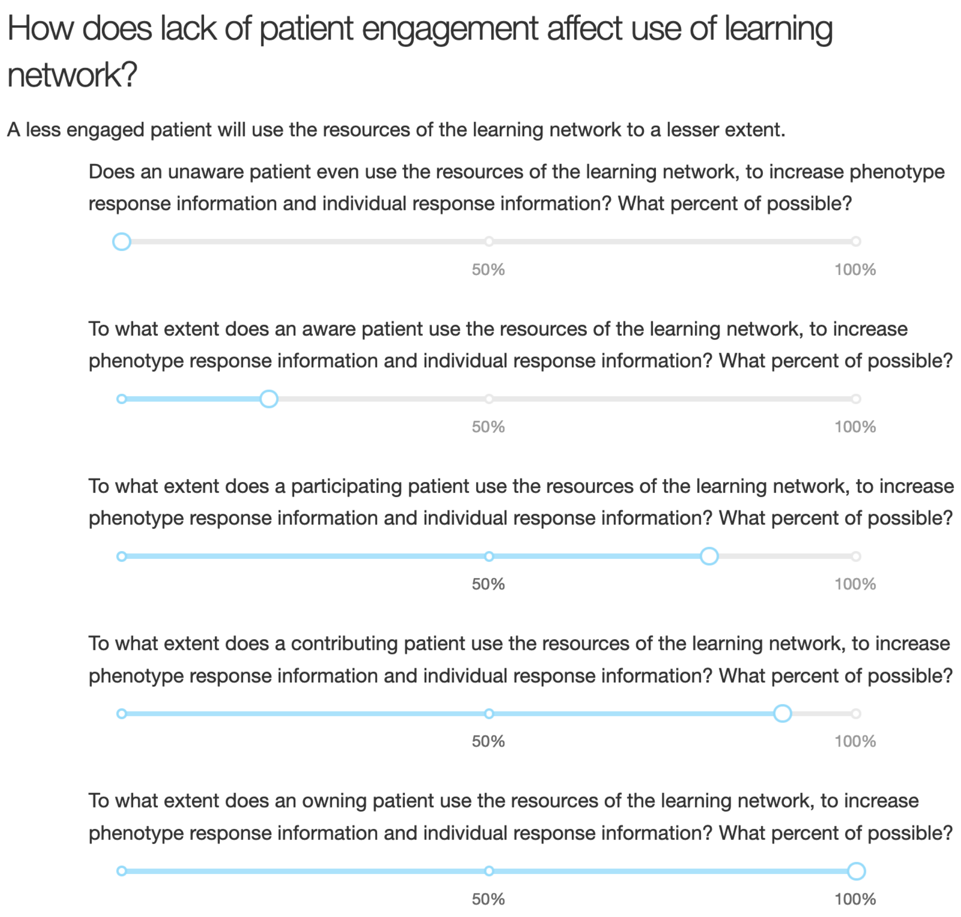

Screenshot 22: the effect of lack of patient engagement

Similarly clinician engagement amount in Equation 26 is the clinician's engagement level, translated into a numeric amount, as shown in Screenshot 23.

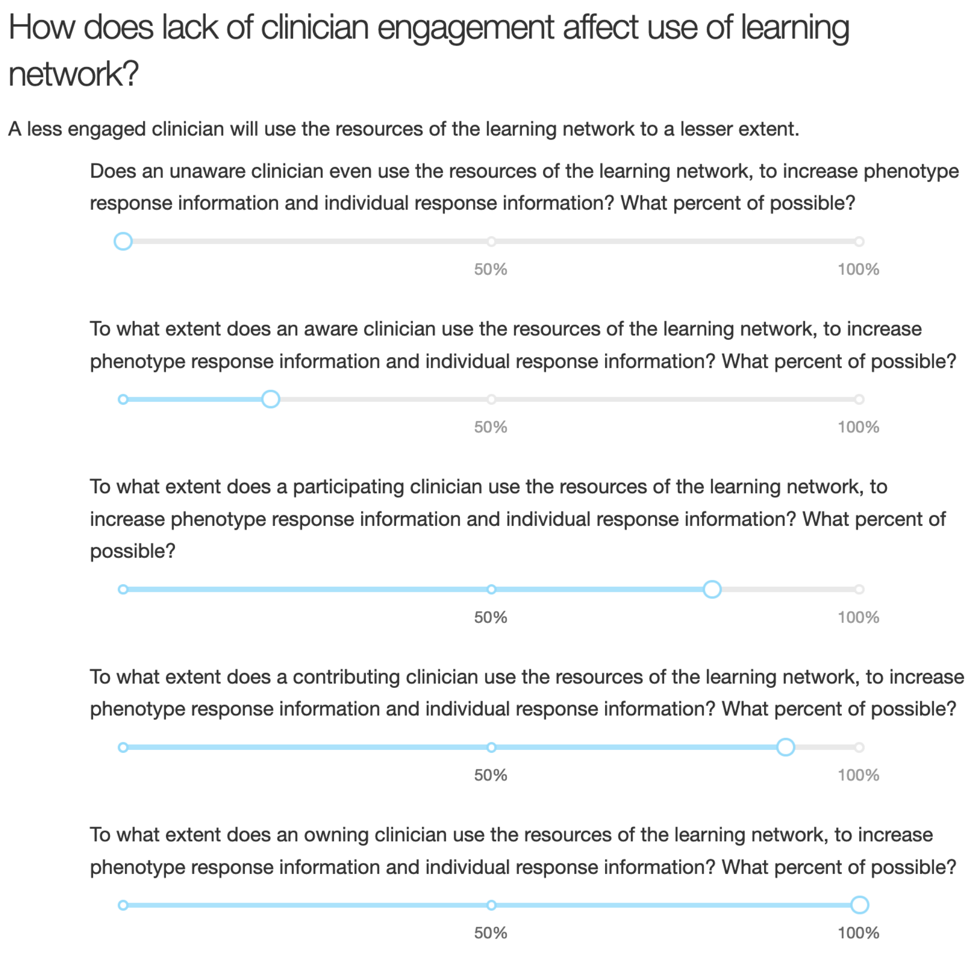

Screenshot 23: the effect of lack of clinician engagement

The default values in the user interface are listed in Table 5.

Engagement level	Engagement amount
Unaware	0.00
Aware	0.25
Participating	0.50
Contributing	0.75
Ownging	1.00

Table 5: mapping engagement level to engagement amount

IRI engagement tilt in Equation 26 is the degree to which the weighted mean is of engagement is weighted to the patient or to the clinician. It is represented on a zero-to-one scale, and is set in the user interface, as shown in Screenshot 24.

It might seem surprising that the engagement of the clinician affects the individual response information even on the weeks between a clinical encounter. The engagement of a clinician has a lasting effect on her patients. For example, an engaged clinician can encourage her patient to collect daily measurements of his own condition, and that encouragement affects his behavior for some weeks.

The maximum IRI increase speed is a constant. The simulation uses a value of 0.03333 for this constant. This represents a maximum increase rate that would allow a patient to grow individual response information from

nothing (zero) to complete (full) in 30 weeks, with full engagement of both patient and clinician, nothing in the commons, and not considering the effect of individual response information decay.

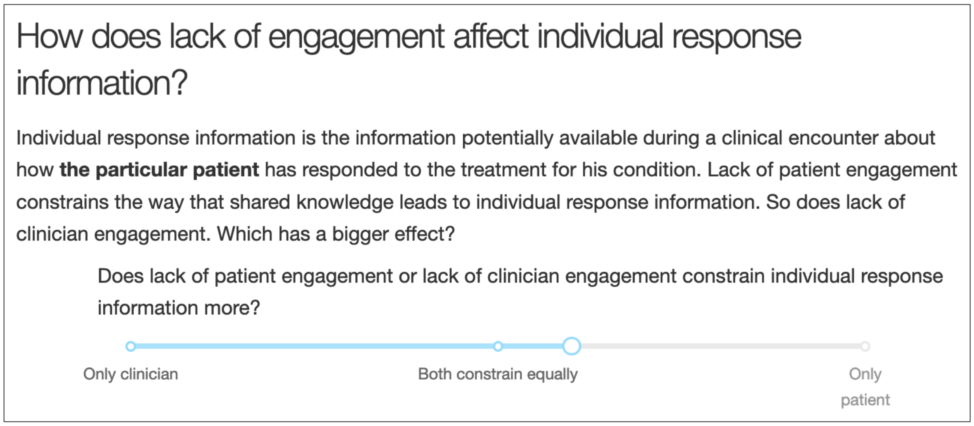

Screenshot 24: IRI engagement tilt

Shared knowledge in the commons increases the pace of increase of individual response information. The amount of that increase in pace is shown in Equation 24 as commons acceleration. Each additional unit of shared knowledge increases the amount of acceleration by a small increment, subject to decreasing returns. Equation 27 defines the commons acceleration as some proportion of the maximum acceleration.

$$CommonsAccelerationIRI \equiv MaximalAccelerationIRI * MaximalAccelerationIRIprop \quad (27)$$

The maximimal acceleration is the maximum extent that the commons accelerates the accumulation of individual response information, even with an unlimited quantity of shared knowledge in the Commons. Maximmal acceleration is a constant between zero and one, and set in the user interface, as shown in the lower slider in Screenshot 25. Note that the maximum possible value in Screenshot 25 is 100%: 1.0, and so at most the Commons can double the pace of accumulating individual response information.

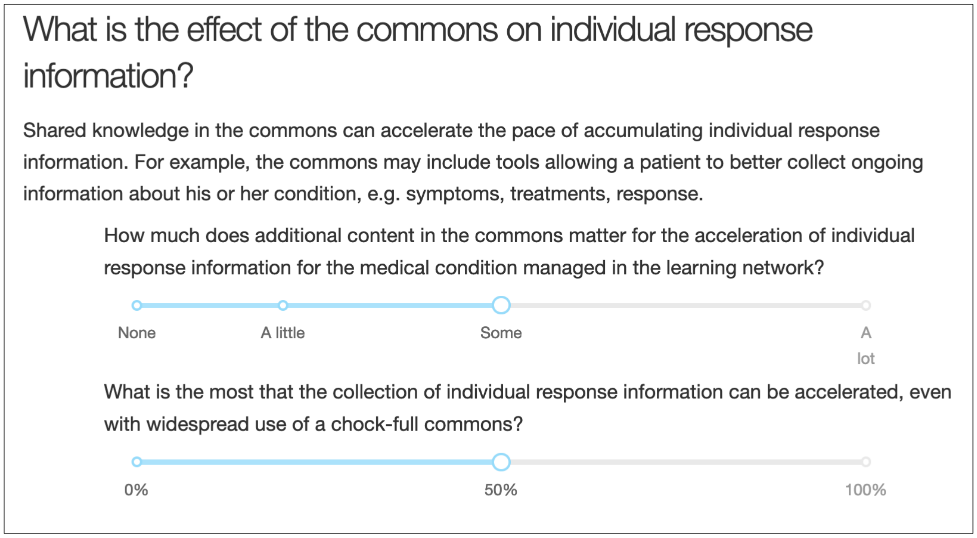

Screenshot 25: The effect of the commons on individual response information

The proportion of maximal acceleration in Equation 27 is the proportion of the maximum acceleration caused by the current quantity of shared knowledge in the Commons. The proportion is a quantity between zero and

one, zero when the Commons is empty, and one when the Commons is full. That quantity is defined in Equation 28, as a function of what the acceleration would be, if it was not limited by any maximum, but instead grew arbitrarily great with arbitrarily large quantities in the Commons. The would-be acceleration is defined in units of the maximimal acceleration, so if would-be acceleration is 2 (e.g.), there is enough in the Commons that acceleration would be twice as large as the maximum possible, if acceleration was not constrained by the maximum.

$$MaximalAccelerationIRIprop \equiv \frac{2}{1 + e^{-2*WouldBeAccelerationIRI}} - 1 \tag{28}$$

Equation 28 creates the decreasing returns effect, as shown by the graph in Figure 1.

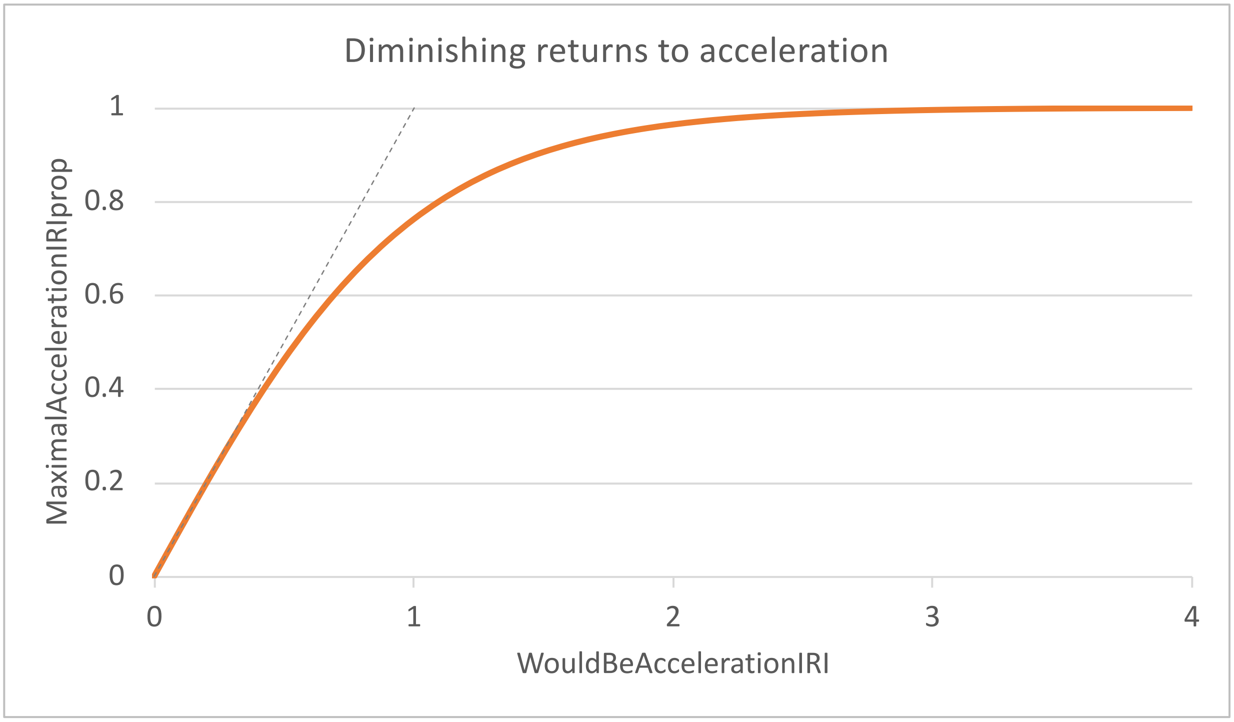

Figure 1: translating *WouldBeAcceleration* to *MaximalAccelerationProp*

The would-be acceleration is defined in Equation 29, as the product of the number of items in the Commons and the would-be acceleration of each individual item in the commons. The would-be acceleration of each individual item in the Commons can be interpreted as the acceleration accomplished by the first item in the Commons, before the onset of any diminishing returns.

$$WouldBeAccelerationIRI \equiv \frac{Commons * UnitAccelerationIRI}{MaximalAccelerationIRI} \tag{29}$$

The would-be acceleration of each individual item in the Commons is set in the user interface, as shown in the upper slider of Screenshot 25, above. The upper slider in Screenshot 25 has qualititative labels on the positions: none, a little, some, and a lot. Table 6 details the numerical values that correspond to each of the qualitative labels. Positions on the slider between the labels determine intermediate values for the would-be acceleration. For example, a position on the slide midway between None and A little determines a value of 0.001.

Label	UnitAccelerationIRI
None	0.0
A little	0.002
Some	0.005
A lot	0.01

Table 6: translating qualitative labels to values of UnitAccelerationIRI

As described above in Equation 23, individual response information both increases each week and declines each week. The decline occurs because the individual response information becomes less current as time passes and the health condition of the patient changes. The decay of individual response information is the amount of decline each week, and is determined by the half-life of individual response information, as shown in Equation 30. The half-life of individual response information is assumed to be twice the encounter period, twice the number of weeks between clinical encounters, as shown in Equation 31. So individual response information decays each week at the rate that its value would decline by 50% after two clinical encounters, if there was no increase from the processes described above.

$$IndividualResponseInformationDecay \equiv 1 - 0.5^{\frac{1}{IRIhalfLife}} \tag{30}$$

$$IRIhalfLife \equiv 2 * EncounterPeriod \tag{31}$$

The encounter period is set directly in the user interface, as shown in Screenshot 26.

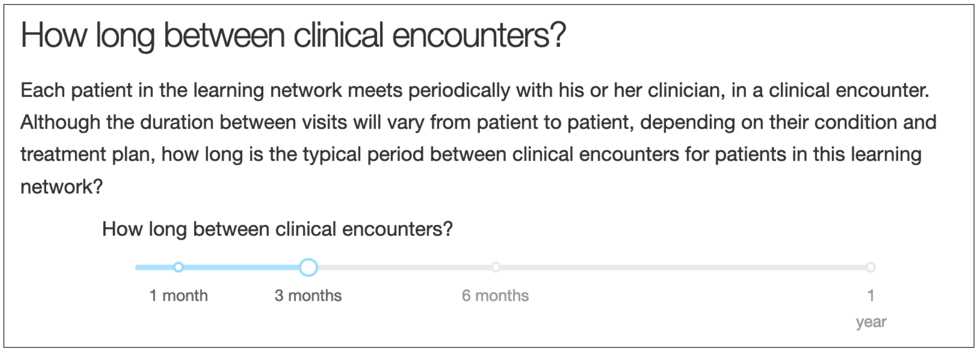

Screenshot 26: encounter frequency

Patient Commons Contribution sub-submodel

Sometimes an engaged patient will contribute something to the Commons. Most weeks, a particular patient will contribute nothing, but in some weeks that patient may contribute a single item. Equation 32 shows the model for patient contribution to the Commons.

$$PatientCommonsContribution \sim Bernoulli\left(\frac{1}{PatientContributionPeriod}\right) \tag{32}$$

The patient contribution period is the number of weeks on average between commons contributions, for a particular patient. That period depends on the engagement level of the patient, and is controlled in the user interface, as shown in Screenshot 27. Note that the patient engagement level can change from week to week, and so the contribution period can change. A patient with an engagement level of unaware or aware has an infinite contribution period, i.e. never contributes to the Commons.

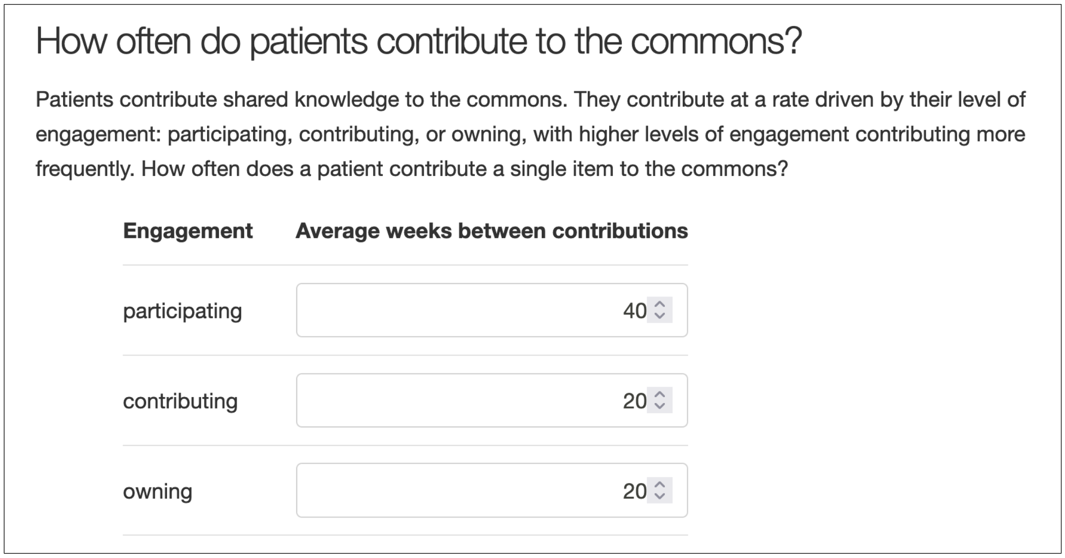

Screenshot 27: patient contribution frequency

Clinical Encounter sub-submodel

Every patient in the model has a clinical encounter with his clinician every so often. The duration between clinical encounters is set in the user interface, as shown in Screenshot 26. This duration is the same for all patients in the model.

When the **Clinical Encounter** sub-submodel executes for a particular patient, it determines whether the clinical encounter duration has passed. If it has not passed, nothing happens. If the clinical encounter duration has passed, the patient has a clinical encounter with his clinician.

In the world we are modeling, a lot happens during a clinical encounter. Not surprisingly, a lot happens within the **Clinical Encounter** sub-submodel: the phenotype response info is evaluated, the patient's individual response info is evaluated, praxis is updated, the patient's health status is evaluated, and a change in treatment regimen may be applied.

Phenotype response information

Phenotype response information is a measure for each patient of how much is known about treatment of the medical condition for patients with the same phenotype as the original patient. It is measured on a zero-to-one scale: zero means nothing is known, and one means that as much is known as can be.

Phenotype response information is determined by the product of potential phenotype response information and the proportion of potential realized, as shown in Equation 33.

$$PhenotypeResponseInfo \equiv PotentialPhenotypeResponseInfo * PRIproportionRealized \quad (33)$$

The amount of knowledge in the commons determines the potential phenotype response information, what the phenotype response information could be, with sufficient patient engagement and sufficient clinician engagement. The relationship is shown in Equations 34 and 35.

$$PotentialPhenotypeResponseInfo \equiv \frac{2}{1 + e^{-2* \, UnboundedPRI}} - 1 \quad (34)$$

$$UnboundedPRI \equiv Commons * PhenotypeResponseInfoPerCommonsItem$$

Each item in the commons has a small incremental contribution to phenotype response information, a contribution named in Equation 35 as phenotype response information per commons item. The per-commons item value is set by the user in the user interface, as shown in Screenshot 28.

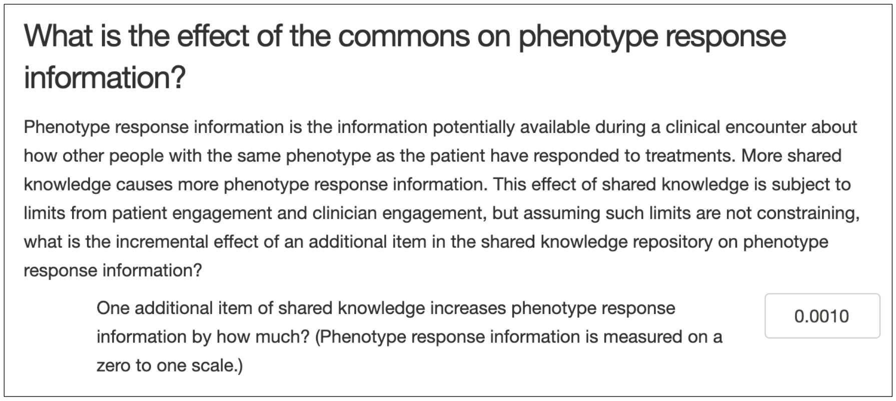

Screenshot 28: commons effect on phenotype response information contribution

Phenotype response information is bounded: it's value cannot be greater than one. If there are many items in the commons, the unbounded PRI in Equation 35 may be greater than one, perhaps much greater. But in practice, there are decreasing returns from additional items in the commons. The thousandth item in the commons will not have the same incremental effect as the first. Equation 34 converts the unbounded PRI to a bounded potential phenotype response information, as shown by the graph in Figure 2.

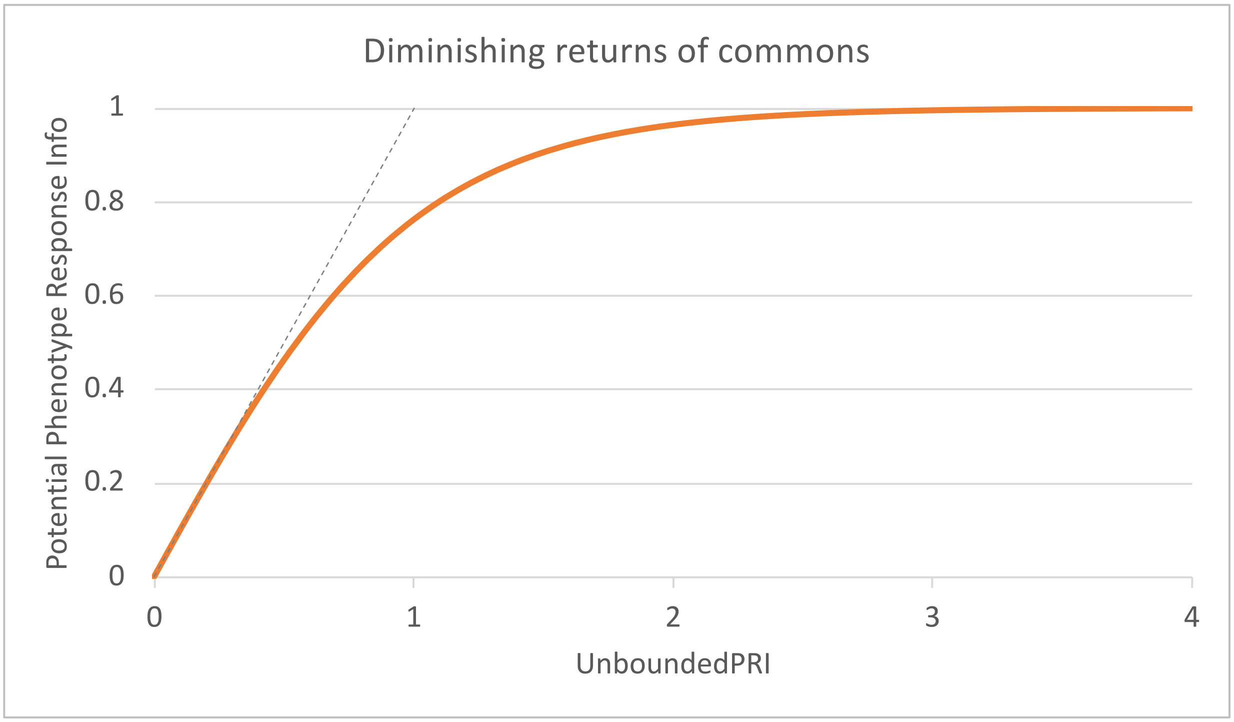

Figure 2: translating unbounded PRI to potential phenotype response info

The proportion of PRI realized—used in Equation 33 above—depends on the engagement of the patient and the patient's clinician, and the possibility of substituting records in the enhanced registry for the engagement. Proportion of PRI realized is measured on a zero-to-one scale. Equation 36 shows the details.

$$PRIproportionRealized \equiv RealizationFromEngagement + (1 - RealizationFromEngagement) * PotentialEngagementSubstitution \tag{36}$$

Realization from engagement is a weighted average of the patient engagement and the clinician engagement, as shown in Equation 37.

$$RealizationFromEngagement \equiv PatientEngagementValue * PhenotypeEngagementWeight + ClinicianEngagementValue * (1 - PhenotypeEngagementWeight) \tag{37}$$

Patient engagement value is measured on a zero-to-one scale, and determined by the patient engagement, as shown in Table 7. Since the patient engagement can change from week to week, patient engagement value also changes from week to week. Similarly, clinician value is also measured on a zero-to-one scale, and determined by the clinician engagement, via the same Table 7.

**Engagement**	**Engagement value**
Unaware	0.0
Aware	0.25
Participating	0.5
Contributing	0.75
Owning	1.0

Table 7: Patient engagement value and clinician engagement value

Phenotype engagement weight is measured on a zero-to-one scale, with zero meaning that only the engagement of the clinician affects potential phenotype realized, and one meaning that only the engagement of the patient affects potential phenotype realized. A value of 0.5 means that the engagement of the clinician and patient equally affect the potential realized. Phenotype engagement weight is set in the user interface, as shown in Screenshot 29.

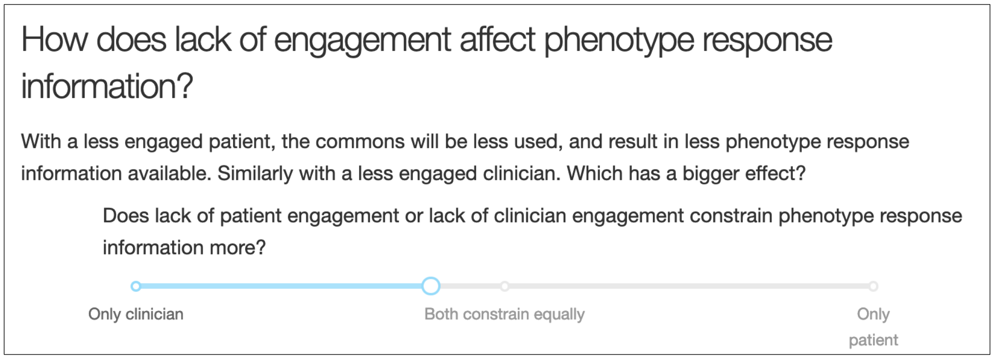

Screenshot 29: phentotype engagement weight

The enhanced registry can substitute for patient and clinician, as shown in Equation 36. This substitution occurs conditionally, only if the patient's care center both contributes to the enhanced registry and has the ability to use it in real-time. Equation 38 shows that conditionality.

$$PotentialEngagementSubstitution \equiv \begin{cases} EngagementSubstitution \\ if\ ERusage = contribution\ and\ realtime\ usage, \\ 0 \\ if\ ERusage = contribution, \\ 0 \\ if\ ERusage = no\ contribution \end{cases} \tag{38}$$

Engagement substitution is measured on a zero-to-one scale, with zero as no substitution when the enhanced registry is empty, and one as the (unattainable) perfect substitution of a great number of enhanced registry records. Equation 39 shows how engagement substitution is defined.

$$EngagementSubstitution \equiv \frac{2}{1 + e^{-2*EHRrecords*EngSubstPerEHRrecord}} - 1 \tag{39}$$

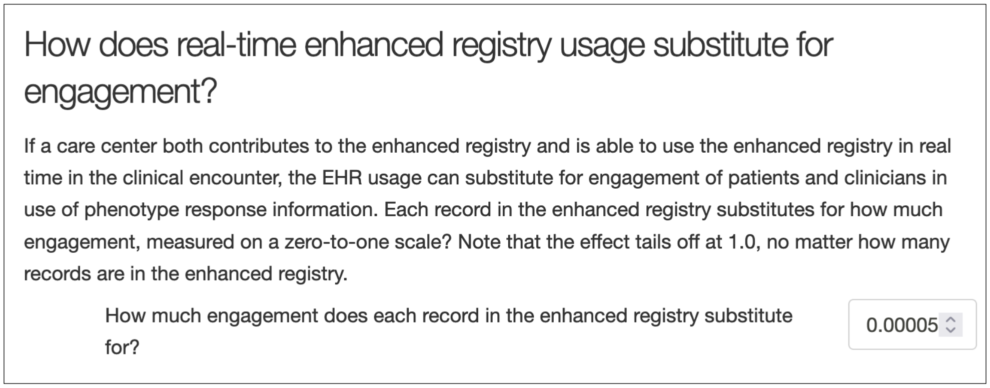

Screenshot 30: substituting enhanced registry records for engagement

The substitution depends on both the number of enhanced registry records and the amount of engagement substitution for each individual enhanced registry record. The value for engagement substitution per EHR record is set in the user interface, as shown in Screenshot 30. The number of enhanced registry records is a simple count of clinical encounters. If the clinical encounter takes place at a care center that contributes to an enhanced registry, the model increments the count.

Praxis

Praxis is updated during the **Clinical Encounter** sub-submodel. Praxis is a measure of how much is known about the condition of the patient for the purpose of making a treatment decision. Praxis varies from from one clinical encounter to the next.

In principle, praxis is a property of the patient-clinician dyad; the same patient working with one clinician would have a different praxis when working with another clinician. But in practice, for this model, each patient is only treated by a single clinician, and his clinician does not change over the course of the simulation. Since the clinician for a patient does not change, we model the praxis as a property of the patient rather than the patient-clinician dyad.

Praxis is measured on a zero to one scale, with zero as the praxis of an inexperienced solo practitioner treating a new patient, and one as the praxis of an experienced and knowledgeable clinician treating a long-term patient within a functional learning network.

Praxis is modeled as the product of the effective phenotype response information and the effective individual response information, as shown in Equation 40.

$$Praxis \equiv EffectivePhenotypeResponseInfo * EffectiveIndividualResponseInfo \tag{40}$$

Effective phenotype response information is the degree to which the patient and clinician bring information about the patient's phenotype to the clinical encounter. Effective individual response information is the degree to which the patient and clinician bring information about the patient response to treatment to the clinical encounter. Both are measured are a zero-to-one scale, where zero is bringing no such information and one is bringing a wealth of information, as much as can be brought.

Effective phenotype response information is modeled as the amount of response information available, potentially constrained by the degree of previsit planning practiced by the care center, as shown in Equation 41. Similarly, the effective individual response information is modeled as the amount of individual response information available, potentially also constrained by the degree of previsit planning practiced by the care center, as shown in Equation 42.

$$EffectivePhenotypeResponseInfo \equiv \min\left(PhenotypeResponseInfo, IndicatedPrevisitPlanningResponseInfo\right) \tag{41}$$

$$EffectiveIndividualResponseInfo \equiv \min\left(IndividualResponseInfo, IndicatedPrevisitPlanningResponseInfo\right) \tag{42}$$

Phenotype response info is described on page 32; individual response info is described on page 26. Indicated previsit planning response information for a care center depends on whether the care center both contributes to an enhanced registry and has the ability to use enhanced registry records in the clinical encounter. Equation 43 shows the conditionality.

$$IndicatedPrevisitPlanningResponseInfo \equiv \begin{cases} AugmentedPrevisitPlanningResponseInfo \\ \quad if\ ERusage = \text{contribution and realtime usage}, \\ PrevisitPlanningResponseInfo \\ \quad if\ ERusage = contribution, \\ PrevisitPlanningResponseInfo \\ \quad if\ ERusage = no\ contribution \end{cases} \tag{43}$$

Enhanced registry usage of the patient's care center can take one of three values: no contribution, contribution, or contribution and real-time usage. The value for enhanced registry usage of a care center is set in initialization, as described in Section 5. If the enhanced registry usage is either no contribution or contribution, indicated previsit planning response information takes the value of previsit planning response information, as described below. If the enhanced registry usage is contribution and real-time usage, indicated previsit planning response information takes a greater value, one determined by both the previsit planning response information and the number of records in the enhanced registry, also described below.

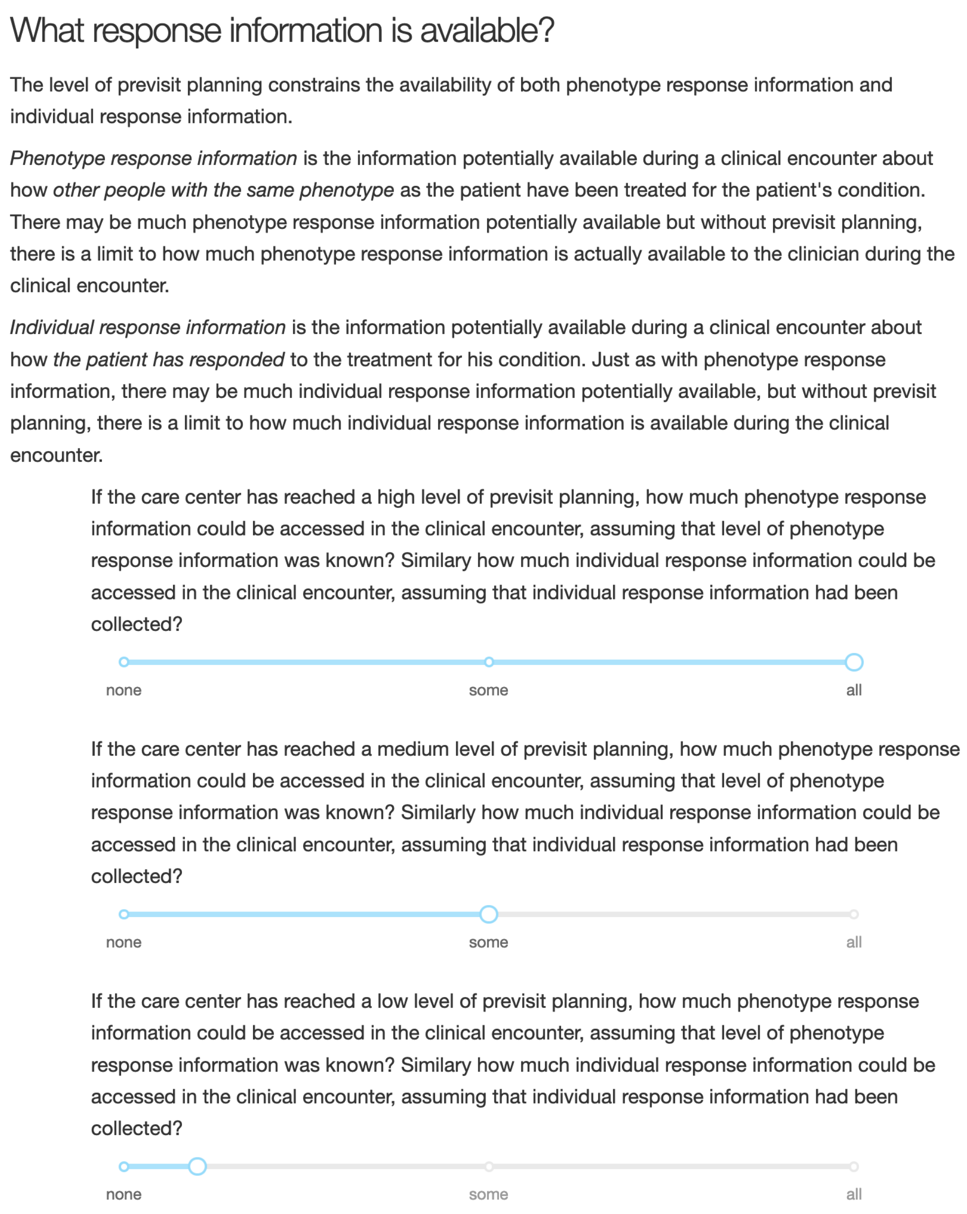

Screenshot 31: Previsit planning response info

Previsit planning response information acts as a constraint of the effectiveness of both phenotype response info and individual response info, as described above. This value for the constraint depends on the degree to which the care center practices previsit planning. The numerical value for the constraint is set in the user interface, as shown in Screenshot 31.

Augmented previsit planning response information allows records in the enhanced registry to substitute for a lack of previsit planning. Equation 44 shows the details of that substitution.

$$AugmentedPrevisitPlanningResponseInfo \equiv PrevisitPlanningResponseInfo + (1 - PrevisitPlanningResponseInfo) * EnhancedRegistrySubstitution \tag{44}$$

Enhanced registry substitution is the degree to which records in the enhanced registry substitute for lack of previsit planning. It is measured on a zero-to-one scale, with zero as no substitution when the enhanced

registry is empty, and one as the (unattainable) perfect substitution of a great number of enhanced registry records. Equation 45 shows how enhanced registry substitution is defined.

$$EnhancedRegistrySubstitution \equiv \frac{2}{1 + e^{-2*EHRrecords*SubstitutionPerEHRrecord}} - 1 \quad (45)$$

The substitution depends on both the number of enhanced registry records and the amount substitution for each individual enhanced registry record. The value for substitution per EHR record is a constant 0.00005.

Equation 45 ensures that the substitution per records has decreasing returns, so the effect of the first enhanced registry record has more impact than the thousandth record. Figure 3 shows the decreasing returns effect, for three values of substitution per EHR record.

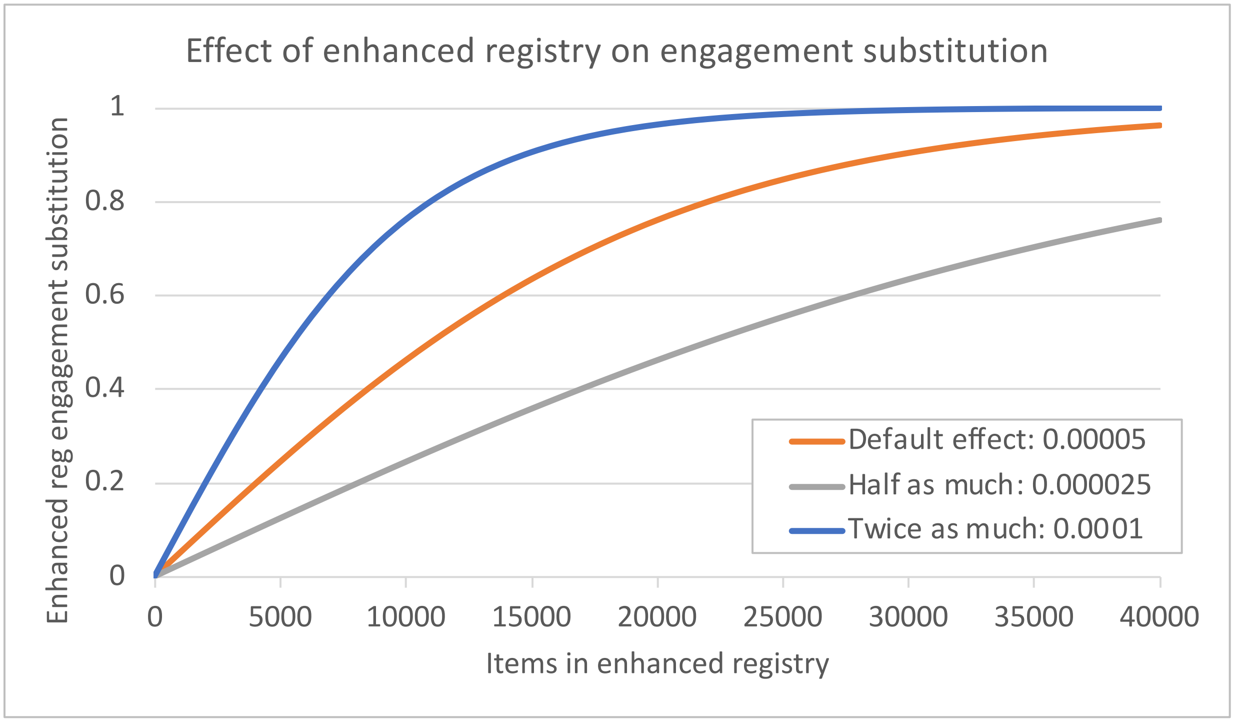

Figure 3: translating enhanced registry records to engagement substitution

Patient health status

During a clinical encounter—and within the **Clinical Encounter** sub-submodel—the clinician and patient evaluate the patient's health status, and whether it has improved since the prior clinical encounter. But the patient's health status is not entirely visible. The patient health status is perceived with some inaccuracy: the patient and clinician may perceive the patient's health status to be better than true health status, or they may perceive it to be worse than the true health status.

Equation 46 defines the perceived health status of the patient in a particular week. The perceived health status approximates the true health status, with some inaccuracy. In any given clinical encounter, evaluation inaccuracy acts as a standard deviation on draws on a truncated normal distribution. For example, if the true health status at a given week is 0.5, and the evaluation inaccuracy is 0.05, the patient's health status might be perceived as 0.45 or 0.55 but is unlikely to be perceived as 0.4—two standard deviations away from the ground truth—and is very unlikely to be perceived as 0.35, three standard deviations away.

$$PerceivedHealthStatus \sim TruncNormal(HealthStatus, EvaluationInaccuracy, 0,1)$$

Evaluation inaccuracy is driven by praxis, with greater praxis causing a smaller value for evaluation inaccuracy, making perceived health status more accurate. Less praxis causes a larger value for evaluation inaccuracy, making the perceived health status less accurate. Equation 47 defines the relationship between evaluation inaccuracy and praxis.

$$EvaluationInaccuracy \equiv EvaluationInaccuracyMinPraxis + Praxis * (EvaluationInaccuracyMaxPraxis - EvaluationInaccuracyMinPraxis) \tag{47}$$

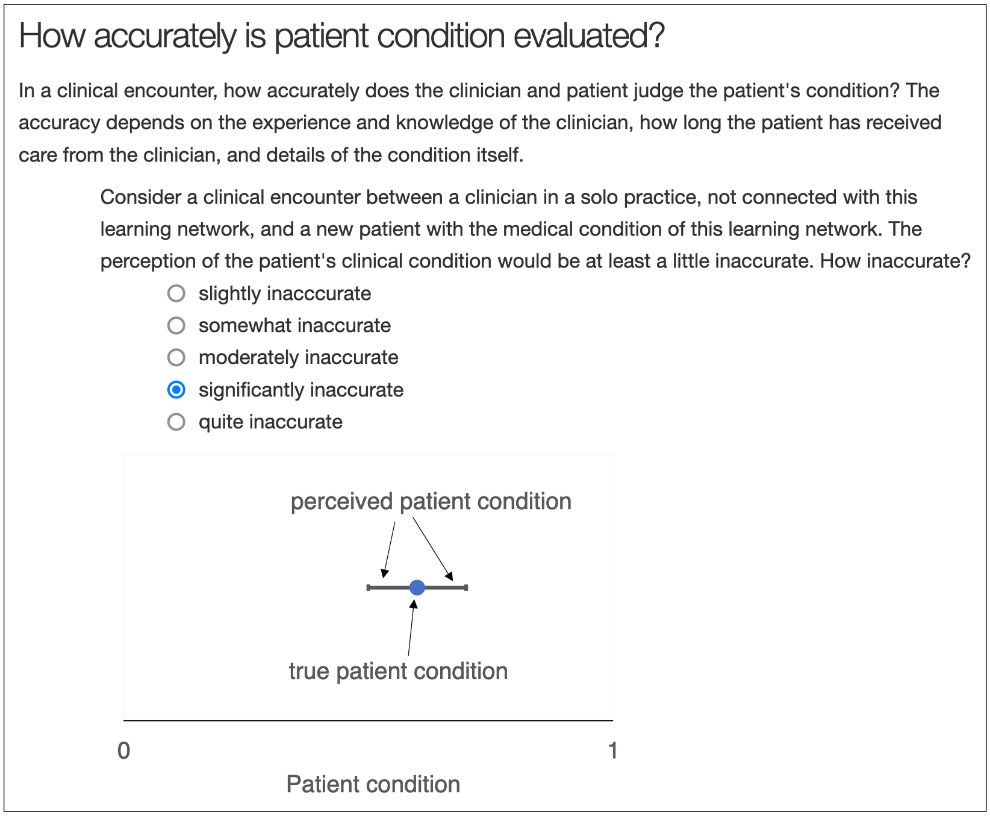

Screenshot 32: evaluation inaccuracy from minimum praxis

The evaluation inaccuracy for minimum praxis is specified for the medical condition by the user, in the user interface, as shown in Screenshot 32.

The five radio buttons in Screenshot 32 correspond to the values for evaluation inaccuracy shown in Table 8.

Radio button	Evaluation inaccuracy
Slightly inaccurate	0.01
Somewhat inaccurate	0.02
Moderately inaccurate	0.05
Significantly inaccurate	0.1
Quite inaccurate	0.2

Table 8: possible values of evaluation inaccuracy

The evaluation inaccuracy of maximum praxis for the medical condition is specified by the user in the same way, as shown in Screenshot 33. In addition to translating a radio button selection in Screenshot 32 to a value of *EvaluationInaccuracyMinPraxis*, the same Table 8 also translates a radio button selection in Screenshot 33 to a value of *EvaluationInaccuracyMaxPraxis*.

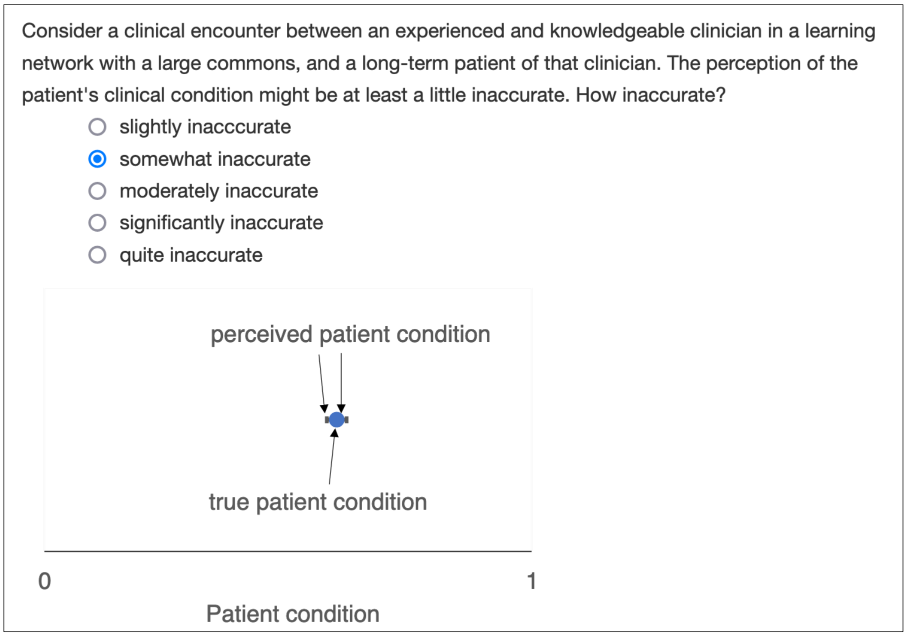

Screenshot 33: evaluation accuracy from maximum praxis

Treatment regimen

Once the patient's health status is evaluated, a change in the treatment regimen may be determined. If the patient's perceived health status has either improved since the prior clinical encounter—or is unchanged but is greater than 0.95—no change is made. If the patient's perceived health status has declined, the treatment regimen is changed. Of course, the patient's health status may be actually worsening but appear to have improved because of the inaccuracy of the perception of patient health status, described above. Similarly, the patient health status may be actually improving but appear to have worsened because of this inaccuracy.

If a new treatment regimen is applied, the new treatment regimen is selected from the set of possible treatment regimens, excluding the current treatment regimen from the set. In effect the clinician and patient say "That treatment regimen did not work. What should we try instead?"

$$TreatmentRegimen \sim Multinomial(TreatmentRegimenCount, TreatmentProbability_h) \tag{48}$$

As shown in Equation 48, the selection of the new treatment regimen is a draw on a multinomial distribution, with a probability for each alternative treatment regimen.   That probability is zero when *h* is the index of the current treatment regimen: the treatment regimen is to be changed, and there is no chance of not changing.

Equation 49 shows how the treatment probability of a treatment regimen is derived from the attractiveness of the treatment regimen, compared to the attractiveness of the alternative treatment regimens. When all treatment regimens are equally attractive, selection is random. Note that the attractiveness of the existing treatment regimen is zero.

$$TreatmentProbability_h \equiv \frac{Attractiveness_h}{\sum_h Attractiveness_h} \quad 49$$

Attractiveness is not a first-class variable of the simulation, some measure of attractiveness on a scale that could be explained and defended. Instead, attractiveness is a variable of convenience in this description. Without attractiveness, treatment probability would be expressed as single, more complex equation, instead of the simple Equations 49 and 50.

Equation 50 shows how the attractiveness of a treatment regimen depends on both its effectiveness and the selection efficiency. The effectiveness of a treatment regimen depends on the patient's phenotype, as described on page 21. The same treatmen regimen will have different degrees of effectiveness for patients of different phonotypes: e.g. effective for one patient, ineffective for another patient, and counterproductive for a third patient. Of course, a patient's phenotype is not known, and even if it were known, the effectiveness of a particular treatment regimen for that phenotype may be uncertain, given the knowledge of the medical condition.

$$Attractiveness_h \equiv e^{Effectiveness_h * SelectionEfficiency} \quad 50$$

The effectiveness of a treatment regimen is modeled as the change in health status of the patient per week if that treatment regimen were applied to that patient. For example, a gradually effective treatment regimen might have an effectiveness of 0.02—for that patient, and other patients of the same phenotype—indicating an increase in patient health condition of 0.02 per week. An ineffective treatment regimen might have an effectiveness of 0.0, and a counterproductive treatment regimen might have an effectiveness of -0.02.

Selection efficieny is a measure of how well the patient-clinician dyad is able to choose a more effective treatment regimen rather than a less effective treatment regimen. Selection efficiency is modeled as a non-negative real number. If the selection efficiency of the clinician and patient is zero, all three treatment regimens are equally attractive, and equally likely to be chosen. If the selection efficiency of the clinician and the patient is 1000, the best treatment regimen will be far more attractive, and will almost always be applied.

Consider a situation with three treatment regimens: A, B, and C. A has an effectiveness of 0.02 (for a particular phenotype), B has an effectiveness of 0.0, and C has an effectiveness of -0.02. Table 9 shows the resultant attractiveness at different degrees of selection efficiency, according to Equation 50.

Selection efficiency	TR A	TR B	TR C
0	1	1	1
1	1.02	1	0.98
10	1.22	1	0.82
100	7.38	1	0.14
1000	485 million	1	Close to zero

Table 9: example attractiveness, depending on selection efficiency

Continuing the example, Table 10 shows the resulting treatment probabilities of the three alternative treatment regimens, applying Equation 49. With a selection efficiency of zero, the patient-clinician dyad is entirely guessing. With a selection efficiency of 10, the dyad is somewhat effective at making the better choice. With a selection efficiency of 100, they are quite effective at making the better choice.

Selection efficiency	TR A	TR B	TR C
0	33%	33%	33%
1	34%	33%	32%
10	40%	33%	27%
100	87%	12%	2%
1000	100%	0%	0%

Table 10: treatment probabilities, depending on selection efficiency

Selection efficiency is determined by both praxis and by the minimum and maximum selection efficiency, as shown in Equation 51. Praxis scales selection efficiency from the minimum selection efficiency—when praxis is zero—to the maximum selection efficiency—when praxis is one.

$$SelectionEfficiency \equiv SelectionEfficiencyMin + (SelectionEfficiencyMax - SelectionEfficiencyMin) * Praxis \quad (51)$$

The minimum and maximum selection efficiency is determined by the knowledge of the medical condition, described on page 18. For medical conditions about which much is known (e.g. pneumonia), the minimum selection efficiency is relatively large. Even an inexperienced solo practitioner working with a new patient—praxis of zero—will do fairly well at selecting a treatment regimen. The maximal selection efficiency—praxis of one—is even larger. For medical conditions about which little is known (e.g. an ultra-rare disease), the minimum selection efficiency is relatively small. An inexperienced solo practitioner working with a new patient—praxis of zero—will have difficulty selecting an effective treatment regimen. An experienced and knowledgeable clinician treating a long-term patient—praxis of one—will fare better, but still be challenged.

Table 11 shows the numeric values for minimum and maximum selection efficiency for three different values of medical condition knowledge. These values were estimated from expert interview, an interview with a clinician, posing questions about likelihood of recognizing the best treatment regimen under different circumstances.

Medical condition knowledge	Example	Minimum selection efficiency	Maximum selection efficiency
0.0	Ultra-rare disease	0	12
0.5	Depression and anxiety	25	50
1.0	Pneumonia	70	180

Table 11: min and max selection efficiencies for different knowledge of medical condition

What about medical conditions for which the knowledge is not 0.0, 0.5, or 1.0? For those medical conditions, the minimum selection efficiency is given by Equations 52 and 53.

$$SelectionEfficiencyMin \equiv MedicalConditionKnowledge^{1.485} * 70 \quad (52)$$

$$SelectionEfficiencyMax \equiv MedicalConditionKnowledge^{2.144} * 168 + 12 \quad (53)$$

Equations 52 and 53 create minimum and maximum selection efficiencies for any medical condition knowledge between 0 and 1, consistent with Table 11, as shown in Figure 4.

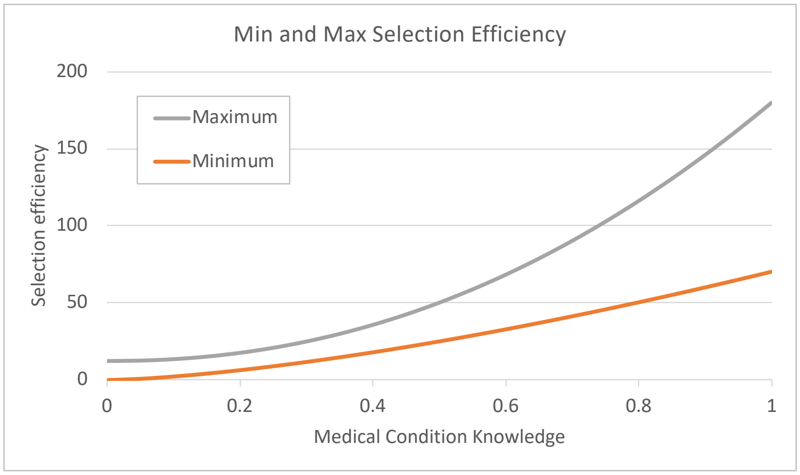

Figure 4: minimum and maximum selection efficiencies

Patient Transition sub-submodel

As described in on page 3, each patient is engaged with the learning network to some extent, an engagement modeled on a five-point ordinal scale: unaware, aware, participating, contributing, or owning. In the **Patient Transition** sub-submodel, a patient may transition from one engagement state to a different engagement state. There are three kinds of transitions:

1. become aware: a transition from unaware to aware
2. activate: a transition from aware to participating, from participating to contributing, or from contributing to owning
3. dispirit: a transition from owning to contributing, from contributing to participating, or from participating to aware

For a particular patient, the three kinds of transitions are mutually exclusive: a patient who becomes aware in one week will not also activate or dispirit. A patient who activates in one week will not also dispirit.

A patient who is unaware in a particular week can become aware, in one of two ways: either as a result of a clinical encounter, or as a result of influence by another patient. If an unaware patient has a clinical encounter with a clinician who is not unaware (i.e. who is aware, participating, contributing, or owning), the patient might become aware of the learning network, with some probability, as shown in Equation 54.

$$AwareViaClinicalEncounter \sim Bernoulli(EncounterAwareLikelihood) \quad (54)$$

The probability that a clinical encounter will cause patient awareness is controlled in the user interface, as shown in Screenshot 34.

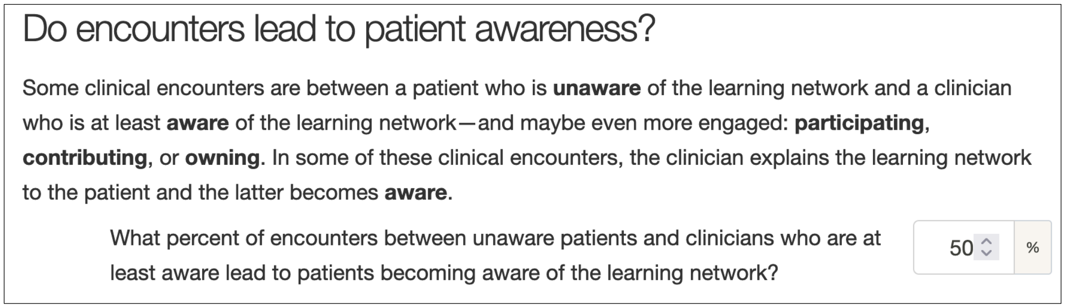

Screenshot 34: encounter aware likelihood

An unaware patient may transition to aware as a result of influence by another patient. Each patient has influence links with some other patients in the network, as described on page 11. One or more of these other patients may be at least aware of the learning network, i.e. have an engagement of aware, participating, creating or owning. The unaware patient then may learn about the learning network via conversation with his not-unaware friend.

We model the transition to aware as a result of influence by another patient as a draw on a Bernoulli distribution, as shown in Equation 55.

$$AwareViaPatientInfluence \sim Bernoulli(PatientInfluenceAwareProb) \tag{55}$$

The draw in Equation 55 occurs weekly for each influence link between an unaware patient and a not-unaware patient to which the unaware patient has an influence link. If patient Zack has influence links to 10 other patients, each of which is not unaware, there will be 10 draws every week for Zack, ten chances for him to become aware of the learning network, and transition from an engagement level of unaware to an engagement level of aware.

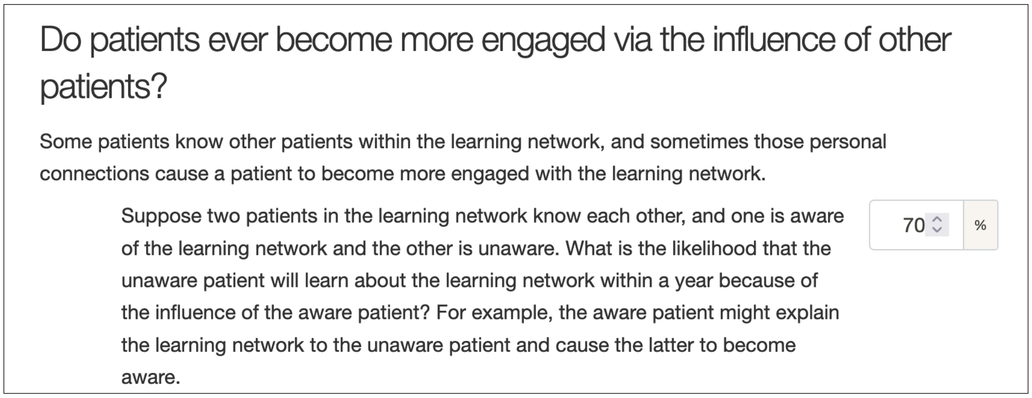

Screenshot 35: probability of a patient becoming aware due to an influence link

The probability *PatientInfluenceAwareProb* is controlled with a dialog in the user interface, as shown in Screenshot 35. The probability in Equation 55 is a weekly probability. The probability controlled in Screenshot 35 is an annual probability**.** The annual is translated to weekly, as shown in Equation 56. For example, the annual probability of 70% shown in Screenshot 35 translates to a weekly probability of roughly 2.29%.

$$PatientInfluenceAwareProb \equiv 1 - \sqrt[52]{1 - PatientInfluenceAwareAnnualProb} \tag{56}$$

A patient who is not unaware can activate—transition from one engagement level to a higher engagement level—i.e. from from aware to participating, from participating to contributing, or from contributing to owning. As with becoming aware, activation can occur either because of the influence of the clinician during a clinical encounter, or because of the influence of another patient, some other patient connected to the original patient by an influence link.

During a clinical encounter between a patient who is less engaged with the learning network and a clinician who is more engaged, there is some chance of the patient activating. The activation is modeled as a draw on a Bernoulli distribution, shown in Equation 57.

$$ActivationViaClinicalEncounter \sim Bernoulli(PatientClinicalEncounterIActivationProb) \quad (57)$$

The probability of activation depends on the combination of the engagement of the patient and the engagement of the clinician. Screenshot 36 shows the possible combinations and the probabilities of each. These probabilities are set by the user in the user interface. Note that if the patient has already attained an engagement level of owning, he cannot further activate.

As with becoming aware, a patient can activate due to influence from other patients, those to whom he is connected by influence links. Some of those other patients may have greater levels of engagement than the patient. We model the activation due to influence links as another draw on a Bernoulli distribution. Note that this draw occurs repeatedly each week, once for each patient via influence link connection who has a greater level of engagement that our patient.

Might a clinical encounter increase patient engagement?

A clinical encounter between a patient who is at least **aware** of the learning network and a clinician who is also at least **aware** might lead to a greater level of patient engagement. What is the probability (as a percentage) of an increased level of patient engagement, based on the existing engagement for both the patient and the clinician? Note that a patient who is already at an engagement of **owning** cannot increase engagement any further.

**How likely?**		**Patient engagement:** from aware to participating	from participating to contributing	from contributing to owning	owning
**Clinician engagement**	**aware**	0 %	0 %	0 %	0%
	**participating**	5 %	0 %	0 %	0%
	**contributing**	10 %	5 %	0 %	0%
	**owning**	20 %	10 %	5 %	0%

Screenshot 36: probability of a patient becoming more engaged due to clinical encounter

The probability of activation is controlled with a dialog in the user interface, as shown in Screenshot 37. The probability in Equation 58 is a weekly probability while the probability controlled in Screenshot 37 is an annual probability. The annual is translated to the weekly, as shown in Equation 59.

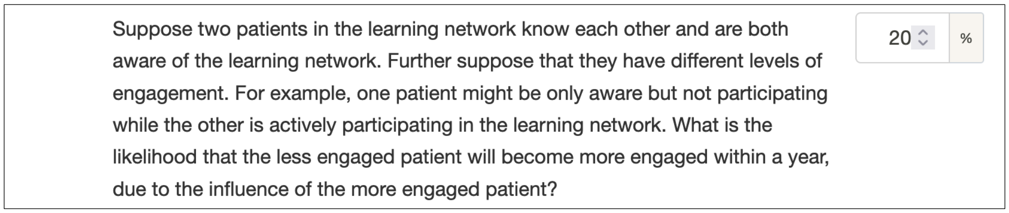

Screenshot 37: probability of a patient becoming more engaged due to an influence link

$$PatientActivationViaInfluence \sim Bernoulli(PatientInfluenceActivationProb) \quad (58)$$

$$PatientInfluenceActivationProb \equiv 1 - \sqrt[52]{1 - PatientInfluenceActivationAnnualProb} \quad (59)$$

A patient who is not unaware can dispirit—transition from a higher level of engagement to a lower level of engagement—i.e. from owning to contributing, from contributing to participating, or from participating to aware. (A patient who is aware cannot become unaware.) Unlike becoming aware or activation, dispiriting can only occur because of the influence of the clinician during a clinical encounter.

A clinical encounter between a patient who is more engaged and a clinician who is less engaged can cause patient dispiriting. Equation 60 shows the decline in engagement as a draw on a Bernoulli distribution.

$$DispiritViaClinicalEncounter \sim Bernoulli(PatientClinicalEncounterDispiritProb) \quad (60)$$

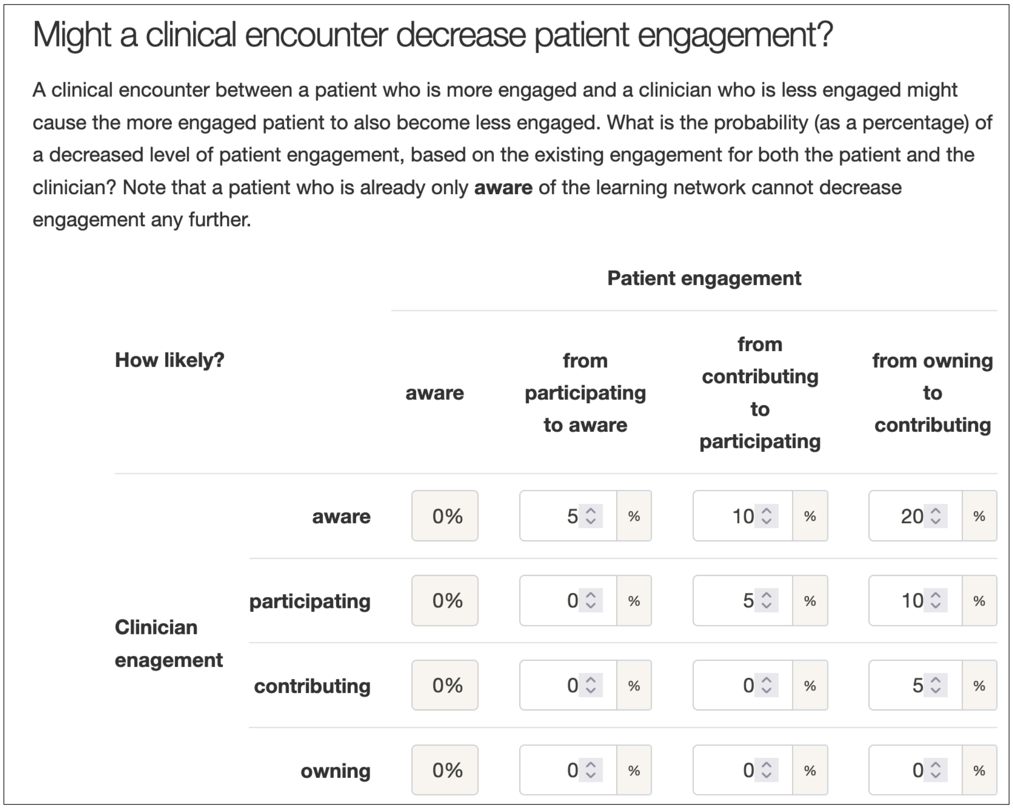

Might a clinical encounter decrease patient engagement?

A clinical encounter between a patient who is more engaged and a clinician who is less engaged might cause the more engaged patient to also become less engaged. What is the probability (as a percentage) of a decreased level of patient engagement, based on the existing engagement for both the patient and the clinician? Note that a patient who is already only **aware** of the learning network cannot decrease engagement any further.

How likely?		Patient engagement: aware	from participating to aware	from contributing to participating	from owning to contributing
Clinician enagement	aware	0%	5 %	10 %	20 %
	participating	0%	0 %	5 %	10 %
	contributing	0%	0 %	0 %	5 %
	owning	0%	0 %	0 %	0 %

Screenshot 38: probability of a patient becoming less engaged due to clinical encounter

As with activation, the probability of dispiriting depends on the combination of the engagement level of the patient and the engagement level of the clinician. Screenshot 38 shows the combinations and the probabilities of each. These probabilities can be set in the user interface. Note that if the patient has an engagement level of aware, he cannot further dispirit. A clinical encounter cannot erase the awareness of the learning network from his memory.

Patient Leave Cohort sub-submodel

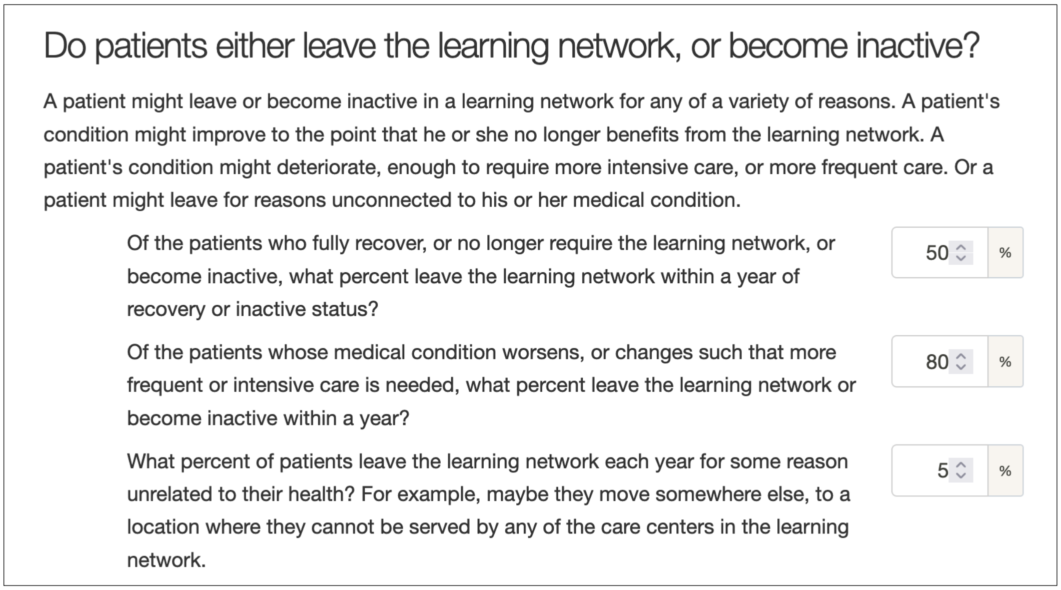

Screenshot 39: probability of a patient leaving the learning network

The final sub-submodel in the **Update Patient** submodel is **Patient Leave**. Sometimes patients leave a learning network. A patient's health status might improve to the point that he no longer benefits from the learning network. Then he might leave. A patient's health status might worsen to the point that he requires more intensive care or more frequent care, care beyond the capability of the learning network. Then he might leave.

Patients sometimes leave a learning network for exogenous reasons, reasons that have nothing to do with their health status. For example, a patient may physically move to a geography where no care centers are participating in the learning network.

Patient exit from the learning network is configured in the user interface, as shown in Screenshot 39. The rate of exit is specified in terms of the probability that a patient in the potentially exiting situation will leave within a year. For example, the default exit percentage for patients who fully recover is 50% in a year. Of course patients are simulated week by week: every week the model determines if this fully recovered patient leaves the learning network this week. That determination is a draw from a Bournouli distribution, as shown in Equation 61.

$$RecoveredExit \sim Bernoulli(RecoveredExitWeeklyProb) \qquad 61$$

The weekly probability of recovered exit is a function of the annual probability of recovered exit, as specified in the user interface of Screenshot 39. The function is defined in Equation 62. For example, if the annual probability is 50%, the weekly probability is roughly 1.3%.

$$RecoveredExitWeeklyProb \equiv 1 - \sqrt[52]{1 - RecoveredExitAnnualProb} \qquad 62$$

Similarly the weekly probability of a patient exiting the learning network when his medical condition sufficiently worsens in defined in Equations 63 and 64. The weekly probability of a patient exiting the learning network for other reasons is defined in Equations 65 and 66.

$$WorsenExit \sim Bernoulli(WorsenExitWeeklyProb) \tag{63}$$

$$WorsenExitWeeklyProb \equiv 1 - \sqrt[52]{1 - WorsenExitAnnualProb} \tag{64}$$

$$ExogenousExit \sim Bernoulli(ExogenousExitWeeklyProb) \tag{65}$$

$$ExogenousExitWeeklyProb \equiv 1 - \sqrt[52]{1 - ExogenousExitAnnualProb} \tag{66}$$

Screenshot 39 allows the user to change the probability that a patient will leave the learning network when he fully recovers. But what counts as full recovery? By default, when a patient’s health status is at 0.9 or greater, he is considered to have fully recovered. But that threshold of full recovery can be changed by the user to a different value, some number either greater than 0.9 or less than 0.9. Screenshot 40 shows how the user can set the full recovery threshold.

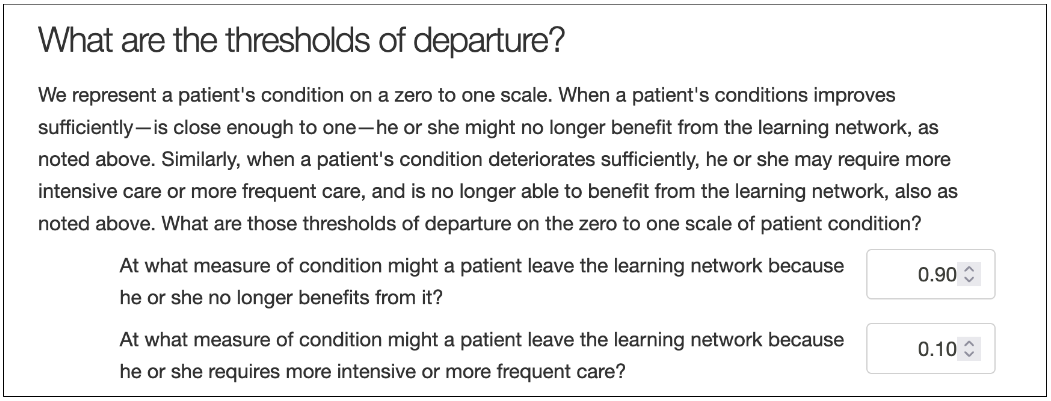

Screenshot 40: thresholds of departure

In the same way, by default when a patient’s health status is at 0.1 or less, he is considered to require more intensive care or more frequent care, and exits the learning network to receive that care. That 0.1 threshold can also be changed by the user, as shown in Screenshot 40.

Update Clinician submodel

The **Update Clinician** submodel consists of two sub-submodels, executed in order with each execution of the **Update Clinician** submodel for a particular clinician agent:

1. Clinician Transition sub-submodel
2. Clinician Commons Contribution sub-submodel

Both of these sub-submodels are executed every week, but make a difference only occasionally. For example, the **Clinician Transition** sub-submodel is executed every week for each clinician in the simulation, to see if the clinician will transition from one engagement level to another. But a particular clinician will only change engagement levels occasionally, not every week.

Clinician Transition sub-submodel

The **Clinician Transition** sub-submodel is much like the **Patient Transition** sub-submodel, described on page 43. Each clinician is engaged with the learning network to some extent, an engagement modeled on a five-

point ordinal scale: unaware, aware, participating, contributing, or owning. A clinician may transition from one engagement state to a different engagement state. As with patient transition, there are three kinds of clinician transitions:

1. become aware: a transition from unaware to aware
2. activate: a transition from aware to participating, from participating to contributing, or from contributing to owning
3. dispirit: a transition from owning to contributing, from contributing to participating, or from participating to aware

For a particular clinician, the three kinds of transitions are mutually exclusive: a clinician who becomes aware in one week will not also activate or dispirit. A clinician who activates in one week will not also dispirit.

Each clinician has influence links with some other clinicians in the network, as described on page 16. We assume that the two clinicians on either side of an influence link have a continuing relationship through the extent of the simulation. An unaware clinician can become aware as a result of influence by another clinician. One or more of these other clinicians may be at least aware of the learning network, i.e. have an engagement of engagement of aware, participating, creating or owning. The unaware clinician then may learn about the learning network via conversation with her not-unaware colleague.

We model the transition to aware as a result of influence by another clinician as a draw on a Bernoulli distribution, as shown in Equation 67.

$$AwareViaCliniciannfluence \sim Bernoulli(ClinicianInfluenceAwareProb) \quad (67)$$

As with patients, the draw in Equation 67 occurs weekly for each influence link between an unware clinician and a not-unaware clinician to whom the unaware clinician has an influence link. If Dr. Megan is unaware, and has influence links to 7 other clinicians, each of which is not aware, there will be 7 draws every week for Dr. Megan, seven chances for her to become aware of the learning network, and transition from an engagement level of unaware to an engagement level of aware.

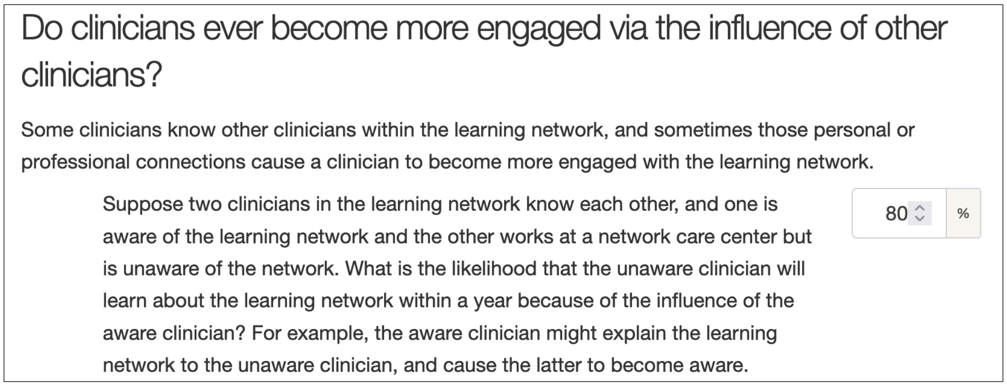

Screenshot 41: probability of a clinician becoming aware due to an influence link

The probability of clinician influence causing awareness in Equation 67 is controlled with a dialog in the user interface, as shown in Screenshot 41. The probability in Equation 67 is a weekly probability. The probability controlled in Screenshot 41 is an annual probability: *ClinicianInfluenceAwareAnnualProb*. The annual is translated to weekly, as shown in Equation 68.

$$ClinicianInfluenceAwareProb \equiv 1 - \sqrt[52]{1 - ClinicianInfluenceAwareAnnualProb} \tag{68}$$

A clinician who is not unaware can activate—transition from one engagement level to a higher level of engagement—i.e. from from aware to participating, from participating to contributing, or from contributing to owning. Activation occurs because of the influence of another clinician who has a higher level of engagement, some clinician connected to the less-engaged clinician by an influence link. The model mechanism for an increase in clinician engagement is yet another draw from a Bernoulli distribution, as shown in Equation 69. Note that this draw occurs repeatedly each week, once for each clinician linked by influence who has a greater level of engagement.

$$ClinicianActivationViaInfluence \sim Bernoulli(ClinicianInfluenceActivationProb) \tag{69}$$

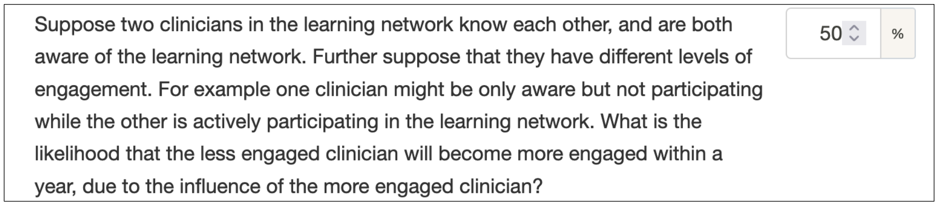

Screenshot 42: probability of a clinician increasing engagement via an influence link

The probability of a clinician activating *nicianInfluenceActivationProb* is controlled with a dialog in the user interface, as shown in Screenshot 42. The probability in Equation 69 is a weekly probability while the probability controlled in Screenshot 42 is an annual probability. The annual is translated to the weekly via Equation 70.

$$ClinicianInfluenceAwareProb \equiv 1 - \sqrt[52]{1 - ClinicianInfluenceAwareAnnualProb} \tag{70}$$

A clinician can dispirit—her engagement with the learning network can decline—when she becomes burned out, and reduces her effort. When a clinician burns out, her engagement with the learning network decreases by a single level, from owning to contributing, from contributing to participating, or from participation to merely aware. A clinician experiencing burnout will never become unaware of the learning network.

$$ClinicianBurnout \sim Bernoulli(ClinicianBurnoutProb) \tag{71}$$

Burnout is modeled as a weekly draw on another Bournoulli distribution, as shown in Equation 71.

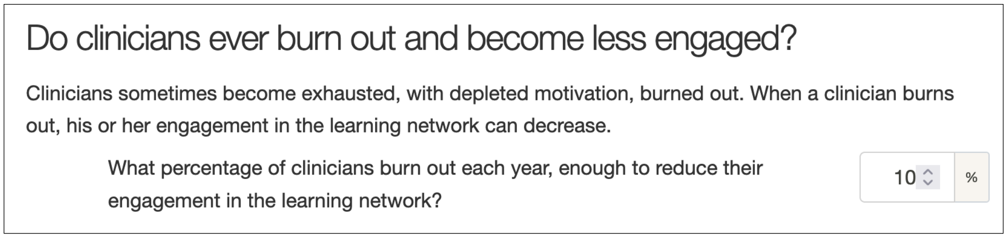

Screenshot 43: probability of clinician burnout

The probability of clinician burnout is controlled with a user interface dialog, as shown in Screenshot 43. As with the other user-controlled probabilities, the probability in Equation 71 is a weekly probability while that controlled in Screenshot 43 is an annual probability. The annual is translated to the weekly via Equation 72.

$$ClinicianBurnoutProb \equiv 1 - \sqrt[52]{1 - ClinicianBurnoutAnnualProb} \quad (72)$$

Clinician Commmons Contribution sub-submodel

The **Clinician Commons Contribution** sub-submodel is much like the **Patient Commons Contribution** sub-submodel, described on page 31. Sometimes an engaged clinician will contribute something to the Commons. Most weeks, a particular clinician will contribute nothing, but in some weeks, that clinician may contribute a single item. Equation 73 shows clinician commons contribution as a draw on a Bernoulli distribution.

$$ClinicianCommonsContribution \sim Bernoulli\left(\frac{1}{ClinicianContributionPeriod}\right) \quad (73)$$

The clinician contribution period is the number of weeks on average between commons contributions, for a particular clinician. That period depends on the engagement level of the patient, and is controlled by the user interface, as shown in Screenshot 44. Note that the engagement of a particular clinician can change from week to week, and so the contribution period can change. A clinician with an engagement level of unaware has an infinite contribution period, i.e. never contributions to the Commons.

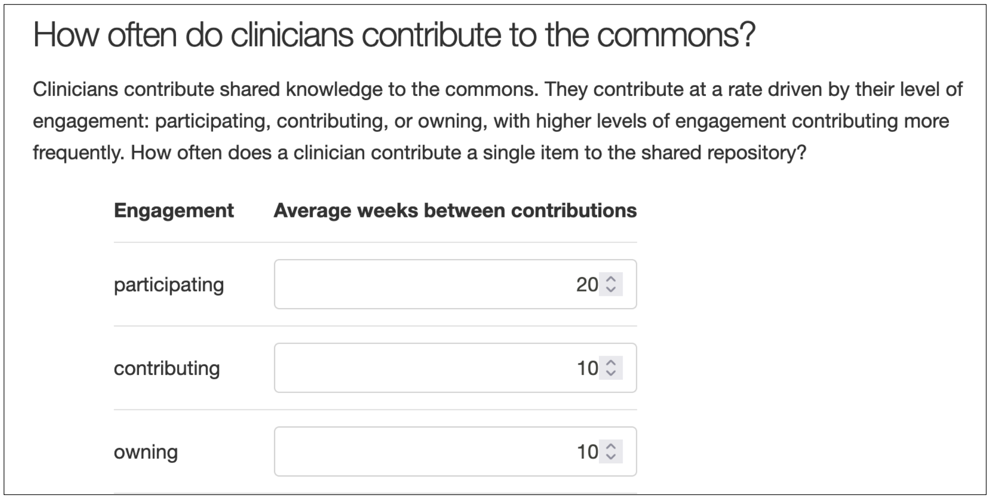

Screenshot 44: cliniciant commons contribution frequency

Enhanced Registry submodel

The **Enhanced Registry** submodel manages the contributions to the Commons that result from periodic analyst of the enhanced registry. Every so often the clinical encounter records in the enhanced registry are analyzed, resulting in a contribution of shared knowledge to the Commons.

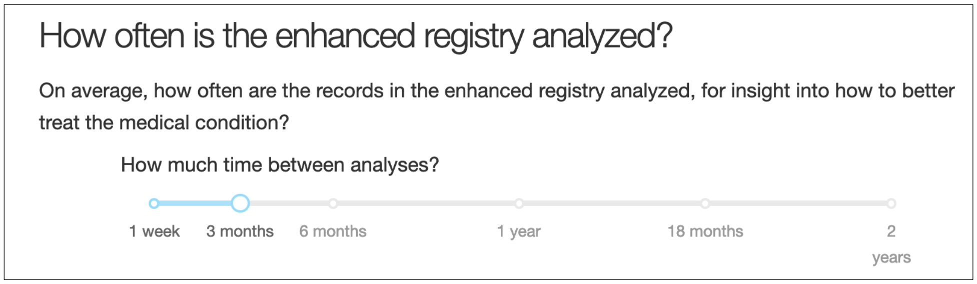

Screenshot 45: enhanced registry analysis frequency

The frequency of analysis is determined via the user interface, as show in Screenshot 45.

An analysis of records in the enhanced registry results in a contribution of shared knowledge to the Commons. The number of items contributed is determined by Equation 74. As described on page 35, the enhanced registry record count is the simple count of records in the enhanced registry.

$$EnhancedRegistryConstribution \equiv Floor(\frac{EnhancedRegistryRecordCount}{RecordsPerCommonsItem}) \tag{74}$$

Equation 74 translates the current number count of enhanced registry records to a commons contribution, using a constant of records per commons item. Records per commons item is set in the user interface, as shown in Screenshot 46.

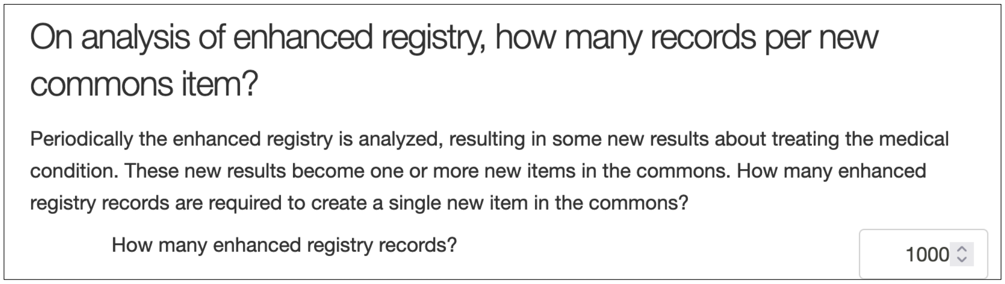

Screenshot 46: enhanced registry contribution amount

The floor function in Equation 74 takes a real number and provides the largest integer that is less than or equal to the real. For example, suppose there are 1,151 records in the enhanced registry and the rocrds per commons item is 1000. The analysis of the records in the enhanced registry results in a contribution of Floor(1.151) or 1 item. Note that if the number of records in the enhanced registry is less than the records per commons item, no items will be contributed.

Patient Arrive submodel

The **Patients Arrive** submodel adds patients to the learning network simulation. Every week patients are added for two reasons:

- A newly founded learning network begins with no patients. Patients are enrolled over the first several weeks of the simulation until all the patients have been enrolled.
- As patients depart the learning network, the resulting open spots allow new patients to be enrolled.

As described on page 11, the learning network simulation can be used to model either an existing learning network or a learning network that does not yet exist. In the latter case, patients are enrolled in the learning network gradually, over the initial weeks of the simulation. Each week, some quantity of patients are enrolled, as defined in Equation 75.

$$PatientEnrollmentQty \equiv Min(PatientsToBeEnrolled, Ceiling\left(\frac{TotalPlannedPatients}{EnrollmentDuration}\right)) \tag{75}$$

The min function provides the lesser of its two arguments, ending patient enrollment once all the patients have been enrolled. Equation 75 uses a count of patients that have not yet been enrolled. During the first weeks of the simulation, as patients are enrolled, this count is decremented. After all patients have been enrolled, the count is zero, and no more patients will be enrolled.

The ceiling function in Equation 75 takes a real number and provides the smallest integer that is greater than or equal to the real. It is the counterpart of the floor function used in Equation 74. If the learning network has 200 patients planned, and the enrollment duration is 10 (weeks), ceiling is applied to 20.0 resulting in 20 patients added. If the enrollment duration is 9, 200/9 is roughly 22.2, and 23 patients are enrolled each week.

Equation 75 uses the total number of patients planned for the learning network. That total number is determined during initialization, as the sum of patients for all care centers. Enrollment duration is set in the user interface, as shown in Screenshot 47.

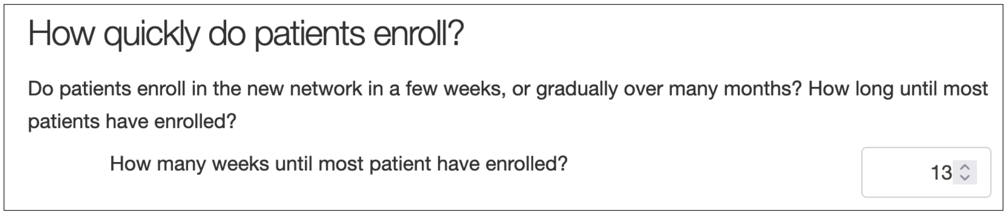

Screenshot 47: patient enrollment pace

In addition to enrolling patients gradually, over the initial weeks of the simulation, patients are also added to replace departing patients, to fill the open spots left behind. Some patients depart during the course of the simulation, as described on page 47. For every patient who leaves, a new one is added.

Initializing a patient after the simulation has started—within the **Patients Arrive** submodel, either from the gradual initial enrollment or replacing a departing patient—is the same process as initializing a patient at the beginning of the simulation, as described on page 11.

1. Fishbein M. A reasoned action approach to health promotion. *Med Decis Making*. Nov-Dec 2008;28(6):834-44. doi:10.1177/0272989x08326092
