# Supplementary material for: An agent-based model to advance the science of collaborative learning health systems
Source: PLoS One. 2025 Sep 9;20(9):e0332054. doi: 10.1371/journal.pone.0332054 (PMC12419628; doi:10.1371/journal.pone.0332054)
Supplement: S2 Supplement — (PDF) [file pone.0332054.s002.pdf]

Table S2: LHN ABM parameters varying in the sensitivity analysis.

| |
|---|
| 1. **encounter_period** (*int or None*) – after how many weeks does a patient have a clinical encounter with a clinician? Valid options: an integer: all patients have the same encounter period or None: each patient has a different encounter period, selected randomly, of at least 10 weeks, and no more than 16 weeks. Default: 13 |
| 2. **encounter_aware_likelihood** (*float*) – if a clinical encounter might lead to a patient becoming aware (i.e. **encounter_aware_on** is True), what is the probability of an unaware patient becoming aware, during a clinical encounter with a clinician who is at least aware? Default: 0.5 |
| 3. **patient_activate_determiner** (*TransitionDeterminer or dict[str, dict[str, float] ] or float*) – if a clinical encounter might cause a patient to increase activation (i.e. **encounter_activate_on** is True), what is the probability of an increased activation? Could be provided as either a TransitionDeterminer object, or as a nested dictionary, or as a float. If provided as a dictionary, the key of each element in the dictionary is a patient state. The value of an element is a dict of clinician states and probabilities of increased activation. If provided as a single float, the probability of activate for an owning clinician and an aware patient is provided, and others calculated from that value. Default: TransitionDeterminer.default_activate() |
| 4. **patient_dispirit_determiner** – if a clinical encounter might cause a patient to lower activation (i.e. **encounter_dispirit_on** is True), what is the probability of a lowered activation? Could be provided as either a TransitionDeterminer object, or as a nested dictionary, or as a float. If provided as a dictionary, the key of each element in the dictionary is a patient state. The value of an element is a dict of clinician states and probabilities of decreased activation. If provided as a single float, the probabilitty of dispirit for an aware clinician and an owning patient is provided, and others calculated from that value. Default: TransitionDeterminer.default_dispirit() |
| 5. **patient_network_edges** (*float*) – how many other patients influence a patient, as an average across all patients? Note that influences are undirected; if patient Paul influences patient Patrick, then Patrick also influences Paul. Default: 0.0 |
| 6. **patient_influence_across_prop** (*float*) – what proportion of influence links between patients cross care center boundaries? Default: 0.0, a sensible default in the default case of only a single care center |
| 7. **patient_influence_become_aware_probability** (*float*) – if a patient can be influenced by other patients (i.e. **patient_influence_on** is True), and if a particular patient is unaware and one of its influencers is at least aware, what is the annual probability that the unaware patient will become aware because of the influence? Note the state transition may occur on any week, but the probability is specified annually. Default: 0.1 |
| 8. **patient_influence_activation_probability** (*float*) – if a patient can be influenced by other patients (i.e. **patient_influence_on** is True), and if a particular patient has an influencer that is of greater activation, what is the annual probability that the more highly activated influencer will cause the less activated patient to become more activated, because of the influence? Note the state transition may occur on any week, but the probability is specified annually. Default: 0.1 |
| 9. **clinician_network_edges** (*float*) – how many other clinicians influence a clinician, as an average across all clinicians? Note that influences are undirected; if clinician Claire influences clinician Claudia, then Claudia also influences Claire. Default: 0.0 |
| 10. **clinician_influence_across_prop** (*float*) – what proportion of influence links between clinicians cross care center boundaries? Default: 0.0, a sensible default in the default case of only a single care center. |
| 11. **clinician_influence_become_aware_probability** (*float*) – if a clinician can be influenced by |

another clinician (i.e. **clinician_influence_on** is True), and if a particular clinician is unaware and one of its influencers is at least aware, what is the annual probability that the unaware clinician will become aware because of the influence? Note the state transition may occur on any week, but the probability is specified annually. Default: 0.8

12. **clinician_influence_activation_probability** (*float*) – if a clinician can be influenced by another clinician (i.e. **clinician_influence_on** is True), and if a particular clinician has an influencer that is of greater activation, what is the annual probability that the more highly activated influencer will cause the less activated clinician to become more activated, because of the influence? Note the state transition may occur on any week, but the probability is specified annually. Default: 0.5

13. **clinician_dispirit_probability** (*float*) – what is the annual probability that a clinician who is at least participating will dispirit to a lower activation state? Note the state transition may occur on any week, but the probability is specified annually. Default: 0.1

14. **shared_knowledge_initial** (*float*) – how much shared knowledge is available in the learning network initially, as a commons across all agents? Shared knowledge is measured in units of contributions. When a contributing patient contributes to shared knowledge, he contributes 1 unit (by default). Default: 0.0

15. **shared_knowlege_half_life** (*float*) – if shared knowledge declines over time (i.e. **shared_knowledge_decays_on**), and if there are no new contributions to shared knowledge, how many weeks until the amount of shared knowledge has declined by half? Note that the decay of shared knowledge occurs whether new contributions are made or not, but it is more convenient to frame the half-life measure using the situation of no new contributions. Default: 153 (weeks)

16. **patient_shared_knowledge_contrib_determiner** (*ContributionDeterminer or dict[str, (float, float)] or int or float*) – if a patient might contribute shared knowledge (i.e. **patient_contributes_shared_knowledge_on** is True), how often will he contribute? And how much will that contribution increase the quantity of shared knowledge? If expressed as a dictionary instead of an instance of ContributionDeterminer, the key of each dict is a patient activation state (e.g. 'participating'), and the value of the dict is a tuple or list of the average period between shared knowledge contributions (in weeks), and the amount of shared knowledge contribution when it occurs. Alternate parameterization: an int or float for the owning period, with an assumption the contributing period is twice the owning, and the participating period is twice the contributing, and with one item with each contribution. Default: ContributionDeterminer.default_for_patients()

17. **clinician_shared_knowledge_contrib_determiner** – if a clinician might contribute shared knowledge (i.e. **clinician_contributes_shared_knowledge** is True), how often will she contribute? And how much will that contribution increase the quantity of shared knowledge? If expressed as a dictionary instead of an instance of ContributionDeterminer, the key of each dict is a clinician activation state (e.g. 'participating'), and the value of the dict is a tuple or list of the average period between shared knowledge contributions (in weeks), and the amount of shared knowledge contribution when it occurs. Alternate parameterization: an int or float for the owning period, with an assumption the contributing period is twice the owning, and the participating period is twice the contributing, and with one item with each contribution. Default: ContributionDeterminer.default_for_clinicians()

18. **enhanced_registry_initial_per_patient** (*int or None*) – how many records are in the enhanced registry at the beginning of the simulation, expressed per patient, as an alternative parameterization of **enhanced_registry_initial**. If not None, the value of

**enhanced_registry_initial** is ignored. Default: None, meaning use the value of **enhanced_registry_initial** instead.

19. **enhanced_registry_analysis_period** (*int*) – how often does is the enhanced registry analyzed, measured in weeks between analyses? Default: 13
20. **enhanced_registry_records_per_commons_item** (*int*) – when the enhanced registry is analyzed, how many records in the enhanced registry does it take to create a single item in the commons? Default: 1000
21. **potential_phenotype_response_info_from_SK_unit** (*float*) – how much does a single unit of shared knowledge increase the phenotype response info, assuming there is sufficient patient and clinician engagement? Default: 0.001
22. **phenotype_realization_numeric** (*float or None*) – how does patient engagement and clinician engagement affect the translation of shared knowledge to phenotype response info? This parameter is a numeric alternative to **phenotype_realization**, expressing the mix of clinician engagement and patient engagement that is used. A value of zero is the same as 'clinician_only'. A value of one is the same as 'patient_only'. A value of 0.5 is the same as 'even_both'. A non-None value is used instead of **phenotype_realization**. None indicates to use **phenothype_realization**. Default: None
23. **patient_response_info_half_life** (*float*) – if a patient is unaware and his clinician is also unaware, no additional patient response info will be added. In that situation, how many weeks until the level of patient response info has declined by half? Note that the decay of patient response info occurs whether or not there is new patient response info added, but it is convenient to frame the half-life measure using the situation of no new contribution. Default: 20 (weeks)
24. **patient_response_info_increase_numeric** (*float or None*) – how does patient engagement and clinician engagement combinge to affect the increase in patient response info? This parameter is a numeric alternative to **patient_response_info_increase**, expressing the mix of clinician engagement and patient engagement that is used. A value of zero is the same as 'clinician_only'. A value of one is the same as 'patient_only'. A value of 0.5 is the same as 'even_both'. A non-None value is used instead of **patient_response_info_incrase**. None indicates to use **patient_reponse_info_increase**. Default: None
25. **patient_response_info_acceleration_from_SK_unit** (*float*) – if the amount of shared knowledge affects the pace of increase of patient response info (i.e. **shared_knowledge_affects_patient_response_info_on** is True), how much does a single unit of shared knowledge accelerate the pace of shared patient response info increase? Default: 0.005
26. **maximal_patient_response_info_acceleration_from_SK** (*float*) – if the amount of shared knowledge affects the pace of increase of patient response info (i.e. **shared_knowledge_affects_patient_response_info_on** is True), and there is a sufficiently large amount of shared knowledge, what is the maximum acceleration of the pace of shared patient response info increase? Default: 0.5
27. **selection_efficiency_maximum** (*float*) – if praxis improves selection efficiency (i.e. **praxis_improves_selection_efficiency_on** is True), and if praxis for some patient is 1.0 (i.e. the clinician knows as much about that patient's condition and phenotype as it is possible to know), what is the selection efficiency for that clinician working with that patient? Default: 100
28. **evaluation_accuracy_minimum_praxis** (*float*) – if a clinician has zero (minimal) praxis with a patient, what is their evaluation accuracy in a clinical encounter? Default: 0.10
29. **patient_engagement_degree_participating** (*float*) – if a patient has an activation of participating, to what extent does he use the learning network to increase phenotype response

| | |
|---|---|
| | information and individual (patient) response information? Scale: zero to one. Default: 0.5 |
| 30. | **clinician_engagement_degree_participating** (*float*) – if a clinician has an activation of participating, to what extent does she use the learning network to increase phenotype response information and individual (patient) response information? Scale: zero to one. Default: 0.5 |
| | |
