# Supplementary material for: An agent-based model to advance the science of collaborative learning health systems
Source: PLoS One. 2025 Sep 9;20(9):e0332054. doi: 10.1371/journal.pone.0332054 (PMC12419628; doi:10.1371/journal.pone.0332054)
Supplement: S3 Supplement — (PDF) [file pone.0332054.s003.pdf]

```python
#!/usr/bin/env python3

"""model.py: modeling learning networks"""

__author__ = "Dave Bridgeland"
__copyright__ = "Copyright 2018-2024, Cincinnati Children's Hospital
Medical Center"
__credits__ = ["Dave Bridgeland", "Michael Seid", "David Hartley"]

__version__ = "0.2.0"
__maintainer__ = "Dave Bridgeland"
__email__ = "dave@hangingsteel.com"
__status__ = "Prototype"


import time
import math
import collections
import itertools
import functools
import random
import warnings
import statistics as stats
from numbers import Number

import pandas as pd
import numpy as np
import mesa
import mesa.time
import transitions

###

# Classes for agents: patients and clinicians

###

class EngagementLadder(transitions.Machine):
    """The state machine for agent states.

    Manages all state transitions. All agents inherit from here.

    :param initial_state: initial state of the agent, as a string.
    :type initial_state: str or None
    """
    def __init__(self, initial_state=None):
        super().__init__(
            self, self._states, initial=initial_state,
            after_state_change='_state_change', send_event=True,
            ignore_invalid_triggers=True)
```

```
        self.add_transition(
            'become_aware', 'unaware', 'aware',
conditions='_becomes_aware_now')
        self.add_transition(
            'activate', 'aware', 'participating',
conditions='_activates_now')
        self.add_transition(
            'activate', 'participating', 'contributing',
            conditions='_activates_now')
        self.add_transition(
            'activate', 'contributing', 'owning',
conditions='_activates_now')
        self.add_transition(
            'dispirit', 'owning', 'contributing',
conditions='_dispirits_now')
        self.add_transition(
            'dispirit', 'contributing', 'participating',
            conditions='_dispirits_now')
        self.add_transition(
            'dispirit', 'participating', 'aware',
conditions='_dispirits_now')

    _states = ['unaware', 'aware', 'participating', 'contributing',
'owning']
    def _state_change(self, event):
        """The agent just changed state. Record the change."""
        if event.transition.dest:
            agent = event.model
            agent.model.trace.trace_transition(
                agent, event.event.name, event.transition.source,
                event.transition.dest)

    @classmethod
    def states(cls):
        """Returns list of all possible states.

        :rtype: list of strings
        """
        return cls._states

    def progress(self):
        """How far along is this agent from unaware (0.0) to owning
(1.0)?

        :rtype: float
        """
        return {
            'unaware': 0.0,
            'aware': 0.25,
            'participating': 0.5,
```

```
            'contributing': 0.75,
            'owning': 1.0
        }[self.state]

    def less_activated_than(self, other_agent):
        """Does other_agent have a greater activation than this one?

        :param EngagementLadder other_agent: the agent to compare to
self
        :rtype: boolean
        """
        return other_agent.progress() > self.progress()

    def at_least(self, minimum_state):
        """Is the agent at least at this level of activation?

        Is the agent's state at least at the level of minimum_state?

        :param str minimum_state: one of the valid states, as a string
        :rtype: boolean
        """
        states = EngagementLadder.states()
        return states.index(self.state) >= states.index(minimum_state)

    def participatory_engagement(self):
        """What is the engagement, if the agent is at least
participating?"""
        if self.at_least('participating'):
            return self.engagement()
        else:
            return 0.0


class LearningNetworkAgent(mesa.Agent, EngagementLadder):
    """A person in a learning network.

    :param int agent_id: unique integer identifier of agent, typically
        generated in sequence
    :param LearningNetworkModel model: instance of model that includes
this
        agent
    :param initial_state: initial state of the agent, as a string.
    :type initial_state: str or None
    :param CareCenter care_center: instance of care center that
includes this
        agents

    """
    def __init__(self, agent_id, model, initial_state, care_center):
        self.unique_id = agent_id
```

```
        self._model = model
        self._influences = []
        if care_center:
            self.care_center = care_center
        EngagementLadder.__init__(self, initial_state=initial_state)

    @property
    def model(self):
        """The instance of :class:`LearningNetworkModel` that includes
agent."""
        return self._model

    def maybe_add_to_shared_knowledge_via_determiner(
            self, add_to_on, determiner):
        """The agent may add to shared knowledge this week.

        :param boolean add_to_on: whether to add to shared knowledge
ever. This
            is some **-on** parameter of :class:`LearningNetworkModel`
        :param ContributionDeterminer determiner: an object that knows
            whether shared knowledge should be added, and how much
        """
        if add_to_on:
            if determiner.does_contribute_now(self):
                amount = determiner.contribution_amount(self)
                self.model.shared_knowledge.increment(amount)
                self.model.trace.trace_shared_knowledge_change(
                    amount, self.model.shared_knowledge.level, self)

    def add_influence_edge(self, another_agent):
        """Add an edge between this agent and another.

        The agent keeps track of the agents it is influenced by. Add
another
        agent to that list.

        :param LearningNetworkAgent another_agent: the agent that is
to be
            added to the list of influencers
        """
        self._influences.append(another_agent)

    def remove_influence_edge(self, another_agent):
        """Remove edge from this agent to another.

        The agent keeps track of the agents it is influenced by.
Remove one
        of those agents from the list.

        :param LearningNetworkAgent another_agent: the agent that is
```

```
to be
            removed
        :raises ValueError: if another_agent is not an influencer of
this
            agent
        """
        self._influences.remove(another_agent)


class Patient(LearningNetworkAgent):
    """Modeling a single patient who suffers from some medical
condition.

    :param int patient_ID: unique integer identifier of patient,
typically
        generated in sequence. Note that these IDs are unique across
both
        patients and clinicians.
    :param LearningNetworkModel model: instance of model that includes
this
        patient
    :param Phenotype phenotype: the particular phenotype of this
patient. Note
        that this is not an observable characteristic in the real
world, and
        operates only behind the scenes in the model.
    :type clinician: either :class:`Clinician` or None
    :param initial_outcome: the initial numeric outcome of the
patient. Outcome
        will vary from week to week, and will change with treatment
    :type initial_outcome: float in [0.0, 1.0]
    :param str initial_state: the initial state of the patient on the
ladder of
        engagement, i.e. 'unaware', 'aware', 'participating',
'contributing',
        or 'owning'
    :param initial_patient_response_info: the initial degree to which
        a patient knows about his own condition, as a numeric measure.
Default:
        0.0
    :type initial_patient_response_info: float in [0.0, 1.0]
    :param encounter_period: how many weeks between clinician
encounters
        between this patient and a clinician. If None, the encounter
period
        is drawn randomly from integers between 10 and 16. Default:
None
    """
    def __init__(
        self,
```

```
        patient_ID,
        model,
        phenotype,
        initial_outcome,
        initial_state,
        care_center,
        initial_patient_response_info=0.0,
        encounter_period=None
    ):
        super().__init__(patient_ID, model, initial_state,
care_center)
        self._phenotype = phenotype
        # to do: abstract away the encounter period
        if encounter_period:
            self._encounter_period = encounter_period
        else:
            self._encounter_period = np.random.randint(10, 16)
        self._weeks_since_last_encounter = 0
        self._outcome = initial_outcome
        self._treatment_package = None
        self._patient_response_info = PatientResponseInfo(
            initial_patient_response_info, model)
        self._phenotype_response_info = PhenotypeResponseInfo(model,
self)
        self._previsit_constraints =
model.parameters.previsit_PRI_constraints
        self._records = MedicalRecord()

    def __str__(self):
        return 'Patient({})'.format(self.unique_id)

    @property
    def treatment_package(self):
        """Which treatment package is currently applied to the
patient?

        :returns: the current :class:`TreatmentPackage`, or None if no
treatment
            package is being applied
        """
        return self._treatment_package

    @property
    def clinician(self):
        """Which clinician is currently assigned to the patient?

        :returns: the assigned :class:`Clinician`, or None if no
clinician
            has been assigned yet"""
        return self._clinician
```

```
    @property
    def outcome(self):
        """What is the current numeric outcome of the patient?

        :returns: a float in [0.0, 1.0] measuring the current clinical
            condition of patient.
        """
        return self._outcome

    @property
    def perceived_outcome(self):
        """What is the current numeric outcome of the patient, as
perceived?

        :returns: a float in [0.0, 1.0] measuring the current clinical
            condition of patient, as perceived by the clinician during
the
            last clinical encounter
        """
        return self._perceived_outcome

    @property
    def previsit_constraints(self):
        """The dict of previsits and constraints on PRIs"""
        return self._previsit_constraints

    def assign_clinician(self, clinician):
        """Assign a clinician to the patient."""
        self._clinician = clinician

    def patient_previsit_planning(self):
        """How much previsit planning is performed by patient?

        :returns: a float in [0.1, 1.0] measuring how much previsit
planning
            is performed
        """
        # for now just use care center's institutional support for PVP
        # later we will add effect of patient engagment and maybe
clinician
        # engagement
        return
self.previsit_constraints[self.care_center.previsit_planning]

    def clinician_previsit_planning(self):
        """How much previsit planning is performed by clinician?

        :returns: a float in [0.1, 1.0] measuring how much previsit
planning
```

```
            is performed
        """
        # for now just use care center's institutional support for PVP
        # later we will add effect of clinician engagement
        return
self.previsit_constraints[self.care_center.previsit_planning]

    def patient_response_info(self):
        """How much is known about how this patient responds to
treatment?

        :returns: a float in [0.0, 1.0] measuring how much is known
about
            this patient's condition and how it responds to treatment.
        """
        return self._patient_response_info.level

    def phenotype_response_info(self):
        """How much is known about how patient's phenotype responds to
trtmn?

        :returns: a float in [0.0, 1.0] measuring how much is known
about
            how this patient's phenotype responds to treatment.
        """
        # This might could be modeled on the Phenotype instead
        return self._phenotype_response_info.level()

    def effective_patient_response_info(self):
        """How much is known during encounter about patient respons to
treatmnt?

        :returns: a float in [0.0, 1.0] measuring how much is known
during
            encounter about about this patient's conditions and how it
responds
            to treatment. Might be less than patient_response_info
because of
            lack of previsit planning.
        """
        return self._effective_response_info(
            self.patient_response_info(),
            self.patient_previsit_planning()
        )

    def _effective_response_info(self, response_info, pvp):
        """How much is known during encounter about individual or
phenotype?"""
        if self.model.parameters.previsit_planning_constrains_info_on:
            if (
```

```
self.model.parameters.realtime_usage_enhanced_registry_on and
                self.realtime_ehr_available()
            ):
                pvp = self._indicated_previsit_planning(pvp)
            return min(response_info, pvp)
        else:
            return response_info

    def realtime_ehr_available(self):
        """Is real-time enhanced registry avail 2 obviate pre-visit
planning?"""
        return self.care_center.realtime_ehr_available()

    def _indicated_previsit_planning(self, pvp_without_obviation):
        """How much previsit planning is performed, given
enhanced_registry?"""
        ehr_records = self.model.enhanced_registry.record_count
        leverage =
self.model.parameters.previsit_obviation_from_ehr_record
        # starts linear, then approaches max of 1.0
        obviation = 2.0 / (1.0 + math.exp(-2.0 * ehr_records *
leverage)) - 1.0
        return pvp_without_obviation + (1 - pvp_without_obviation) *
obviation

    def effective_phenotype_response_info(self):
        """How much is known during encounter about phenotype response
to trtmt?

        :returns: a float in [0.0, 1.0] measuring how much is known
during
            encounter about the phentotype response to treatment.
Might be
            less than phenotype_response_info because of lack of
previsit
            planning.
        """
        return self._effective_response_info(
            self.phenotype_response_info(),
            self.clinician_previsit_planning()
        )

    def praxis(self):
        """How much is known about patient?

        How much is known about this patient, for the purpose of
making a
        treatment decision? A combination of effective patient
response
```

```
        information and effecive phenotype response information.

        :returns:
        """
        usepat =
self.model.parameters.patient_response_info_affects_praxis_on
        usephe =
self.model.parameters.phenotype_response_info_affects_praxis_on
        patient_ri = self.effective_patient_response_info() if usepat
else 1
        phenotype_ri = self.effective_phenotype_response_info() if
usephe else 1
        return patient_ri * phenotype_ri

    def step(self):
        """What happens to the patient this week, and what does he do?

        Simulate the patient for one week. His outcome might change,
        praxis might be adjusted, he might add to shared knowledge, he
        might experience an encounter with his clinician, he might
transition
        his state, and he might leave the cohort."""
        # print(f"Step {str(self)}")
        self._adjust_outcome()
        self._update_patient_response_info()
        self._maybe_add_to_shared_knowledge()
        self._maybe_encounter()
        self._maybe_transition_state()
        self._maybe_leave_cohort()

    def _maybe_add_to_shared_knowledge(self):
        """The patient might add to shared knowledge now."""
        params = self.model.parameters
        self.maybe_add_to_shared_knowledge_via_determiner(
            params.patient_contributes_shared_knowledge_on,
            params.patient_shared_knowledge_contrib_determiner)

    def _maybe_encounter(self):
        """If it is time for a clinician enounter, encounter."""
        self._weeks_since_last_encounter += 1
        if self._weeks_since_last_encounter >= self._encounter_period:
            self._encounter_this_week = True
            self._encounter()
            self._weeks_since_last_encounter = 0

    def _encounter(self):
        """Patient has a medical encounter with his clinician."""
        # to do: log this
        # print('Encounter between {} and {}'.format(self,
self._clinician))
```

```
        po = np.random.normal(
            self._outcome,
            self._clinician.evaluation_accuracy(self)
        )
        self._perceived_outcome = max(0, min(1, po))
        self._records.update_records(
            self._perceived_outcome,
            self.model.clock.current_week
        )
        self._maybe_adjust_treatment_package()
        self.care_center.maybe_contribute_to_ehr(self.model)
        self.model.trace.trace_encounter(self, self._clinician)

    def _maybe_adjust_treatment_package(self):
        """Change the treatment package, if necessary."""
        # Should be moved to Clinician
        clinician = self._clinician
        trace = self.model.trace
        old_tp = self.treatment_package
        if old_tp is None:
            self._treatment_package =
clinician.initial_treatment_package(self)
            trace.trace_treatment_package(self, 'none',
self._treatment_package)
        elif not self._records.is_patient_improving():
            self._treatment_package =
clinician.changed_treatment_package(self)
            trace.trace_treatment_package(self, old_tp,
self._treatment_package)

    def _maybe_transition_state(self):
        """At a clinician encounter, chance of patient state
transition."""
        # must be called after _maybe_encounter(), not before
        self.become_aware() or self.activate() or self.dispirit()

    def _becomes_aware_now(self, event_data):
        """Does the patient become aware now?"""
        params = self.model.parameters
        via_clinician =
self._becomes_aware_via_clinical_encounter(params)
        via_other_patient =
self._becomes_aware_via_patient_influence(params)
        return via_clinician or via_other_patient

    def _becomes_aware_via_clinical_encounter(self, params):
        """Does the patient become aware this wk via a clinician
encounter?"""
        return (
            params.encounter_aware_on and
```

```
            self._encounter_occurs_this_week() and
            self.state == 'unaware' and
            self._clinician.may_cause_patient_awareness() and
            np.random.random() < params.encounter_aware_likelihood
        )

    def _becomes_aware_via_patient_influence(self, params):
        """Does the patient become aware this wk via patient
influence?"""
        if params.patient_influence_on:
            wkly_p =
params.patient_become_aware_weekly_prob_per_aware_infl()
            for p in self._influences:
                if p.at_least('aware') and np.random.random() <
wkly_p:
                    return True
        return False

    def _activates_now(self, event_data):
        """Does the patient activate now?"""
        params = self.model.parameters
        return (
            self._activates_now_via_clinical_encounter(params) or
            self._activates_now_via_patient_influence(params)
        )

    def _activates_now_via_clinical_encounter(self, params):
        """Does the patient activate this wk via a clinical
enocunter?"""
        return (
            params.encounter_activate_on and
            self._encounter_occurs_this_week() and

params.patient_activate_determiner.does_transition_now(self)
        )

    def _activates_now_via_patient_influence(self, params):
        """Does the patient activate this wk via patient influence?"""
        if params.patient_influence_on:
            wkly_p =
params.patient_activates_weekly_prob_per_pos_influence()
            for p in self._influences:
                if self.less_activated_than(p) and np.random.random()
< wkly_p:
                    return True
        return False

    def _dispirits_now(self, event_data):
        """Does the patient dispirit now?"""
        params = self.model.parameters
```

```
        return (params.encounter_dispirit_on and
                self._encounter_occurs_this_week() and
                
params.patient_dispirit_determiner.does_transition_now(self))

    def _encounter_occurs_this_week(self):
        """Did the clinical encounter occur this week?"""
        return self._weeks_since_last_encounter == 0

    def _adjust_outcome(self):
        """Adjust the current outcome based on effectiveness of 
treatment."""
        self._outcome = np.clip(
            self._outcome +
                self._improvement_from_treatment_package() +
                self._random_outcome_change() +
                self._relapse_outcome_change(),
            0, 1)

    def _improvement_from_treatment_package(self):
        """How much does outcome improve this week due to treatment 
package?"""
        if self._treatment_package is None:
            return 0
        elif self.model.parameters.treatment_package_on:
            impr = 
self.treatment_package_effectiveness(self.treatment_package)
            # print(f"Improved {str(self)} by treatment package 
{impr}")
            return impr
        else:
            return 0

    def _random_outcome_change(self):
        """How much does the outcome change this week for random 
reasons?"""
        params = self.model.parameters
        if params.random_walk_on:
            change = np.random.normal(
                params.random_walk_mean,
                params.random_walk_sd
            )
            # print(f"Changed {str(self)} by random {change}")
            return change
        else:
            return 0.0

    def _relapse_outcome_change(self):
        """How much does the outcome change if the patient 
relapses?"""
```

```
        params = self.model.parameters
        if not params.relapse_on:
            return 0.0
        elif np.random.random() < params.relapse_probability:
            self.model.trace.trace_relapse(self, self.outcome)
            amount = -params.relapse_amount
            # print(f"{str(self)} relapsed by {amount}")
            return amount
        else:
            return 0.0

    def _update_patient_response_info(self):
        """Update the patient response info of the patient."""
        self._patient_response_info.step(self)

    def _maybe_leave_cohort(self):
        """The patient may leave the cohort."""
        def log_patient_departure(reason):
            pass
            # print(f'{self} in care center {self.care_center}
{reason}')

        if self._age_out_p():
            log_patient_departure('aged out')
            self._leave_cohort('aged out')
        elif self._move_away_p():
            log_patient_departure('moved away')
            self._leave_cohort('moved away')
        elif self._got_better_p():
            log_patient_departure('got better')
            self._leave_cohort('got better')
        elif self._got_worse_p():
            log_patient_departure('got worse')
            self._leave_cohort('got worse')

    def _age_out_p(self):
        """Does the patient age out of the cohort?"""
        params = self.model.parameters
        return (params.cohort_exit_on and
                np.random.random() <
params.age_out_weekly_proportion())

    def _move_away_p(self):
        """Does the patient move away this week?"""
        params = self.model.parameters
        return (params.cohort_exit_on and
                np.random.random() <
params.move_away_weekly_proportion())

    def _got_better_p(self):
```

```
        """Does the patient leave this week because he is better?"""
        return (
            self.model.parameters.cohort_exit_on and
            np.random.random() < self._weekly_get_better_chance()
        )

    def _weekly_get_better_chance(self):
        """What is the chance that this patient will get better this
week?"""
        return _annual_to_weekly(self._annual_get_better_chance())

    def _annual_get_better_chance(self):
        """What is the chance that the patient will get better this
year?"""
        params = self.model.parameters
        return (
            params.get_better_annual_proportion *
            self._progress_along(
                self.outcome, params.get_better_start_outcome, 1.0) )

    def _got_worse_p(self):
        """Does the patient leave the cohort this week because he is
worse?"""
        return (
            self.model.parameters.cohort_exit_on and
            np.random.random() < self._weekly_get_worse_chance()
        )

    def _weekly_get_worse_chance(self):
        """What is the chance this patient will get worse this
week?"""
        return _annual_to_weekly(self._annual_get_worse_chance())

    def _annual_get_worse_chance(self):
        """What is the chance the patient will leave in a yaer?"""
        params = self.model.parameters
        return (
            params.get_worse_annual_proportion *
            self._progress_along(
                self.outcome, params.get_worse_end_outcome, 0.0))

    def _leave_cohort(self, reason):
        """The patient leaves the cohort."""
        self._remove_influence_of_exiting_patient
        self.model.trace.trace_cohort_exit(self, reason, self.outcome)
        self.model.remove_patient_from_cohort(self)

    def _remove_influence_of_exiting_patient(self):
        """Remove patient from any influence edges."""
        for p in self._influences:
```

```
            p.remove_influence_edge(self)

    def current_status(self):
        """The current status of patient, suitable for history and
traces.

        :returns: the outcome, state, clinician and praxis of patient
        :rtype: dict
        """
        status = {
            'outcome': self.outcome,
            'state': self.state,
            'clinician': self.clinician,
            'praxis': self.praxis(),
            'care center': self.care_center.id
        }
        try:
            status['perceived_outcome'] = self._perceived_outcome
        finally:
            try:
                status['treatment_package'] = self._treatment_package
            finally:
                return status

    def treatment_package_effectiveness(self, treatment_package):
        """The potential effectiveness of a treatment_package to this
patient.

        How effective is a treatment package, in terms of
        outcome change per week? Note that this is hidden knowledge in
the real
        world. In the model, the knowledge is used in the clinician
mapping
        from selection efficiency to choice of treatment package.

        :param TreatmentPackage treatment_package: which treatment
package to
            evaluate
        :returns: outcome change per week for this patient
        :rtype: float in [-1.0, 1.0], although typically close to 0.0
        """
        return treatment_package.outcome_improvement(self._phenotype)

    @staticmethod
    def _progress_along(traveler, from_num, to_num):
        """How far is the traveler num along path from from_num to
to_num?"""
        return max(0.0, min(1.0, (traveler - from_num) / (to_num -
from_num)))
```

```
    def engagement(self):
        """To what extent does the patient participate in learning
network?"""
        return
self.model.parameters.patient_engagement_degree[self.state]


class Clinician(LearningNetworkAgent):
    """Modeling a clinician who treats patients within a learning
network.

    :param int clinician_id: unique integer identifier of clinician,
        typically generated in sequence. Note that these IDs are
unique across
        both patients and clinicians.
    :param LearningNetworkModel model: instance of model that includes
this
        clinician
    :param str initial_state: the initial state of the clinician on
the
        ladder of engagement, i.e.  'unaware', 'aware',
'participating',
        'contributing', or 'owning'
    :param CareCenter care_center: the care_center where the clinician
        practices
    """
    def __init__(self, clinician_id, model, initial_state,
care_center):
        super().__init__(clinician_id, model, initial_state,
care_center)

    def __str__(self):       # pragma: no cover
        return "Clinician({})".format(self.unique_id)

    def step(self):
        """What does the clinician do this week?

        Simulate the clinician for one week. She might transition
state,
        and she might add to shared knowledge. (Encounters with
patients
        are simulated as part of :meth:`Patient.step`.)
        """
        # print(f"Step {str(self)}")
        self._maybe_transition_state()
        self._maybe_add_to_shared_knowledge()

    def _maybe_transition_state(self):
        """Each week, a clinician may change state."""
        self.become_aware() or self.activate() or self.dispirit()
```

```
    def _becomes_aware_now(self, event_data):
        """Does the clinician become aware this week?"""
        params = self.model.parameters
        if params.clinician_influence_on:
            wkly_p =
params.clinician_become_aware_weekly_prob_per_aware_infl()
            for c in self._influences:
                if c.at_least('aware') and np.random.random() <
wkly_p:
                    return True
        return False

    def _activates_now(self, event_data):
        """Does the clinician activate now?"""
        params = self.model.parameters
        if params.clinician_influence_on:
            wkly_p =
params.clinician_activates_weekly_prob_per_pos_influence()
            for c in self._influences:
                if self.less_activated_than(c) and np.random.random()
< wkly_p:
                    return True
        return False

    def _dispirits_now(self, event_data):
        """Does the clinician dispirit now?"""
        params = self.model.parameters
        return (
            params.clinician_dispirit_on and
            np.random.random() <
params.clinician_dispirit_weekly_probability()
        )

    def _maybe_add_to_shared_knowledge(self):
        """The clinician may add to shared knowledge this week."""
        params = self.model.parameters
        self.maybe_add_to_shared_knowledge_via_determiner(
            params.clinician_contributes_shared_knowledge_on,
            params.clinician_shared_knowledge_contrib_determiner)

    def initial_treatment_package(self, patient):
        """Determine treatment package for patient.

        The patient currently has no treatment package. Select one.

        :param Patient patient: the patient who needs a treatment
package
        :rtype: TreatmentPackage
        """
```

```
        return self._select_treatment_package(
            patient, self.model.treatment_packages)

    def changed_treatment_package(self, patient):
        """Determine a better (or just different) treatment package
for patient.

        The patient currently has a treatment package, but it needs to
be
        changed to a different one. If there is only a single
treatment
        package available, that one must be used.

        :param Patient patient: the patient who needs a different
treatment
            package
        :rtype: TreatmentPackage
        """
        remaining_treatment_packages = [
            tp for tp in self.model.treatment_packages
            if tp is not patient.treatment_package]
        if remaining_treatment_packages:
            return self._select_treatment_package(
                patient, remaining_treatment_packages)
        else:
            # cannot change as there is no other one to choose
            return patient.treatment_package

    def _select_treatment_package(self, patient, tps):
        """Select a treatment package for the patient, from the ones
in tps."""
        params = self.model.parameters
        if params.praxis_improves_selection_efficiency_on:
            return self._choice_weighted(
                tps, self._treatment_package_probabilities(patient,
tps))
        else:
            return np.random.choice(tps)

    @staticmethod
    def _choice_weighted(seq, weights):
        """Return a member of seq, randomly and weighted."""
        sumweight = sum(weights)
        p = [w/sumweight for w in weights]
        return np.random.choice(seq, p=p)

    def _treatment_package_probabilities(self, patient,
treatment_packages):
        """Return the probabilities of selecting each treatment
package."""
```

```
        effectivenesses = [
            patient.treatment_package_effectiveness(tp)
            for tp in treatment_packages]
        selection_efficiency = self.selection_efficiency(patient)
        attractivenesses = [
            math.exp(selection_efficiency * eff) for eff in
effectivenesses]
        total_attractiveness = sum(attractivenesses)
        return [att / total_attractiveness for att in
attractivenesses]

    def selection_efficiency(self, patient):
        """Return the selection efficiency, based on praxis.

        The selection efficiency is how well this clinician selects a
        treatment for this patient. For a particular clinician,
selection
        efficiency will differ from patient to patient, based on the
praxis
        for that patient.

        A greater selection efficiency means that the clinician is
more likely
        to select an effective treatment package for the patient, and
less
        likely to select an ineffective treatment package. The minimum
        possible selection efficiency is 0.0, which means even odds:
the
        clinician is just as likely to select an ineffective (or
        countereffective) treatment package as an effective one.

        If there are two treatment packages with effectiveness F1 and
F2,
        and F1 is more effective than F2 by a difference in
effectiveness
        of d, and if selection efficiency is 1.0, then the odds of
selecting
        F1 instead of F2 is (1 + d). If selection efficiency is 2.0,
then
        the odds are (1 + d)^2. In fact the odds increase by a factor
of
        (1 + d) for each additional unit of selection efficiency.

        Praxis is how much is known about the patient, for the purpose
of
        making a treatment decision, and is measured on a scale [0.0,
1.0].

        There is some minimum selection efficiency if a clinician
knows
```

```
        nothing about a patient, e.g. he is a new patient in the
clinician's
        solo practice. There is a maximum selection efficiency, the
        selection efficiency if the clinician has a praxis of 1.0 for
this
        patient.

        The selection efficiency for a patient with some praxis in
between
        0.0 and 1.0 is modeled as a linear function of the praxis,
from
        the minimum selection efficiency to the maximum.

        :param Patient patient: the patient for whom a treatment
package is
            selected
        :returns: selection efficiency for this patient
        :rtype: float
        """
        params = self.model.parameters
        return self._proportion_along(
            params.selection_efficiency_minimum,
            params.selection_efficiency_maximum,
            softmin1(patient.praxis())
        )

    def current_status(self):
        """The current status of clinician, suitable for history and
traces.

        :returns: the state of the clinician
        :rtype: dict
        """
        return {'state': self.state, 'care center':
self.care_center.id}

    def may_cause_patient_awareness(self):
        """Could the clinician cause the patient to become aware?

        Might the clinician cause a patient to become aware of the
learning
        network, i.e. is the clinician in a state that could so
educate the
        patient?

        :rtype: boolean
        """
        return self.state in ['aware','contributing', 'participating',
'owning']
```

```
    @staticmethod
    def _proportion_along(min, max, prop):
        """Return the quantity that is prop along the path from min to
max."""
        return min + prop * (max - min)

    def evaluation_accuracy(self, patient):
        """How accurate are the observations of outcome?

        The evaluation accuracy is the degree to which perceived
outcome
        is similar to true (unobserved) outcome. Evaluation accuracy
is
        measured as the standard deviation of the perceived outcome,
where
        the true outcome is the mean. Both true outcomes and perceived
outcomes
        are measured on zero to one scales, so perceived outcome is
truncated by
        by the limit of the scale.

        :param Patient patient: the patient for whom we are
considering
            evaluation accuracy
        :returns: a float in [0.0, 1.0], measuring standard deviation
of
            difference betweeen perceived_outcome and outcome
        """
        params = self.model.parameters
        if params.praxis_improves_evaluation_accuracy_on:
            return self._proportion_along(
                params.evaluation_accuracy_minimum_praxis,
                params.evaluation_accuracy_maximum_praxis,
                softmin1(patient.praxis())
            )
        else:
            return params.evaluation_accuracy_minimum_praxis

    def engagement(self):
        """To what extent does the clinician participate in learning
network?"""
        return
self.model.parameters.clinician_engagement_degree[self.state]

class CareCenter:
    """Modeling a care center that employs clinicians and cares for
patients.

    A care center tracks three different patient costs:
        * the initial specification of how many patients are in the
```

```
care center
        * how many of the initial patients are still to be added, in
the case that we are adding patients progressively over some weeks
        *  how many patients are in the care center now, including the
initial patients already added and any patients who arrived later

    :param int patient_count: initial specification of how many
patients will
        this care center simulate care for?
    :param int cliniciant_count: how many clinicians are in this care
center
        treating patients?
    :param str assignment_spec: how are clinicians assigned to
patients?
        Valid alternatives:

            * 'random': each patient is assigned a clinician randomly
from the set of clinicians.
            * 'successive': the first patient is assigned the first
clinician, the second patient is assigned the second clinician, and so
on. After the last clinician is assigned, the first clinician is
assigned again, to the next patient, repeating in a cycle.

    :param int id: unique ID of the care center
    :param str previsit_planning: level of support for previsit
planning. 'low',
        'medium', or 'high'. Default: 'high'
    :param str enhanced_registry: support for enhanced registry,
either
        'no contribution', 'contribution', or
        'contribution and real-time usage'. Default: 'no contribution'
    """
    def __init__(
        self,
        patient_count,
        clinician_count,
        assignment_spec,
        id,
        previsit_planning="low",
        enhanced_registry="no contribution",
    ):
        # the counts are prospective, assigned berfore any patient
added
        self.planned_patient_count = patient_count
        self.planned_clinician_count = clinician_count
        self.id = id
        self.patients = []
        self.clinicians = []
        self._assignment_spec = assignment_spec
        self._clinician_assigner = None # created after clinicians are
```

```
known
        self.previsit_planning = previsit_planning
        self._open_spots = 0
        self._enhanced_registry = enhanced_registry

    @property
    def contribute_to_ehr(self):
        return (
            self._enhanced_registry == "contribution" or
            self._enhanced_registry == "contribution and real-time
usage"
        )

    def realtime_ehr_available(self):
        """Is real-time usage of enhanced registry available?"""
        return self._enhanced_registry == "contribution and real-time
usage"

    def add_clinician(self, clinician):
        """Add a clinician to the care center."""
        if self._clinician_assigner is not None:
            raise LearningNetworkModelException(
                "Attempt to add clinician after assigner is created"
            )
        self.clinicians.append(clinician)

    def add_patient(self, patient):
        """Add a patient to the care center."""
        self.patients.append(patient)
        self._create_assigner_if_necessary()
        patient.assign_clinician(next(self._clinician_assigner))

    def _create_assigner_if_necessary(self):
        """Create an assigner for clinicians, if does not yet exist"""
        # Note that this depends on all the clinicians being known by
now.
        if self._clinician_assigner is None:
            self._clinician_assigner = {
                'random': iter(lambda:
np.random.choice(self.clinicians), 2 ),
                'successive': itertools.cycle(self.clinicians)
            }[self._assignment_spec]

    def remove_patient(self, patient):
        """Remove patient from the care center, e.g. if patient ages
out"""
        self.patients.remove(patient)
        self._open_spots = self._open_spots + 1

    def patient_count(self):
```

```
        """How many patients?"""
        return len(self.patients)

    def clinician_count(self):
        """How many clinicians?"""
        return len(self.clinicians)

    def maybe_replace_departures(self, model):
        """If there have been any departures, maybe replace those
patients"""
        for _ in range(self._open_spots):
            model._add_patient('replace', care_center=self)
        self._open_spots = 0

    def maybe_contribute_to_ehr(self, model):
        """If this care center contributes to the enhanced registry,
do so"""
        if self.contribute_to_ehr:
            model.enhanced_registry.add_encounter()


class CareCenterDesignator:
    """For determining the care center for a patient or a
clinician."""
    def __init__(self, cc_list, agent_type="patient"):
        count_attr = f"planned_{agent_type}_count"
        self._initialize_counts_initial_cohort(cc_list, count_attr)
        self._initialize_probs_later_entrants(cc_list, count_attr)

    def _initialize_counts_initial_cohort(self, cc_list, count_attr):
        """Initialize counts used for initial cohort"""
        self._to_designate = {
            cc: getattr(cc, count_attr)
            for cc in cc_list
            if getattr(cc, count_attr) > 0
        }
        self._remaining_count = sum(self._to_designate.values())

    def _initialize_probs_later_entrants(self, cc_list, count_attr):
        """Initialize probs used for agents entering cohort later"""
        self._care_centers = cc_list.copy()
        total_count = sum(getattr(cc, count_attr) for cc in cc_list)
        self._probabilities = [
            getattr(cc, count_attr) / total_count
            for cc in cc_list
        ]

    def next(self, reason='initial'):
        """Return the next care center randomly, given counts for
each."""
```

```
        if reason == 'initial':
            return self._next_initial_cohort()
        else:
            return self._next_subsequent_entrant()

    def _next_initial_cohort(self):
        """Return the next care center, according to initial cohort
counts"""
        index = np.random.randint(0, self._remaining_count)
        for cc, rem in self._to_designate.items():
            if index < rem:
                # could be optimized by removing zeroed out care
centers
                self._to_designate[cc] -= 1
                self._remaining_count -= 1
                return cc
            else:
                index -= rem
        raise LearningNetworkModelException("did not find care
center")

    def _next_subsequent_entrant(self):
        """Return the next  are center, according to probabilities"""
        choice = np.random.choice(
            len(self._care_centers), p=self._probabilities
        )
        return self._care_centers[choice]

#
# Classes for creating agents: creating agents and creating clinicians
#

class _AgentFactory:
    """For creating agents via a set of specs."""
    def __init__(self, model, first_id, state_spec):
        self._id = itertools.count(first_id)
        self._model = model
        self._state = self._state_iterator(state_spec)

    def _state_iterator(self, spec):
        """Return an iterator for agent states, based on spec."""
        if isinstance(spec, str):
            return {
                'all_unaware': itertools.repeat('unaware'),
                'all_aware': itertools.repeat('aware'),
                'all': itertools.cycle(EngagementLadder.states()),
                'all_except_unaware': itertools.cycle([
                    state for state in EngagementLadder.states()
                    if state != 'unaware']),
                'random_choice': iter(
```

```
                    lambda:
np.random.choice(EngagementLadder.states()),
                    "never"
                )
                # 'power_law_80': 'not yet implmented'
            }[spec]
        elif isinstance(spec, list):
            return itertools.cycle(spec)
        else:
            states = EngagementLadder.states()
            probs = [spec[s] if s in spec else 0.0 for s in states]
            return iter(lambda: np.random.choice(states, p=probs),
"never")


class PatientFactory(_AgentFactory):
    """For creating patients via a set of patient creation specs.

    The factory is initialized with a set of patient creation specs.
Then
    each call to factory.create() creates a new patient.

    :param LearningNetworkModel model: the instance of the model that
is to
        include all the patients to be created
    :param int first_id: the ID of the first patient to be created.
The
        second patient will have an ID of first_id + 1, the next one
will be
        first_id + 2, and so on.
    :param outcome_spec: the probability distribution of the
        initial patient outcomes. Valid alternatives:

            * 'uniform_0_1': uniform distribution between 0.0 and 1.0
            * 'uniform_08_1': uniform distribution between 0.8 and 1.0
            * 'uniform_0_03': uniform distribution between 0.0 and 0.3
            * 'beta_2_5': a beta distribution with alpha=2 and beta=5.
That distribution has a mode of 0.2 and a median of roughly 0.26
            * 'beta_3_4': a beta distribution with alpha=3 and beta=4
            * 'beta_3-5_3-5': a beta distribution with alpha and beta
both 3.5
            * 'beta_4_3': a beta distribution with alpha=4 and beta=3
            * 'beta_5_2': a beta distribution with alpha-5 and beta=2
            * a list of floats (e.g. [0.0, 0.4, 0.6]), indicating that
outcomes should cycle through the list, one patient at a time.
    :type outcome_spec: str or list of floats
    :param state_spec: what initial state is assigned to each patient?
        Valid options:

            * 'all_unaware': each patient is initially unaware
```

```
            * 'all_aware': each patient is initially aware
            * 'all': first patient is unaware, the second is aware,
third is participating, fourth contributing, fifth owning, then the
sixth is aware again, and so on, in a cycle
            * 'all_except_unware': first patient is aware, second is
participating, third contributing, fourth owning, then the fifth is
aware again, and so on, in a cycle
            * 'random_choice': each patient is assigned an initial
state among the five possible states, randomly
            * list of states: the first patient is assigned the first
state in the list, the second patient is assigned the second state in
the list, and so on, cycling as needed
            * dict of states and probabilities for each
    :type state_spec: str or list of states (as strings)

    """
    def __init__(self, model, first_id, outcome_spec, state_spec):
        super().__init__(model, first_id, state_spec)
        self._outcome = self._outcome_iterator(outcome_spec)

    def create(self, care_center):
        """Create a new patient.

        :rtype: Patient
        """
        params = self._model.parameters
        patient = Patient(
            next(self._id),
            self._model,
            np.random.choice(self._model._phenotypes),
            next(self._outcome),
            next(self._state),
            care_center,

initial_patient_response_info=params.patient_response_info_initial,
            encounter_period=params.encounter_period
        )
        care_center.add_patient(patient)
        return patient

    def _outcome_iterator(self, spec):
        """Return an iterator for initial outcomes, based on spec."""
        try:
            return {
                'uniform_0_1': iter(np.random.random, 2),
                'beta_2_5': iter(lambda: np.random.beta(2, 5), 2),
                'beta_3_4': iter(lambda: np.random.beta(3, 4), 2),
                'beta_3-5_3-5': iter(lambda: np.random.beta(3.5, 3.5),
2),
                'beta_4_3': iter(lambda: np.random.beta(4, 3), 2),
```

```
                'beta_5_2': iter(lambda: np.random.beta(5, 2), 2),
                'uniform_08_1': iter(lambda: np.random.random() * 0.2
+ 0.8, 2),
                'uniform_0_03': iter(lambda: np.random.random() * 0.3,
2)
            }[spec]
        except TypeError:
            return itertools.cycle(spec)


class ClinicianFactory(_AgentFactory):
    """For creating clinicians via a set of clinician creation specs.

    The factory is initialized with a specs. Then each call to
factory.create()
    creates a new clinician.

    :param LearningNetworkModel model: the instance of the model that
is to
        include all the clinicians to be created
    :param int first_id: the ID of the first clinician to be created.
The
        second clinician will have an ID of first_id + 1, the next one
will be
        first_id + 2, and so on.
    :param state_spec: what initial state is assigned to each
clinician?
        Valid options:

            * 'all_unaware': each clinician is initially unaware
            * 'all_aware': each clinician is initially aware
            * 'all': first clinician is unaware, the second is aware,
third is participating, fourth contributing, fifth owning, then the
sixth is aware again, and so on, in a cycle
            * 'all_except_unware': first clinician is aware, second is
participating, third contributing, fourth owning, then the fifth is
aware again, and so on, in a cycle
            * 'random_choice': each clinician is assigned an initial
state among the five possible states, randomly
            * list of states: the first clinician is assigned the
first state in the list, the second clinician is assigned the second
state in the list, and so on, cycling as needed
            * dict of states and probabilities for each
    :type state_spec: str or list of states (as strings)
    """
    def create(self, care_center):
        """Create a new clinician.

        :param CareCenter care_center: the care center where the newly
created
```

```
            clinician practices
        :rtype: Clinician
        """
        clinician = Clinician(
            next(self._id), self._model, next(self._state),
care_center
        )
        care_center.add_clinician(clinician)
        return clinician

#
# PatientResponseInfo and PhenotypeResponseInfo
#

class _ResponseInfo:
    """Common elements of PatientResponseInfo and
PhenotypeResponseInfo."""
    def __init__(self, model, aggregation_param):
        self._shared_knowledge = model.shared_knowledge
        self._model_parameters = model.parameters
        self._aggregation = PatientClinicianTeam(
            self._model_parameters, aggregation_param)


class Stock:
    """A system dynamics abstract class.

    Any subclass must implement change(), to implement how the stock
increases
    or declines this step.

    :param float initial_level: the initial value for the stock.
Default: 0.0
    """
    def __init__(self, initial_level=0.0):
        self._level = initial_level

    @property
    def level(self):
        """Current level of the stock.

        :rtype: float
        """
        return self._level

    def step(self, *args):
        """Change the level of the stock, as another time unit
passed."""
        self._level += self.change(*args)
```

```
    def increment(self, amount):
        """Increment level by amount"""
        self._level += amount


class PatientResponseInfo(_ResponseInfo, Stock):
    """Amount of patient response information that a patient currently
has.

    More precisely: the amount of patient response information that a
    clinician has about a patient, assisted by what the patient is
collecting
    about himself. The level will vary week-by-week from 0.0 to 1.0.

    :param float initial_level: the initial amount of patient response
into
    :param LearningNetworkModel model: the model that this patient is
part of

    """
    def __init__(self, initial_level, model):
        if model.parameters.patient_response_info_increase_numeric is
None:
            _ResponseInfo.__init__(
                self, model, 'patient_response_info_increase'
            )
        else:
            _ResponseInfo.__init__(
                self, model, 'patient_response_info_increase_numeric'
            )
        Stock.__init__(self, initial_level)

    # maximum increase per week, to make this a 0 to 1ish scale
    # To do: expose this as a model parameter
    _max_increase = 1 / 30.0

    @property
    def level(self):
        """Current level of patient response info. No more than 1"""
        return softmin1(self._level)

    def change(self, patient):
        """Another week has passed. Determine change in patient
response info.

        :param Patient patient: the patient whose response info has
changed
        :param Clinician clinician: the clinician whose understanding
of the
            patient may have changed (e.g. during an encounter)
```

```
        """
        return self._increase(patient) - self._decay()

    def _decay(self):
        """How much does patient response info decay this week?"""
        return (
            self._level *
self._model_parameters.patient_response_info_decay_prop_per_week
        )

    def _increase(self, patient):
        """How much does patient response info increase this week?"""
        return (
            self._aggregation.aggregate(patient) *
            (1 + self._increase_acceleration()) *
            self._max_increase
        )

    def _increase_acceleration(self):
        """How much quicker does PRI increase due to shared
knowledge?"""
        params = self._model_parameters
        if params.shared_knowledge_affects_patient_response_info_on:
            unit_accel =
params.patient_response_info_acceleration_from_SK_unit
            max_accel
=params.maximal_patient_response_info_acceleration_from_SK
            sk = self._shared_knowledge.level
            return (
                max_accel *
                (2.0 / (1.0 +
                        math.exp(-2.0 * sk * unit_accel/max_accel)) -
1.0)
            )
        else:
            return 0


class PhenotypeResponseInfo(_ResponseInfo):
    """Amount of information about a particular phenotype.

    Level varies from week to week within [0.0, 1.0].

    :param LearningNetworkModel: the model that this phenotype is part
of
    :param Patient patient: the patient who is of this phenotype
    """
    def __init__(self, model, patient):
        if model.parameters.phenotype_realization_numeric is None:
```

```
            super().__init__(model, 'phenotype_realization')
        else:
            super().__init__(model, 'phenotype_realization_numeric')
        self._patient = patient
        self._model = model

    def level(self):
        """What is the current value of phenotype response info?

        :returns: the current amount of phenotype response info,
between 0.0
            and 1.0
        :rtype: float
        """
        return (
            self._potential_phenotype_response_info() *
            self._proportion_of_potential_realized()
        )

    def _potential_phenotype_response_info(self):
        """Return possible phenottype response info, based on shared
knowledge.

        What phenotype response info is possible with sufficient
engagement
        of patient and clinician?
        """
        # starts linear, then approaches a max of 1.0
        sk = self._shared_knowledge.level
        params = self._model_parameters
        pri_leverage =
params.potential_phenotype_response_info_from_SK_unit
        return 2.0 / (1.0 + math.exp(-2.0 * sk * pri_leverage)) - 1.0

    def _proportion_of_potential_realized(self):
        """How much of potential phenotype response info is
realized?"""
        realization_from_activation =
self._aggregation.aggregate(self._patient)
        if (
            self._model_parameters.realtime_usage_enhanced_registry_on
and
            self._patient.realtime_ehr_available()
        ):
            return self._substitute_ehr_for_activation(
                realization_from_activation
            )
        else:
            return realization_from_activation
```

```
    def _substitute_ehr_for_activation(self, realization):
        """Substitute EHR records in whole or in part for
activation"""
        ehr_records = self._model.enhanced_registry.record_count
        leverage =
self._model.parameters.activation_obviation_from_ehr_record
        # starts linear, then approaches max of 1.0
        # could be DRYed out
        obviation = 2.0 / (1.0 + math.exp(-2.0 * ehr_records *
leverage)) - 1.0
        return realization + (1 - realization) * obviation


class PatientClinicianTeam:
    """Combining the engagement of patient and clinician in different
ways.

    Both clinician engagement and patient engagtement are important to
how
    response info increases over time. How important?
    This class manages aggregating the engagement of the two, for the
benefit
    of both PatientResponseInfo and PhenotypeResponseInfo.

    :param ModelParameters model_parameters: the parameters of the
model that
        includes this patient
    :param str parameter_name: the name of the model parameter by
which patient
        contribution and clinician contribution should be combined.
The named
        parameter must either have a value which is a function name
(missing
        an underscore, e.g. 'heavily_patient') or a value which is a
float
        between zero and one.  Zero is entirely clinician; one is
entirely
        patient.
    """
    def __init__(self, model_parameters, parameter_name):
        self._model_parameters = model_parameters
        self._parameter_name = parameter_name

    def aggregate(self, patient):
        """What is combination of patient and clinician, as qty in [0,
1]?"""
        param_value =  getattr(self._model_parameters,
self._parameter_name)
        clinician = patient.clinician
        if isinstance(param_value, Number):
```

```
            return self._linear_tilted(
                patient, clinician, np.clip(param_value, 0.0, 1.0)
            )
        elif param_value is None:
            return 0
        else:
            return getattr(self, '_' + param_value)(patient,
clinician)

    def _only_participation_counts(self, patient, _):
        """Return aggregation based only on patient patricipation."""
        return 1 if patient.at_least('participating') else 0

    def _patient_only(self, patient, clinician):
        """Ignore clinician engagement."""
        return patient.engagement()

    def _heavily_patient(self, patient, clinician):
        """"Linear aggregation, weighted heavily toward patient."""
        return self._linear_tilted(patient, clinician, 0.8)

    def _slightly_patient(self, patient, clinician):
        """Linear aggregation, weighted slightly toward patient."""
        return self._linear_tilted(patient, clinician, 0.6)

    def _even_both(self, patient, clinician):
        """Return simple linear function of patient and clinician
engagement."""
        return self._linear_tilted(patient, clinician, 0.5)

    def _slightly_clinician(self, patient, clinician):
        """Linear aggregation, weighted slightly toward clinician."""
        return self._linear_tilted(patient, clinician, 0.4)

    def _heavily_clinician(self, patient, clinician):
        """Linear aggregation, weighted heavily toward clinician."""
        return self._linear_tilted(patient, clinician, 0.2)

    def _clinician_only(self, patient, clinician):
        """Ignore patient engagement."""
        return clinician.engagement()

    def _linear_once_participating(self, patient, clinician):
        """Return even both, but with aware = 0."""
        return (
            patient.participatory_engagement() +
            clinician.participatory_engagement()
        ) / 2.0

    def _linear_tilted(self, patient, clinician, lean):
```

```
        """Return linear aggregation, learning to patient or
clinician."""
        return patient.engagement() * lean + clinician.engagement() *
(1 - lean)

#
# Phenotypes and treatment packages
#

class Phenotype:
    """Some biological variant of a disease or a patient.

    :param int phenotype_ID: unique integer identifier of phenotype
    """
    def __init__(self, phenotype_ID):
        self._id = phenotype_ID

    def __str__(self):      # pragma: no cover
        return 'Phenotype({})'.format(self._id)


class TreatmentPackage:
    """A particular package of treatments to treat a condition.

    A clinician will prescribe a particular treatment package for a
patient,
    and may prescribe a different treatment package if the first one
is not
    successful.

    :param int treatment_package_id: unique integer identifier of
treatment
        package, typically generated in sequence
    :param phenotypes: the list of all phenotypes in this model
    :type phenotypes: list of strings
    :param float effect_mean: mean effect of treatment package on
patient.
        The effect is measured in change in outcome per week. For all
patients
        of a particular phenotype, the effect of a treatment package
will be
        the same. The mean is across the phenotypes.
    :param float effect_sd: standard deviation of effect of treatment
package
        on patient. The effect is measured in change in outcome per
week.
        For all patients of a particular phenotype, the effect of a
treatment
        package will be the same. The standard deviation is across the
        phenotypes.
```

```
    """
    def __init__(self, treatment_package_id, phenotypes,
effect_mean,effect_sd):
        self._id = treatment_package_id
        self._effect_mean = effect_mean
        self._effect_sd = effect_sd
        self._setup_outcome_improvement(phenotypes)

    def __str__(self):       # pragma: no cover
        return 'TreatmentPackage({})'.format(self._id)

    def _setup_outcome_improvement(self, phenotypes):
        """Create a mapping from phenotype to change in outcome for
the TP."""
        self._outcome_improvement = {
            phenotype: self._draw_from_effect_distribution()
            for phenotype in phenotypes}

    def _draw_from_effect_distribution(self):
        """Return a single draw from a treatment package effect
distribution."""
        return np.random.normal(self._effect_mean, self._effect_sd)

    def outcome_improvement(self, phenotype):
        """For this phenotype, what is the weekly improvement in
outcome?

        The improvement can be negative, i.e. the treatment package
may make
        the condition worse, for this phenotype.

        :param Phenotype phenotype: the phenotype that this treatment
package
            could be applied to
        :returns: improvement, measured in change in outcome per week
        :rtype: float
        """
        return self._outcome_improvement[phenotype]


class MedicalRecord:
    """The record of a patient's outcomes, over time.

    Note that this class serves a very different purpose
from :class:`Trace`.
    This class models the improvement (or failure to
    improve) in the real world that is modeled. :class:`Trace`
captures
    everything that happens in the simulation model, for reporting and
    analysis. :class:`MedicalRecord` models the world; :class:`Trace`
```

```
models
    the model.
    """
    def __init__(self):
        self._records = []

    def update_records(self, perceived_outcome, time):
        """Add the latest outcome to the medical record.

        Records are updated during a clinical encounter.

        :param float perceived_outcome: the current outcome of the
patient, as
            perceived by the clinician
        :param int time: the current week
        """
        self._records.append({
            'time': time,
            'perceived_outcome': perceived_outcome
        })

    def is_patient_improving(self):
        """Is the patient's outcome improving?

        Based on the records we have captured, is the patient's
outcome
        improving? Improving is defined as either a greater outcome
than
        the last encounter, or the same outcome but greater than 0.95.
        (Other definitions of improving are possible, of course.)

        :rtype: boolean
        """
        # Simple determination for now: latest outcome > outcome
before
        if len(self._records) < 2:
            raise LearningNetworkModelException(
                'Cannot tell if patient is improving, from {}
records'.format(
                    len(self._records)))
        else:
            outcome_now = self._records[-1]['perceived_outcome']
            outcome_before = self._records[-2]['perceived_outcome']
            return (
                outcome_now > outcome_before or
                (outcome_now == outcome_before and outcome_now >
0.95))

#
# Will a patient transition? How much does an agent contribute to
```

```
shared
# knowledge?
#

class TransitionDeterminer:
    """Determines whether an agent will transition to a new state now.

    Two instances of this class are supplied
to :class:`LearningNetworkModel`,
    one for patient activation and one for patient dispirit. The
activation
    instance determines at simulation time whether a patient
activates, and
    the dispirit instance performs the same function for the dispirit
    transition.

    :param triples: a list of triples, each of which specifies the
probability
        of transition in one clinical encounter situation. The
situation
        includes three components: the state of the patient at the
time
        of the clinical encounter, the state of the clinician at the
same
        time, and the probability of transition. For example, if one
triple
        in the list is ('aware', 'owning', 0.5), that means that if a
patient
        is aware and has a clinical encounter with a clinician who is
owning,
        there is a 50% chance that the patient will transition (i.e.
activate
        and become participating) because of the encounter.
    :type triples: list of three-element tuples
    :param dict: a nested dictionary. Each key in the dict is a
patient state.
        The value of a dict element is another dictionary, or
clinician states
        and probabilities of transition. For example, a dict of
        {'aware': {'owning': 0.5}} means that if a patient is aware
and has a
        clinical encounter with
        a clinician who is owning, there is a 50% chance that the
patient will
        transition (i.e. activate and become participating) because
the
        encounter. If **dict** is specified, **triples** is ignored.
    :type dict: dictionary keys that are two-element tuples
    """
    def __init__(self, *triples, dict=None):
```

```
        self._probabilities = collections.defaultdict(float)
        if dict:
            for patient_state, pvals in dict.items():
                for clinician_state, prob in pvals.items():
                    self._probabilities[patient_state,
clinician_state] = prob
        else:
            for patient_state, clinician_state, prob in triples:
                self._probabilities[(patient_state, clinician_state)]
= prob

    @classmethod
    def default_activate(cls):
        """A default specification for activate transitions.

        The default is used when a :class:`LearningNetworkModel`
        instance is created without specifying some other transition
        determiner for activate.

        :returns: a good default for activate transitions
        :rtype: TransitionDeterminer
        """
        return TransitionDeterminer(
            ('aware', 'participating', 0.05),
            ('aware', 'contributing', 0.1),
            ('aware', 'owning', 0.2),
            ('participating', 'contributing', 0.05),
            ('participating', 'owning', 0.1),
            ('contributing', 'owning', 0.05))

    @classmethod
    def default_dispirit(cls):
        """A default specification for dispirit transitions.

        The default is used when a :class:`LearningNetworkModel`
        instance is created without specifying some other transition
        determiner for dispirit.

        :returns: a good default for dispirit transitions
        :rtype: TransitionDeterminer
        """
        return TransitionDeterminer(
            ('participating', 'aware', 0.05),
            ('contributing', 'aware', 0.1),
            ('contributing', 'participating', 0.05),
            ('owning', 'aware', 0.2),
            ('owning', 'participating', 0.1),
            ('owning', 'contributing', 0.05))

    @classmethod
```

```
    def activate_specified_numerically(klass, value):
        """A default based only on value for clinician owning, patient
aware"""
        return TransitionDeterminer(
            ("aware", "participating", value / 4.0),
            ("aware", "contributing", value/2.0),
            ("aware", "owning", value),
            ("participating", "contributing", value/4.0),
            ("participating", "owning", value/2.0),
            ("contributing", "owning", value/4.0)
        )

    @classmethod
    def dispirit_specified_numerically(klass, value):
        """A default based only on value for clinician aware, patient
owning"""
        return TransitionDeterminer(
            ("owning", "aware", value),
            ("owning", "participating", value/2.0),
            ("owning", "contributing", value/4.0),
            ("contributing", "aware", value/2.0),
            ("contributing", "participating", value/4.0),
            ("participating", "aware", value/4.0)
        )

    def does_transition_now(self, patient):
        """Does the patient transition now?

        :param Patient patient: the patient who might transition to a
new
            state

        :rtype: boolean
        """
        return np.random.random() < self.probability(
            patient.state, patient.clinician.state)

    def probability(self, patient_state, clinician_state):
        """Return the probability of transition.

        :param str patient_state: the state of a patient
        :param str clinician_state: the state of a clinician
        :returns: the probability that the patient will transition
            during an encounter, given the states
        :rtype: float
        """
        return self._probabilities[(patient_state, clinician_state)]

    def print(self, f):
        """Print the probabilities in a table.
```

```
        :param f: the file-like object to print to, e.g. created with
open()
        :type f: file-like object
        """
        print('    patient state     clinician state     probability',
file=f)
        print('    -------------     ---------------     -----------',
file=f)
        for states, probability in self._probabilities.items():
            print('    {:<13}     {:<15}     {:>11}'.format(
                states[0], states[1], probability), file=f)

    def as_list(self):
        """Return the probabilities as a list of dicts"""
        return [
            {
                "patient_state": states[0],
                "clinician_state": states[1],
                "probability": probability
            }
            for states, probability in self._probabilities.items()
            if probability > 0  # correcting for defaultdict
        ]


class ContributionDeterminer:
    """Determines whether and how much agent will contribute shared
knowledge.

    Patients and clinicians periodically contribute to shared
knowledge.
    Do they contribute now? And if they contribute, how much are they
    contributing?

    Two instances of this class are supplied
to :class:`LearningNetworkModel`,
    one to specify shared knowledge contributions from patients, the
other
    to specify shared knowledge contributions from clinicians.

    :param dict states_periods_and_amounts: a dict with keys of agent
states.
        Each value in the dict is a two-element tuple or list. The
first
        element is
        the (average) period between shared knowledge contributions.
The second
        element is the amount of the shared knowledge contribution
when it
```

```
        occurs. For example, if states_periods_and_amounts is
        {'owning': (40, 1)}, that means that the agent only contribute
        when he is owning (and not any other state), he contributes
every
        40 weeks (on average) and contributes 1.0 units of shared
knowledge
        when he makes a contribution.
    """
    def __init__(self, states_periods_and_amounts):
        self._states_periods_and_amounts = states_periods_and_amounts

    @classmethod
    def default_for_patients(cls):
        """A default specification for patient contribution to shared
knowledge.

        The default is used when a :class:`LearningNetworkModel`
        instance is created without specifying some other contribution
        specification for patients.

        :returns: a good default for patient shared knowledge
contributions
        :rtype: Contribution Determiner
        """
        return ContributionDeterminer({
            'participating': (40, 0.5),
            'contributing': (20, 1),
            'owning': (20, 1.5)
        })

    @classmethod
    def specified_numerically(klass, value):
        """A default based only on a single numeric value"""
        return ContributionDeterminer({
            'participating': (4*value, 1),
            'contributing': (2*value, 1),
            'owning': (value, 1)
        })

    @classmethod
    def default_for_clinicians(cls):
        """A default specification for clinician contribution to shd
knowledge.

        The default is used when a :class:`LearningNetworkModel`
        instance is created without specifying some other contribution
        specification for clinicians.

        :returns: a good default for clinician shared knowledge
contributions
```

```
        :rtype: Contribution Determiner
        """
        return ContributionDeterminer({
            'participating': (20, 0.5),
            'contributing': (10, 1),
            'owning': (10, 1.5)
        })

    def does_contribute_now(self, agent):
        """Does the agent contribute to shared knowledge this week?

        :param LearningNetworkAgent agent: the patient or clinician
who might
            contribute shared knowledge this simulated week
        :rtype: boolean
        """
        try:
            period, amount =
self._states_periods_and_amounts[agent.state]
            if period > 0.0:
                return np.random.random() < 1.0 / period
            else:
                return False
        except KeyError:
            return False

    def contribution_amount(self, agent):
        """How much does agent contribute to shared knowledge now?

        Given that the patient or clinician is contributing to shared
        knowledge this simulated week, how much does he contribute?

        :param LearningNetworkAgent agent: the patient or clinician
who
            contributes to shared knowledge this simulated week

        :returns: amount of shared knowledge contributed
        :rtype: float
        """
        try:
            period, amount =
self._states_periods_and_amounts[agent.state]
            return amount
        except KeyError:
            return 0.0

    def print(self, f):
        """Print the periods and amounts in a table.

        :param f: the file-like object to print to, e.g. created with
```

```
open()
        :type f: file-like object
        """
        print('    agent state       avg period     amount', file=f)
        print('    -----------       ----------     ------', file=f)
        for state, results in
self._states_periods_and_amounts.items():
            print('    {:<13}     {:>10}     {:>6}'.format(
                state, results[0], results[1]), file=f)

    def as_list(self):
        """Return the amounts as a list of dicts"""
        return [
            {
                "agent_state": state,
                "average_period": results[0],
                "amount": results[1],
            }
            for state, results in
self._states_periods_and_amounts.items()
        ]

#
# LearningNetworkModel: the model as a whole
#

class LearningNetworkModel(mesa.Model):
    """A model of a learning network.

    There are many parameters, all with default values. A typical run
    of the model need only specify some of these parameters. The
parameters
    themselves are defined in the class :class:`ModelParameters`.

    Some parameters enable (or disable) some model logic. For example
the
    parameter **cohort_exit_on** enables the open cohorts model logic
of
    patients exiting the cohort during the simulation. When
**cohort_exit_on**
    is True, patients will exit; when False, no patient will exit the
cohort.
    All the parameters that enable (or disable) model logic are named
    with the suffix **-on**. All these -on parameters are False by
default.

    Some parameters refer to *praxis*. Praxis is how much is known
about a
    patient, for the purpose of making a treatment decision, and is
measured
```

on a scale [0.0, 1.0].

Some parameters refer to *selection efficiency*, how well a clinician selects a treatment for her patient. For a particular clinician, selection efficiency will differ from patient to patient, based on the praxis for that patient.

A greater selection efficiency means that the clinician is more likely to select an effective treatment package for the patient, and less likely to select an ineffective treatment package. The minimum possible selection efficiency is 0.0, which means even odds: the clinician is just as likely to select an ineffective (or countereffective) treatment package as an effective one.

If there are two treatment packages with effectiveness F1 and F2, and F1 is more effective than F2 by a difference in effectiveness of d, and if selection efficiency is 1.0, then the odds of selecting F1 instead of F2 is (1 + d). If selection efficiency is 2.0, then the odds are (1 + d)^2. In fact the odds increase by a factor of (1 + d) for each additional unit of selection efficiency.

There is some minimum selection efficiency if a clinician knows nothing about a patient, e.g. he is a new patient in the clinician's solo practice. There is a maximum selection efficiency, the selection efficiency if the clinician has a praxis of 1.0 for this patient.

The selection efficiency for a patient with some praxis in between 0.0 and 1.0 is modeled as a linear function of the praxis, from the minimum selection efficiency to the maximum.

:param care_centers: specification of the patient count and clinician count in each care center. A list, with one element for each care center. Each element is a dict, with mandatory keys patient_count and clinician_count, each of whose values is an integer. Optional keys include previsit_planning (either 'high', 'medium', or 'low', with default 'low'), enhanced_registry (either 'no contribution', 'contribution', 'contribution and real-time usage', with default

```
        'no contribution'). Alternative parameterization: count of the
        care centers, each with 200 patients and 10 clinicians,
rounding float
        to int if needed.
        Default: [{'patient_count': 200, 'clinician_count': 10}]
    :type care_centers: list of dict, or int or float
    :param str previsit_planning: if the care centers are specified
with a
        simple count (i.e. if **care_centers** is a number rather than
a list),
        what is the level of previsit planning for each of the care
        centers? Must be either 'low', 'medium', or 'high'. Ignored if
care
        centers are not specified with a count. Default: 'low'
    :param str enhanced_registry: if the care centers are specified
with a
        simple count (i.e. if **care_centers** is a number rather than
a list),
        what is the degree of enhanced registry for each of the care
        centers? Must be either 'no contribution', 'contribution', or
        'contribution and real-time usage'. Ignored if care
        centers are not specified with a count. Default: 'no
contribution'
    :param boolean patients_gradual_on: are patients in the initial
cohort
        added gradually (True) or all at once (False)? Default: False
    :param int patients_gradual_duration: if patients are added
gradually (i.e.
        **patient_gradually_on** is True), over how many weeks are all
the
        initial patients added? Default: 13
    :param boolean cohort_entrance_on: do patients enter the cohort
later, e.g.
        because they develop the condition?  Default: False
    :param float cohort_entrance_per_month: if patients enter the
cohort later
        (i.e. **cohort_entrance_on** is True) how many patients are
added every
        month, on average? Note that patients enter weekly, but the
amount is
        specified monthly. Note also that the patient arrival is a
poisson
        distribution, with a mean of the specified monthly entrance.
Default:
        10
    :param boolean cohort_exit_on: do patients leave the cohort, e.g.
because
        they move away? Default: False
    :param float age_out_annual_proportion: if patients leave the
cohort (i.e.
```

```
        **cohort_exit_on** is True), what proportion of the patients
age out
        each year? Note that patients may age out each week, but the
        probability is specified annually. Default: 0.1
    :param float move_away_annual_proportion: if patients leave the
cohort
        (i.e. **cohort_exit_on** is True), what proportion of the
patients
        move away or otherwise leave the cohort for exogenous reasons
unrelated
        to their age or medical condition? Note that patients may move
away
        each week, but the probability is specified annually. Default:
0.05
    :param float get_better_annual_proportion: if patients leave the
cohort
        (i.e. **cohort_exit_on** is True), what proportion of the
patients
        who have an outcome of 1.0 (the max) leave the cohort each
year
        because they are better? Note that patients may leave on any
week
        because they are better, but the probability is specified
annually.
        Default: 0.5
    :param float get_better_start_outcome: if patients leave the
cohort (i.e.
        **cohort_exit_on** is True), above what outcome level might a
patient
        leave the cohort because he is better? The probability of exit
is a
        linear function of the outcome, from 0.0 at this
        **get_better_start_outcome** level to
**get_better_annual_proportion**
        at outcome level of 1.0. Default: 0.9
    :param float get_worse_annual_proportion: if patients leave the
cohort
        (i.e. **cohort_exit_on** is True), what proportion of the
patients
        who have an outcome of 0.0 (the min) leave the cohort each
year because
        their outcome is poor? Note that patients may leave because
they are
        better on any week, but the probability is specified annually.
Default:
        0.8
    :param float get_worse_end_outcome: if patients leave the cohort
(i.e.
        **cohort_exit_on** is True), below what outcome level might a
patient
```

```
        leave the cohort because his outcome is poor? The probability
of exit
        is a linear function of the outcome, from
        **get_worse_annual_proportion** at an outcome level of 0.0 to
0.0 at
        an outcome level of **get_worse_end_outcome**. Default: 0.1
    :param boolean replace_departing_patients_on: when a patient
leaves the
        cohort for any reasons, is he replaced by a new patient who
fills the
        resulting open spot? Default: False
    :param int phenotype_count: how many distinct patients phenotypes
exist?
        The treatment packages give different outcomes for each
phenotype.
        Default: 4
    :param initial_outcome_distr: the probability distribution of the
        initial patient outcomes. Valid alternatives:

            * 'uniform_0_1': uniform distribution between 0.0 and 1.0
            * 'uniform_08_1': uniform distribution between 0.8 and 1.0
            * 'uniform_0_03': uniform distribution between 0.0 and 0.3
            * 'beta_2_5': a beta distribution with alpha=2 and beta=5.
That distribution has a mode of 0.2 and a median of roughly 0.26
            * 'beta_3_4': a beta distribution with alpha=3 and beta=4
            * 'beta_3-5_3-5': a beta distribution with alpha and beta
both 3.5
            * 'beta_4_3': a beta distribution with alpha=4 and beta=3
            * 'beta_5_2': a beta distribution with alpha-5 and beta=2
            * a list of floats (e.g. [0.0, 0.4, 0.6]), indicating that
outcomes should cycle through the list, one patient at a time.
        Default: beta_2_
    :type initial_outcome_distr: str or list of floats
    :param boolean random_walk_on: does a patient's outcome vary from
week to
        week? Default: False
    :param float random_walk_mean: if a patient's outcome varies from
week
        to week (i.e. **random_walk_on** is True), what is the mean of
that
        weekly variance? Default 0.0
    :param float random_walk_sd: if a patient's outcome varies from
week to
        week (i.e. **random_walk_on** is True), what is the standard
deviation
        of that weekly variance? Default 0.01

    :param boolean relapse_on: might a patient relapse, i.e. see a
dramatic
        reduction in outcome in a single week? Default: False
```

```
    :param float relapse_period: if a patient might relapse (i.e.
        **relapse_on** is True), how many weeks (on average) between
relapses?
        Note that the relapse events do not recur on any regular
schedule.
        Instead **relapse_period** serves to define a probability of
relapse
        each week. Default: 52
    :param float relapse_amount: if a patient might relapse (i.e.
        **relapse_on** is True), how much outcome will he lose when he
        relapses? For example, if his outcome is 0.8,
**relapse_amount** is
        0.5, and he relapses this week, his outcome will fall to 0.3.
Default:
        0.5

    :param boolean treatment_package_on: might a patient's outcome be
affected
        because of the application of a treatment package? Default:
False
    :param int treatment_package_count: how many distinct treatment
packages
        could be used to treat this condition? Default: 8
    :param float treatment_package_effect_mean: if a patient's outcome
might
        be affected by the application of a treatment package (i.e.
        **treatment_package_on** is True), what is the mean weekly
improvement
        in outcome of a treatment package? Note that a treatment
package is
        modeled as having the same effect for each patient of a
particular
        phenotype, so **treatment_package_effect_mean** is the mean
effect
        across the phenotypes, not across the patients. Note that if
both
        **best_treatment_package_effect** and
**worst_treatment_package_effect**
        are specified, any value for this parameter is ignored, and
the
        mean is instead estimated from the best, worst, and
        **treatment_package_count**. Default: 0.0
    :param float treatment_package_effect_sd: if a patient's outcome
might be
        affected by the application of a treatment package (i.e.
        **treatment_package_on** is True), what is the standard
deviation of
        the weekly improvement in outcome of a treatment package? Note
that
        a treatment package is modeled as having the same effect for
```

```
each
        patient of a particular phenotype, so
**treatment_package_effect_sd**
        is the effect standard deviation across the phenotypes, not
across
        the patients. Note that if both
        **best_treatment_package_effect** and
**worst_treatment_package_effect**
        are specified, any value for this parameter is ignored, and
the
        SD is instead estimated from the best, worst, and
        **treatment_package_count**. Default: 0.02
    :param best_treatment_package_effect: what is the expected value
        of the weekly improvement of the best treatment package for a
particular
        phenotype? This is an alternate (and easier) way to estimate
        **treatment_package_effect_mean** and
**treatment_package_effect_sd**.
        Default: None
    :type best_treatment_package_effect: float or None
    :param worst_treatment_package_effect: what is the expected value
        of the weekly improvement of the worst treatment package for a
        particular phenotype? This is an alternate (and easier) way to
estimate
        **treatment_package_effect_mean** and
**treatment_package_effect_sd**.
        Default: None
    :type worst_treatment_package_effect: float or None

    :param str clinician_assignment: how are clinicians assigned to
patients?
        Valid alternatives:

            * 'random': each patient is assigned a clinician randomly
from the set of clinicians.
            * 'successive': the first patient is assigned the first
clinician, the second patient is assigned the second clinician, and so
on. After the last clinician is assigned, the first clinician is
assigned again, to the next patient, repeating in a cycle.

        Default: 'random'
    :param encounter_period: after how many weeks does a patient have
a
        clinical encounter with a clinician? Valid options:

            * an integer: all patients have the same encounter period.
            * None: each patient has a different encounter period,
selected randomly, of at least 10 weeks, and no more than 16 weeks.

        Default: 13
```

```
    :type encounter_period: int or None

    :param patient_states: what initial state is assigned to each
patient?
        Valid options:

            * 'all_unaware': each patient is initially unaware
            * 'all_aware': each patient is initially aware
            * 'all': first patient is unaware, the second is aware,
third is participating, fourth contributing, fifth owning, then the
sixth is aware again, and so on, in a cycle
            * 'all_except_unware': first patient is aware, second is
participating, third contributing, fourth owning, then the fifth is
aware again, and so on, in a cycle
            * 'random_choice': each patient is assigned an initial
state among the five possible states, randomly
            * list of states: the first patient is assigned the first
state in the list, the second patient is assigned the second state in
the list, and so on, cycling as needed
            * a dictionary of engagement states and proportions

        Default: 'all_unaware'
    :type patient_states: str or list of states (as strings) or dict
of states and proportions
    :param clinician_states: what initial state is assigned to each
clinician?
        Valid options:

            * 'all_unaware': each clinician is initially unaware
            * 'all_aware': each clinician is initially aware
            * 'all': first clinician is unaware, the second is aware,
third is participating, fourth contributing, fifth owning, then the
sixth is aware again, and so on, in a cycle
            * 'all_except_unware': first clinician is aware, second is
participating, third contributing, fourth owning, then the fifth is
aware again, and so on, in a cycle
            * 'random_choice': each clinician is assigned an initial
state among the five possible states, randomly
            * list of states: the first clinician is assigned the
first state in the list, the second clinician is assigned the second
state in the list, and so on, cycling as needed
            * a dictionary of engagement states and proportions

        Default: 'all_aware'
    :type clinician_states: str or list of states (as strings) or dict
of states and proportions

    :param boolean encounter_aware_on: might a clinical encounter
between an
        unaware patient and a clinician who is at least aware lead to
```

```
the
        patient becoming aware? Default: False
    :param float encounter_aware_likelihood: if a clinical encounter
might lead
        to a patient becoming aware (i.e. **encounter_aware_on** is
True),
        what is the probability of an unaware patient becoming aware,
during
        a clinical encounter with a clinician who is at least aware?
Default:
        0.5

    :param boolean encounter_activate_on: might a clinical encounter
between a
        patient and a clinician cause the patient to increase
        activation? Default: False
    :param patient_activate_determiner: if a clinical
        encounter might cause a patient to increase activation (i.e.
        **encounter_activate_on** is True), what is the probability of
an
        increased activation? Could be provided as either a
        :class:`TransitionDeterminer`
        object, or as a nested dictionary, or as a float. If provided
as
        a dictionary, the
        key of each element in the dictionary is a patient
        state. The value of an element is a dict of clinician states
and
        probabilities of increased activation. If provided as a single
        float, the probability of activate for an owning clinician and
        an aware patient is provided, and others calculated from that
value.
        Default: TransitionDeterminer.default_activate()
    :type patient_activate_determiner: TransitionDeterminer or
dict[str, dict[str, float] ] or float
    :param boolean encounter_dispirt_on: might a clinical encounter
between
        a patient and a clinician cause the patient to lower
activation?
        Default: False
    :param patient_dispirit_determiner: if a clinical
        encounter might cause a patient to lower activation (i.e.
        **encounter_dispirit_on** is True), what is the probability of
        a lowered activation? Could be provided as either a
        :class:`TransitionDeterminer` object, or as a nested
dictionary, or as
        a float. If provided as a dictionary, the
        key of each element in the dictionary is a patient
        state. The value of an element is a dict of clinician states
and
```

```
        probabilities of decreased activation. If provided as a single
float,
        the probabilitty of dispirit for an aware clinician and an
owning
        patient is provided, and others calculated from that value.
Default:
        TransitionDeterminer.default_dispirit()
    :type param_dispirit_determiner: TransitionDeterminer or dict[str,
dict[str, float] ] or float

    :param boolean patient_influence_on: might a patient change its
state
        because of the influence of another patient? Default: False
    :param float patient_network_edges: how many other patients
influence
        a patient, as an average across all patients? Note that
influences
        are undirected; if patient Paul influences patient Patrick,
then
        Patrick also influences Paul. Default: 0.0
    :param float patient_influence_across_prop: what proportion of
influence
        links between patients cross care center boundaries? Default:
0.0, a
        sensible default in the default case of only a single care
center
    :param float patient_influence_become_aware_probability: if a
patient
        can be influenced by other patients (i.e.
**patient_influence_on** is
        True), and if a particular patient is unaware and one of its
        influencers is at least aware, what is the annual probability
that
        the unaware patient will become aware because of the
influence? Note
        the state transition may occur on any week, but the
probability
        is specified annually. Default: 0.1
    :param float patient_influence_activation_probability: if a
patient
        can be influenced by other patients (i.e.
**patient_influence_on** is
        True), and if a particular patient has an influencer that is
of
        greater activation, what is the annual probability that
        the more highly activated influencer will cause the less
activated
        patient to become more activated, because of the influence?
Note
        the state transition may occur on any week, but the
```

```
probability
        is specified annually. Default: 0.1

    :param boolean clinician_influence_on: might a clinician change
its
        state because of the influence of another clinician? Default:
False
    :param float clinician_network_edges: how many other clinicians
influence
        a clinician, as an average across all clinicians? Note that
influences
        are undirected; if clinician Claire influences clinician
Claudia,
        then Claudia also influences Claire. Default: 0.0
    :param float clinician_influence_across_prop: what proportion of
influence
        links between clinicians cross care center boundaries?
Default: 0.0, a
        sensible default in the default case of only a single care
center.
    :param float clinician_influence_become_aware_probability: if a
clinician
        can be
        influenced by another clinician (i.e.
**clinician_influence_on** is
        True), and if a particular clinician is unaware and one of its
        influencers is at least aware, what is the annual probability
that
        the unaware clinician will become aware because of the
influence?
        Note the state transition may occur on any week, but the
probability
        is specified annually. Default: 0.8
    :param float clinician_influence_activation_probability: if a
clinician
        can be influenced by another clinician (i.e.
        **clinician_influence_on** is
        True), and if a particular clinician has an influencer that is
of
        greater activation, what is the annual probability that
        the more highly activated influencer will cause the less
activated
        clinician to become more activated, because of the influence?
Note
        the state transition may occur on any week, but the
probability
        is specified annually. Default: 0.5

    :param boolean clinician_dispirit_on: might a clinician become
dispirited,
```

```
        and decline in activation state? Default: False
    :param float clinician_dispirit_probability: what is the annual
probability
        that a clinician who is at least participating will dispirit
to a
        lower activation state?
        Note the state transition may occur on any week, but the
probability
        is specified annually. Default: 0.1

    :param float shared_knowledge_initial: how much shared knowledge
is
        available in the learning network initially, as a commons
across
        all agents? Shared knowledge is measured in units of
contributions.
        When a contributing patient contributes to shared knowledge,
he
        contributes 1 unit (by default). Default: 0.0
    :param boolean shared_knowledge_decays_on: does the amount of
shared
        knowledge decline over time? Shared knowledge might decline
for a
        variety of reasons: it becomes out of date with the latest
        practice, software no longer works, books are
        lost, etc. Default: False
    :param float shared_knowlege_half_life: if shared knowledge
declines
        over time (i.e. **shared_knowledge_decays_on**), and if there
are no
        new contributions to shared knowledge, how many weeks until
the
        amount of shared knowledge has declined by half? Note that the
        decay of shared knowledge occurs whether new contributions are
        made or not, but it is more convenient to frame the half-life
        measure using the situation of no new contributions. Default:
153
        (weeks)

    :param boolean patient_contributes_shared_knowledge_on: might a
sufficiently
        engaged patient contribute shared knowledge to the learning
network
        commons? Default: False
    :param patient_shared_knowledge_contrib_determiner:
        if a patient might contribute shared knowledge (i.e.
        **patient_contributes_shared_knowledge_on** is True), how
often will he
        contribute? And how much will that contribution increase the
quantity
```

```
        of shared knowledge? If expressed as a dictionary instead of
an
        instance of :class:`ContributionDeterminer`, the key of each
dict is a
        patient activation state (e.g. 'participating'), and the value
of the
        dict is a tuple or list of the average period between shared
knowledge
        contributions (in weeks), and the amount of shared knowledge
        contribution when it occurs. Alternate parameterization: an
int or
        float for the owning period, with an assumption the
contributing period
        is twice the owning, and the participating period is twice the
        contributing, and with one item with each contribution.
        Default:
        :class:`ContributionDeterminer`.default_for_patients()
    :type patient_shared_knowledge_contrib_determiner:
ContributionDeterminer or dict[str, (float, float)] or int or float

    :param boolean clinician_contributes_shared_knowledge_on: might a
        sufficiently engaged clinician contribute shared knowledge to
the
        learning network commons? Default: False
    :param clinician_shared_knowledge_contrib_determiner:
        if a clinician might contribute shared knowledge (i.e.
        **clinician_contributes_shared_knowledge** is True), how often
will she
        contribute? And how much will that contribution increase the
quantity
        of shared knowledge? If expressed as a dictionary instead of
an
        instance of :class:`ContributionDeterminer`, the key of each
dict is a
        clinician activation state (e.g. 'participating'), and the
value of the
        dict is a tuple or list of the average period between shared
knowledge
        contributions (in weeks), and the amount of shared knowledge
        contribution when it occurs. Alternate parameterization: an
int or
        float for the owning period, with an assumption the
contributing period
        is twice the owning, and the participating period is twice the
        contributing, and with one item with each contribution.
Default:
        :class:`ContributionDeterminer`.default_for_clinicians()
    :type patient_shared_knowledge_contrib_determiner:
ContributionDeterminer or dict[str, (float, float)] or int or float
```

```
    :param int enhanced_registry_initial: how many records are in the
        enhanced registry at the beginning of the simulation? Each
record
        is a single clinical encounter. Default: 0.
    :param enhanced_registry_initial_per_patient: how many records are
in the
        enhanced registry at the beginning of the simulation,
expressed per
        patient, as an alternative parameterization of
        **enhanced_registry_initial**. If not None, the value of
        **enhanced_registry_initial** is ignored. Default: None,
meaning use
        the value of **enhanced_registry_initial** instead.
    :type enhanced_registry_initial_per_patient: int or None
    :param boolean enhanced_registry_affects_commons_on: does the
enhanced
        registry increase the commons, due to periodic analysis of the
        records in the enhanced registry? Default: False
    :param int enhanced_registry_analysis_period: how often does is
the
        enhanced registry analyzed, measured in weeks between
analyses?
        Default: 13
    :param int enhanced_registry_records_per_commons_item: when the
enhanced
        registry is analyzed, how many records in the enhanced
registry does
        it take to create a single item in the commons? Default: 1000

    :param boolean phenotype_response_info_affects_praxis_on: does the
        phenotype response information affect praxis? Default: False
    :param float potential_phenotype_response_info_from_SK_unit: how
much
        does a single unit of shared knowledge increase the phenotype
        response info, assuming there is sufficient patient and
clinician
        engagement? Default: 0.001
    :param str phenotype_realization: how does
        patient engagement and clinician engagement affect the
translation
        of shared knowledge to phenotype response info? In all
situations,
        an unaware patient and an unaware clinician combine to
translate
        any amount of shared knowledge to zero phenotype response
info.
        And an owning patient and an owning clinician
        combine to translate all shared knowledge into praxis,
according to
        factor **potential_phenotype_response_info_from_SK_unit**. But
```

```
        intermediate engagements are translated differently. Valid
alternatives:

            * 'only_participation_counts': if patient is at least
participating, the full extent of increase is realized. Otherwise
nothing. Clinician engagement is ignored.
            * 'patient_only': the progress of patient engagement only.
Clinician engagement is ignored.
            * 'heavily_patient': both progress of patient engagement
and progress of clinician engagement are considered, as a weighted
average, heavily weighted toward patients
            * 'slightly_patient': both progress of patient engagement
and progress of clinician engagement are considered, as a weighted
average, slightly weighted toward patient progress
            * 'even_both': both progress of patient engagement and
progress of clinician engagement are considered, and evenly weighted.
            * 'slightly_clinician': both progress of patient
engagement and progress of clinician engagement are considered, as a
weighted average, slightly weighted toward clinician progress
            * 'heavily_clinician': both progress of patient engagement
and progress of clinician engagement are considered, as a weighted
average, heavily weighted toward clinician
            * 'clinician_only': the progress of clinician engagement
only. Patient engagement is ignored.
            * 'linear_once_participating': the mean progress of
patient engagement and clinician engagement, but with no progress
noted for agents who are only aware

        Default: 'linear_once_participating'

    :param phenotype_realization_numeric: how does
        patient engagement and clinician engagement affect the
translation
        of shared knowledge to phenotype response info? This parameter
is a
        numeric alternative to **phenotype_realization**, expressing
the mix
        of clinician engagement and patient engagement that is used. A
value
        of zero is the same as 'clinician_only'. A value of one is the
same as
        'patient_only'. A value of 0.5 is the same as 'even_both'. A
non-None
        value is used instead of **phenotype_realization**. None
indicates to
        use **phenothype_realization**. Default: None
    :type phenotype_realization_numeric: float or None

    :param boolean patient_response_info_affects_praxis_on: does the
patient
```

```
        response information affect praxis? Default: False

    :param float patient_response_info_initial: for each particular
patient,
        what is the initial level of patient response info? Default:
0.0

    :param float patient_response_info_half_life: if a patient is
unaware and
        his clinician is also unaware, no additional patient response
info will
        be added. In that situation, how many weeks until the level of
        patient response info has declined by half? Note that the
decay of
        patient response info occurs whether or not there is new
patient
        response info added, but it is convenient to frame the half-
life
        measure using the situation of no new contribution. Default:
20 (weeks)

    :param str patient_response_info_increase: how does patient
engagement and
        clinician engagement combine to affect the increase in patient
        response info? In all situations, an unaware patient and an
unaware
        clinician combine to affect no change in patient response
info. And
        an owning patient and an owning clinician combine to achieve
the
        maximum increase in patient response info. But intermediate
        engagements are combined differently. Valid alternatives:

            * 'only_participation_counts': if patient is at least
participating, the full extent of increase is realized. Otherwise
nothing. Clinician engagement is ignored.
            * 'patient_only': the progress of patient engagement alone
determines increase, with an unaware patient causing no increase, and
an owning patient causing full increase. Clinician engagement is
ignored.
            * 'heavily_patient': both progress of patient engagement
and progress of clinician engagement are considered, as a weighted
average, heavily weighted toward patients
            * 'slightly_patient': both progress of patient engagement
and progress of clinician engagement are considered, as a weighted
average, slightly weighted toward patient progress
            * 'even_both': both progress of patient engagement and
progress of clinician engagement are considered, and evenly weighted.
            * 'slightly_clinician': both progress of patient
engagement and progress of clinician engagement are considered, as a
```

```
weighted average, slightly weighted toward clinician progress
            * 'heavily_clinician': both progress of patient engagement
and progress of clinician engagement are considered, as a weighted
average, heavily weighted toward clinician
            * 'clinician_only': the progress of clinician engagement
only. Patient engagement is ignored.
            * 'linear_once_participating': the mean progress of
patient engagement and clinician engagement, but with no progress
noted for agents who are only aware

        Default: 'slightly_patient'

    :param patient_response_info_increase_numeric: how does
        patient engagement and clinician engagement combinge to affect
the
        increase in patient response info? This parameter is a
        numeric alternative to **patient_response_info_increase**,
expressing
        the mix of clinician engagement and patient engagement that is
used. A
        value of zero is the same as 'clinician_only'. A value of one
is the
        same as 'patient_only'. A value of 0.5 is the same as
'even_both'. A
        non-None value is used instead of
**patient_response_info_incrase**.
        None indicates to use **patient_reponse_info_increase**.
Default: None
    :type patient_response_info_increase_numeric: float or None

    :param boolean shared_knowledge_affects_patient_response_info_on:
does
        the amount of shared knowledge affect the pace of increase of
        patient response info? For example, shared knowledge may
include
        tools allowing patients to better collect information about
their
        clinicial condition, between clinical encounters.

    :param float patient_response_info_acceleration_from_SK_unit: if
the
        amount of shared knowledge affects the pace of increase of
patient
        response info (i.e.
        **shared_knowledge_affects_patient_response_info_on** is
True), how
        much does a single unit of shared knowledge accelerate the
pace of
        shared patient response info increase? Default: 0.005
```

```
    :param float maximal_patient_response_info_acceleration_from_SK:
if the
        amount of shared knowledge affects the pace of increase of
patient
        response info (i.e.
        **shared_knowledge_affects_patient_response_info_on** is
True),
        and there is a sufficiently large amount of shared knowledge,
what
        is the maximum acceleration of the pace of shared patient
response
        info increase? Default: 0.5

    :param boolean praxis_improves_selection_efficiency_on: does
praxis
        improve selection efficiency, causing a clinician to better
determine
        which treatment package will be most effective for her
patient?
        Default: False
    :param float selection_efficiency_minimum: if praxis does not
improve
        selection efficiency (i.e.
**praxis_improves_selection_efficiency_on**
        is False) or if praxis is 0.0 (i.e. a new patient for a
        traditional clinician in a solo practice), how much selection
        efficiency does the clinician exhibit? Default: 0.0
    :param float selection_efficiency_maximum: if praxis improves
        selection efficiency (i.e.
**praxis_improves_selection_efficiency_on**
        is True), and if praxis for some patient is 1.0 (i.e. the
clinician
        knows as much about that patient's condition and phenotype as
it is
        possible to know), what is the selection efficiency for that
clinician
        working with that patient? Default: 100
    :param selection_efficiency_minmax: specify
**selection_efficiency_minimum**
        and **selection_efficiency_maximum** as a tuple, instead of
individual
        parameeters. Note that if this parameter is non-None,
        **selection_efficiency_minimum** and
**selection_efficiency_maximum**
        are ignored. Default: None
    :type selection_efficiency_minmax: (float, float) or None

    :param boolean previsit_planning_constrains_info_on: does previsit
planning
        constrains the phenotype response info and patient response
```

```
info?
        Default: False
    :param float high_previsit_PRI_constraint: if previsit planning is
high,
        what is the maximum effective patient response info, and the
maximum
        effective phenotype response info? Default: 1.0
    :param float medium_previsit_PRI_constraint: if previsit planning
is medium,
        what is the maximum effective patient response info, and the
maximum
        effective phenotype response info? Default: 0.5
    :param float low_previsit_PRI_constraint: if previsit planning is
low,
        what is the maximum effective patient response info, and the
maximum
        effective phenotype response info? Default: 0.2

    :param boolean praxis_improves_evaluation_accuracy_on: does praxis
improve
        evaluation accuracy? Default: False
    :param float evaluation_accuracy_minimum_praxis: if a clinician
has zero
        (minimal) praxis with a patient, what is their evaluation
accuracy in a
        clinical encounter? Default: 0.10
    :param evaluation_accuracy_maximum_praxis: if a clinician has one
        (maximal) praxis with a patient, what is their evaluation
accuracy in
        a clinical encounter? Alternate parameterization: if the
string value
        "min20" is supplied instead of a float,
        **evaluation_accuracy_maximum_praxis** takes a value of 0.2 of
        **evaluation_accuracy_minimum_praxis**. Default: 0.02
    :type evaluation_accuracy_maximum_praxis: float or str

    :param boolen realtime_usage_enhanced_registry_on: is the enhanced
        registry used in real-time, as a substitute for pre-visit
planning,
        and for patient and clinician activation?
        Note that for real-time usage, not only does this parameter
need to
        be True,
        but the enhanced_registry of the care center must have a value
of
        'contribution and real-time usage'. Default: False
    :param float previsit_obviation_from_ehr_record: if the enhanced
registry
        is used in real-time to substitute for pre-visit planning, how
much
```

```
        of the need for previsit planning is obviated by each record
        in the enhanced registry? Note that the effect tails off at
1.0,
        no matter how many records are in the enhanced registry.
        Default: 0.00005
    :param float activation_obviation_from_ehr_record: if the enhanced
        registry is used in real-time to substitute for patient
activation
        and clinician activation, how much of the need for activation
is
        obviated by each record in the enhanced registry?
        Note that the effect tails off at 1.0,
        no matter how many records are in the enhanced registry.
        Default: 0.00005

    :param float patient_engagement_degree_unaware: if a patient has
an
        activation of unaware, to what extent does he use the learning
        network to increase phenotype response information and
individual
        (patient) response information? Scale: zero to one. Default:
0.0
    :param patient_engagement_degree_aware: if a patient has an
        activation of aware, to what extent does he use the learning
        network to increase phenotype response information and
individual
        (patient) response information? Scale: zero to one.
Alternative
        parameterization: the string "by_participating" will set
        **patient_engagement_degree_aware** to be halfway between
        **patient_engagement_degree_unaware** (usually zero) and
        **patient_engagement_degree_participating**. Default: 0.25
    :type patient_engagement_degree_aware: float or str
    :param float patient_engagement_degree_participating: if a patient
has an
        activation of participating, to what extent does he use the
learning
        network to increase phenotype response information and
individual
        (patient) response information? Scale: zero to one. Default:
0.5
    :param patient_engagement_degree_contributing: if a patient has an
        activation of contributing, to what extent does he use the
learning
        network to increase phenotype response information and
individual
        (patient) response information? Scale: zero to one.
Alternative
        parameterization: the string "by_participating" will set
        **patient_engagement_degree_contributing** to be halfway
```

```
between
        **patient_engagement_degree_participating**  and
        **patient_engagement_degree_owning**. Default: 0.75
    :type patient_engagement_degree_contributing: float or str
    :param float patient_engagement_degree_owning: if a patient has an
        activation of owning, to what extent does he use the learning
        network to increase phenotype response information and
individual
        (patient) response information? Scale: zero to one. Default:
1.0

    :param float clinician_engagement_degree_unaware: if a clinician
has an
        activation of unaware, to what extent does she use the
learning
        network to increase phenotype response information and
individual
        (patient) response information? Scale: zero to one. Default:
0.0
    :param clinician_engagement_degree_aware: if a clinician has an
        activation of aware, to what extent does she use the learning
        network to increase phenotype response information and
individual
        (patient) response information? Scale: zero to one.
Alternative
        parameterization: the string "by_participating" will set
        **clinician_engagement_degree_aware** to be halfway between
        **clinician_engagement_degree_unaware** (usually zero) and
        **clinician_engagement_degree_participating**.Default: 0.25
    :type clinician_engagement_degree_aware: float or str
    :param float clinician_engagement_degree_participating: if a
clinician
        has an
        activation of participating, to what extent does she use the
learning
        network to increase phenotype response information and
individual
        (patient) response information? Scale: zero to one. Default:
0.5
    :param clinician_engagement_degree_contributing: if a clinician
has an
        activation of contributing, to what extent does she use the
learning
        network to increase phenotype response information and
individual
        (patient) response information? Alternative
        parameterization: the string "by_participating" will set
        **clinician_engagement_degree_contributing** to be halfway
between
        **clinician_engagement_degree_participating**  and
```

```
        **clinician_engagement_degree_owning**. Default: 0.75Scale:
zero to one. Default: 0.75
    :type clinician_engagement_degree_contributing: float or str
    :param float clinician_engagement_degree_owning: if a clinician
has an
        activation of owning, to what extent does she use the learning
        network to increase phenotype response information and
individual
        (patient) response information? Scale: zero to one. Default:
1.0

    :param seed: Should the learning model take new draws of all the
random
        variables? In that case, seed should take the value of None.
Or should
        the learning network model take the same draws of all random
        variables as some other learning network? In that case, the
seed
        should take the value of the seed in that other learning
network,
        some integer. Default: None
    """
    def __init__(self, serialize=False, egest=False, **kwargs):
        self._parameters = ModelParameters(**kwargs)
        if serialize:
            self._parameters.serialize_to_file('test')
        if egest:
            self._egest_model_parameters('test', kwargs)
        self._clock = Clock()
        self._trace = Trace(self.clock, self.parameters, self)
        self._schedule = mesa.time.RandomActivation(self)
        self._seed = self._random_seed()
        self._shared_knowledge = SharedKnowledge(self)
        self._enhanced_registry = EnhancedRegistry(self)
        self._phenotypes = self._initial_phenotypes()
        self._treatment_packages = self._initial_treatment_packages()
        self._care_centers = self._initial_care_centers()
        self._clinicians = self._initial_clinicians()
        self._initialize_patients()
        self._trace_current_state()
        super().__init__(self._seed)

    @property
    def patients(self):
        """The patients currently in the cohort in the learning
network.

        Patients changes over the course of a simulation run, as
patients
        are enrolled in the cohort, and as patients leave the cohort.
```

```
        :rtype: list of :class:`Patient`.
        """
        return self._patients

    @property
    def clinicians(self):
        """The clinicians in the learning network.

        :rtype: list of :class:`Clinician`.
        """
        return self._clinicians

    @property
    def care_centers(self):
        """The care_centers in the learning network.

        :rtype: list of :class:`CareCenter`.
        """
        return self._care_centers

    @property
    def treatment_packages(self):
        """The alternative treatment packages used to treat patients.

        :rtype: list of :class:`TreatmentPackage`."""
        return self._treatment_packages

    @property
    def phenotypes(self):
        """The phenotypes. Each patient exhibits one phenotype.

        :rtype: list of :class:`Phenotype`."""
        return self._phenotypes

    @property
    def shared_knowledge(self):
        """The shared knowledge, available to all patients and
clinicians.

        :rtype: SharedKnowledge
        """
        return self._shared_knowledge

    @property
    def commons(self):
        """Same as shared knowledge"""
        return self._shared_knowledge

    @property
```

```
    def parameters(self):
        """The values of all the static model parameters.

        The model is initialized with a large number of static
parameters.
        These parameter values are managed in an instance of
        :class:`ModelParameters`.

        :rtype: ModelParameters
        """
        return self._parameters

    def parameter_values(self):
        """Return all parameter values as a dict."""
        return self.parameters.as_dict()

    @property
    def clock(self):
        """The current week of the simulation.

        :rtype: Clock
        """
        return self._clock

    @property
    def trace(self):
        """The complete history of the simulation run.

        The complete history of the simulation run, including
everything
        that changed and the week it changed.

        :rtype: Trace
        """
        return self._trace

    @property
    def enhanced_registry(self):
        return self._enhanced_registry

    def _total_planned_clinicians(self):
        """How many clinicians in total, when everyone is added?"""
        return sum(self.planned_clinicians())

    def planned_clinicians(self):
        """How many clinicians are planned for each care center?"""
        return (cc['clinician_count'] for cc in
self.parameters.care_centers)

    # There are three different counts of patients:
```

```
    # * planned_patients() --- how many patients are planned in the
    #     cohort, from the initial specification. A list of counts by
care
    #     center.
    # * patients_left_to_add --- how many patients of the initial
cohort are left
    #     to add, when patients_gradual_on is true. A simple int
    # * patient_counts() --- how many patients are in each care center
now,
    #     including both the initial cohort and those added to the
cohort later,
    #     if cohort_entrance_on is true. Returns a list of counts by
care center

    def _total_planned_patients(self):
        """How many patients in total, when everyone is added?"""
        return sum(self.planned_patients())

    def planned_patients(self):
        """How many patients are planned for each care center?"""
        return (cc['patient_count'] for cc in
self.parameters.care_centers)

    def patient_counts(self):
        """Return list of current counts of patients in care
centers."""
        return [cc.patient_count() for cc in self.care_centers]

    def _random_seed(self):
        """Set random seed, perhaps per a parameter."""
        if self.parameters.seed:      # pragma: no cover
            seed = self.parameters.seed
        else:
            # using a different random number generater to generate
seed
            seed = random.randint(0, 999999)
        np.random.seed(seed)
        self.reset_randomizer(seed) # Mesa has its own RNG
        # print('Seed: {}'.format(seed))
        return seed

    def _initial_phenotypes(self):
        """Return the initial list of phenotypes."""
        phenotype_count = self.parameters.phenotype_count
        try:
            assert phenotype_count >= 1
            return [Phenotype(i) for i in range(phenotype_count)]
        except:
            raise LearningNetworkModelException(
                '{} phenotypes not allowed'.format(phenotype_count))
```

```
    def _initial_treatment_packages(self):
        """Return initial list of treatment packages."""
        return [
            TreatmentPackage(
                i, self.phenotypes,

self.parameters.estimated_treatment_package_effect_mean,
                self.parameters.estimated_treatment_package_effect_sd)
            for i in range(self.parameters.treatment_package_count)]

    def _initial_care_centers(self):
        """Create the care centers"""
        assign_spec = self.parameters.clinician_assignment
        ccs = self.parameters.care_centers
        def _create_care_center(cc_spec, i):
            pcount = cc_spec['patient_count']
            ccount = cc_spec['clinician_count']
            kwargs = {
                k:v for k,v in cc_spec.items()
                if k == "previsit_planning" or k ==
"enhanced_registry"
            }
            return CareCenter(pcount, ccount, assign_spec, i+1,
**kwargs)
        return [
            _create_care_center(cc_spec, i)
            for i, cc_spec in enumerate(self.parameters.care_centers)
        ]

    def _initial_clinicians(self):
        """Create and the initial list of clinicians."""
        params = self.parameters
        self._clinician_factory = ClinicianFactory(
            self, 0, params.clinician_states
        )
        designator = CareCenterDesignator(
            self.care_centers, agent_type="clinician"
        )
        new_clinicians = [
            self._clinician_factory.create(designator.next())
            for count in self.planned_clinicians() for _ in
range(count)
        ]
        self._add_clinician_influence_edges(new_clinicians)
        for c in new_clinicians:
            self._schedule.add(c)
        return new_clinicians

    def _add_clinician_influence_edges(self, new_clinicians):
```

```
        """Add edges between clinicians who influence each other."""
        probs = self._clinician_infl_probs()
        for c1, c2 in itertools.combinations(new_clinicians, 2):
            if c1.care_center == c2.care_center:
                p = probs["within_CC"]
            else:
                p = probs["cross_CC"]
            self._maybe_add_influence_edge(c1, c2, p)

    def _clinician_infl_probs(self):
        """Calc probabilities (within, across) of edge between two
clinicians"""
        return edge_probs(
            list(self.planned_clinicians()),
            self.parameters._clinician_network_edges,
            self.parameters._clinician_influence_across_prop
        )

    def _patient_infl_probs(self):
        """Calc probabilities (within, across) of edge between two
patients"""
        return edge_probs(
            list(self.planned_patients()),
            self.parameters._patient_network_edges,
            self.parameters._patient_influence_across_prop
        )

    def _patient_infl_probs_later(self, counts_prior,
counts_posterior):
        """Calc probs of edge between 2 patients after intiial
cohort"""
        return edge_probs_later(
            counts_prior,
            counts_posterior,
            self.parameters._patient_network_edges,
            self.parameters._patient_influence_across_prop
        )

    def _initialize_patients(self):
        """Initialize patients."""
        self._patients = []
        params = self.parameters
        self._patient_factory = PatientFactory(
            self,
            self._total_planned_clinicians(),
            params.initial_outcome_distr,
            params.patient_states
        )
        self._patient_designator =
CareCenterDesignator(self.care_centers)
```

```
        self._setup_influence_probs()
        if params.patients_gradual_on:
            self._setup_gradual_patients()
            self._add_initial_patients_gradually()
        else:
            self._add_patients('initial',
self._total_planned_patients())

    def _setup_influence_probs(self):
        """Set up the influence probabilities for the initial
cohort"""
        self._initial_cohort_influence_probs =
self._patient_infl_probs()

    def _setup_gradual_patients(self):
        """Set up attributes to use for adding patients gradually"""
        self._patients_left_to_add = self._total_planned_patients()

    def _trace_current_state(self):
        """Trace the current state of the model."""
        self.trace.trace_patients(self.patients)
        self.trace.trace_clinicians(self.clinicians)
        self.trace.trace_shared_knowledge(self.shared_knowledge.level)

    def step(self, weeks=1):
        """Simulate several weeks.

        Run the simulation for a number of weeks. After running, you
        can examine model.trace to see what happened. And then you can
        simulate some more weeks.

        :param int weeks: number of weeks to simulate. Default: 1
        """
        for i in range(weeks):
            self._step_one()

    def _step_one(self):
        """Advance by one week."""
        self.clock.advance()
        self.shared_knowledge.step()
        self._schedule.step()
        self.enhanced_registry.step()
        self._maybe_add_patients()
        self._trace_current_state()

    def _maybe_add_patients(self):
        """Maybe add one or more patients this week."""
        params = self.parameters
        if params.patients_gradual_on and self._patients_left_to_add >
0:
```

```
            self._add_initial_patients_gradually()
        if params.cohort_entrance_on:
            self._maybe_add_subsequent_patients()
        if params.replace_departing_patients_on:
            self._maybe_replace_departures()

    def _add_initial_patients_gradually(self):
        """Add some more initial patients, gradually. After"""
        qty = min(
            self._patients_left_to_add,
            math.ceil(
                self._total_planned_patients() /
                self.parameters.patients_gradual_duration
            )
        )
        self._add_patients('initial', qty)
        self._patients_left_to_add -= qty

    def _maybe_add_subsequent_patients(self):
        """Maybe add some patients, in addition to those from initial
cohort."""
        expected_qty = (
            self.parameters._cohort_entrance_per_month /
_weeks_per_month()
        )
        qty_added = np.random.poisson(expected_qty)
        if qty_added > 0:
            self._add_patients('added later', qty_added)

    def _maybe_replace_departures(self):
        """Maybe replace some of the departed patients, in the same
CCs"""
        for cc in self.care_centers:
            cc.maybe_replace_departures(self)

    def _add_patients(self, reason, n):
        """Add n patients."""
        for i in range(n):
            self._add_patient(reason)

    def _add_patient(self, reason, care_center=None):
        """Add a patient to the cohort."""
        pd = self._patient_designator
        if care_center is None:
            care_center = pd.next(reason=reason)
        counts_before = self.patient_counts()
        new_patient = self._patient_factory.create(care_center)
        if reason == "replace":
            # print('Adding {} to {} with condition {}'.format(
            #     new_patient, care_center, new_patient.outcome
```

```
            #     )
            # )
            pass
        counts_after = self.patient_counts()
        existing_patients = self._patients.copy() #ugh: O(n^2) for
adding many
        self._patients.append(new_patient)
        self._schedule.add(new_patient)
        if reason == "initial":
            probs = self._initial_cohort_influence_probs
        else:
            probs = self._patient_infl_probs_later(counts_before,
counts_after)
        self._add_patient_edges(new_patient, existing_patients, probs)
        self.trace.trace_cohort_entrance(
            new_patient, reason, new_patient.outcome
        )

    def _add_patient_edges(self, new_patient, existing_patients,
probs):
        """Add influence edges between this patient and existing
ones."""
        for xp in existing_patients:
            if new_patient.care_center == xp.care_center:
                prob = probs["within_CC"]
            else:
                prob = probs["cross_CC"]
            self._maybe_add_influence_edge(new_patient, xp, prob)

    def _maybe_add_influence_edge(self, a1, a2, prob):
        """Maybe add an influence edge between these two agents."""
        if np.random.random() < prob:
            a1.add_influence_edge(a2)
            a2.add_influence_edge(a1)
            self.trace.trace_influence_edge(a1, a2)

    def remove_patient_from_cohort(self, patient):
        """Remove the patient from the cohort.

        A patient is exiting the cohort, perhaps because he
        moved away, perhaps because he aged out. Remove him from the
        simulation.

        :param Patient patient: the patient who is exiting
        """
        patient.care_center.remove_patient(patient)
        self._schedule.remove(patient)
        self.patients.remove(patient)

    def results_for_display(self):
```

```
        """The trace of the simulation, in a display format.

        Used by user interfaces to retrieve the results of the
simulation
        for display. Note that these results are a small subset of
what is
        available in the full trace, suitable for storage as hidden
HTML
        fields.

        :returns: simulation run results, suitable for display
        :rtype: dict with the keys 'patients', 'patient_aggregates',
            'patient_state_count', 'clinician_state_count',
'shared_knowledge',
            and 'seed'
        """
        tr = self.trace
        return {
            'patients': tr.patient_display_results(),
            'patient_aggregates':
tr.patient_aggregate_display_results(),
            'patient_state_count':
tr.count_patients_in_states_over_time(),
            'clinician_state_count':
tr.count_clinicians_in_states_over_time(),
            'shared_knowledge': tr.shared_knowledge_display_results(),
            'seed': self._seed,
        }

    def results_for_download(self):
        """The trace of the simulation, in a format ready for
download.

        Used by user interfaces to retrieve all results of the
simulation,
        for CSV download. This is everything: the entire trace of the
        simulation. The strings are huge.

        :returns: simulation run results, suitable for downloads
        :rtype: dict with the keys 'patients', 'clinicians',
'encounters',
            'transitions', 'treatment_packages', 'relapses',
'shared_knowledge',
            'shared_knowledge_changes', 'cohort_changes', and
'influence_edges'.
            The dict values are string encodings of CSV files.
        """
        tr = self.trace
        def _create_csv(method):
            """Return a properly-coded CSV."""
```

```
            return getattr(tr, method)().to_csv(encoding='utf-8',
index=False)

        return {k:_create_csv(k) for k in [
            'patients', 'clinicians', 'encounters', 'transitions',
            'treatment_packages', 'relapses', 'shared_knowledge',
            'shared_knowledge_changes', 'cohort_changes',
'influence_edges']}

    def _egest_model_parameters(self, path_prefix, params):
        """Write a file of all model paramters, for debugging
purposes"""
        def _egest_parameter(param, value, f):
            if isinstance(value, str):
                print(f'{param}="{value}",', file=f)
            elif isinstance(
                value, (TransitionDeterminer, ContributionDeterminer)
            ):
                pass
            else:
                print(f'{param}={value},', file=f)

        with open(f'{path_prefix}_raw_model_parameters.txt', 'w') as
f:
            for param, value in params.items():
                _egest_parameter(param, value, f)


#
# Managing the parameters of a learning network model
#

# bogus first line of docstring to suppress autodoc superlong args
class ModelParameters:
    """__init__(**kwargs)

    Parameters for a model run.

    Some of these parameters are supplied
to :class:`LearningNetworkModel`,
    others are derived from supplied parameters. All parameters
managed by
    this class are static: they do not change over the course of a
model run.

    Many other classes retain references to an instance of this class,
if
    they need to reference model parameter settings.

    This class is initialized with exactly the same parameters as
```

```
    :class:`LearningNetworkModel`, because this class just serves to 
manage
    the parameters of the model.
    """
    parameters = [
        'care_centers', 'previsit_planning', 'enhanced_registry',
        'patients_gradual_on', 'patients_gradual_duration',
        'cohort_entrance_on', 'cohort_entrance_per_month', 
'cohort_exit_on',
        'age_out_annual_proportion', 'move_away_annual_proportion',
        'get_better_annual_proportion', 'get_better_start_outcome',
        'get_worse_annual_proportion', 'get_worse_end_outcome',
        'replace_departing_patients_on',
        'phenotype_count', 'initial_outcome_distr', 'random_walk_on',
        'random_walk_mean', 'random_walk_sd', 'relapse_on',
        'relapse_period', 'relapse_amount', 'treatment_package_on',
        'treatment_package_count', 'treatment_package_effect_mean',
        'treatment_package_effect_sd',
        'best_treatment_package_effect',
        'worst_treatment_package_effect',
        'clinician_assignment', 'encounter_period', 'patient_states',
        'clinician_states', 'encounter_aware_on',
        'encounter_aware_likelihood', 'encounter_activate_on',
        'patient_activate_determiner', 'encounter_dispirit_on',
        'patient_dispirit_determiner', 'patient_influence_on',
        'patient_network_edges', 'patient_influence_across_prop',
        'patient_influence_become_aware_probability',
        'patient_influence_activation_probability',
        'clinician_influence_on', 'clinician_network_edges',
        'clinician_influence_across_prop',
        'clinician_influence_become_aware_probability',
        'clinician_influence_activation_probability',
        'clinician_dispirit_on', 'clinician_dispirit_probability',
        'shared_knowledge_initial', 'shared_knowledge_decays_on',
        'shared_knowledge_half_life',
        'patient_contributes_shared_knowledge_on',
        'patient_shared_knowledge_contrib_determiner',
        'clinician_contributes_shared_knowledge_on',
        'clinician_shared_knowledge_contrib_determiner',
        'enhanced_registry_initial',
        'enhanced_registry_affects_commons_on',
        'enhanced_registry_analysis_period',
        'enhanced_registry_records_per_commons_item',
        'phenotype_response_info_affects_praxis_on',
        'potential_phenotype_response_info_from_SK_unit',
        'phenotype_realization',
        'phenotype_realization_numeric',
        'patient_response_info_affects_praxis_on',
        'patient_response_info_initial', 
'patient_response_info_half_life',
```

```
        'patient_response_info_increase',
        'patient_response_info_increase_numeric',
        'shared_knowledge_affects_patient_response_info_on',
        'patient_response_info_acceleration_from_SK_unit',
        'maximal_patient_response_info_acceleration_from_SK',
        'praxis_improves_selection_efficiency_on',
        'selection_efficiency_minimum',
        'selection_efficiency_maximum',
        'previsit_planning_constrains_info_on',
        'previsit_PRI_constraints',
        'praxis_improves_evaluation_accuracy_on',
        'evaluation_accuracy_minimum_praxis',
        'evaluation_accuracy_maximum_praxis',
        'realtime_usage_enhanced_registry_on',
        'previsit_obviation_from_ehr_record',
        'activation_obviation_from_ehr_record',
        'patient_engagement_degree',
        'clinician_engagement_degree',
        'seed'
    ]
    def __init__(
            self, care_centers=None,
            previsit_planning='low', enhanced_registry='no
contribution',
            patients_gradual_on=False, patients_gradual_duration=13,
            cohort_entrance_on=False, cohort_entrance_per_month=10,
            cohort_exit_on=False, age_out_annual_proportion=0.1,
            move_away_annual_proportion=0.05,
            get_better_annual_proportion=0.5,
get_better_start_outcome=0.9,
            get_worse_annual_proportion=0.8,
get_worse_end_outcome=0.1,
            replace_departing_patients_on=False,

            phenotype_count=4, initial_outcome_distr='uniform_0_1',

            random_walk_on=False, random_walk_mean=0.0,
random_walk_sd=0.01,

            relapse_on=False, relapse_period=52, relapse_amount=0.5,

            treatment_package_on=False,
            treatment_package_count=8,
            treatment_package_effect_mean=0.0,
            treatment_package_effect_sd=0.02,
            best_treatment_package_effect=None,
            worst_treatment_package_effect=None,

            clinician_assignment='random',
            encounter_period=None,
```

```
            patient_states='all_unaware',
clinician_states='all_aware',
            encounter_aware_on=False, encounter_aware_likelihood=0.5,

            encounter_activate_on=False,

patient_activate_determiner=TransitionDeterminer.default_activate(),
            encounter_dispirit_on=False,

patient_dispirit_determiner=TransitionDeterminer.default_dispirit(),

            patient_influence_on=False, patient_network_edges=0,
            patient_influence_across_prop=0.0,
            patient_influence_become_aware_probability=0.1,
            patient_influence_activation_probability=0.1,

            clinician_influence_on=False, clinician_network_edges=0,
            clinician_influence_across_prop=0.0,
            clinician_influence_become_aware_probability=0.8,
            clinician_influence_activation_probability=0.5,

            clinician_dispirit_on=False,
clinician_dispirit_probability=0.1,

            shared_knowledge_initial=0.0,
            shared_knowledge_decays_on=False,
            shared_knowledge_half_life=153,

            patient_contributes_shared_knowledge_on=False,
            patient_shared_knowledge_contrib_determiner=(
                ContributionDeterminer.default_for_patients()
            ),

            clinician_contributes_shared_knowledge_on=False,
            clinician_shared_knowledge_contrib_determiner=(
                ContributionDeterminer.default_for_clinicians()
            ),

            enhanced_registry_initial=0,
            enhanced_registry_initial_per_patient=None,
            enhanced_registry_affects_commons_on=False,
            enhanced_registry_analysis_period=13,
            enhanced_registry_records_per_commons_item=1000,

            phenotype_response_info_affects_praxis_on=False,
            potential_phenotype_response_info_from_SK_unit=0.001,
            phenotype_realization='linear_once_participating',
            phenotype_realization_numeric=None,
```

```
            patient_response_info_affects_praxis_on=False,
            patient_response_info_initial=0,
            patient_response_info_half_life=20,
            patient_response_info_increase='slightly_patient',
            patient_response_info_increase_numeric=None,

            shared_knowledge_affects_patient_response_info_on=False,
            patient_response_info_acceleration_from_SK_unit=0.005,
            maximal_patient_response_info_acceleration_from_SK=0.5,

            praxis_improves_selection_efficiency_on=False,
            selection_efficiency_minimum=0,
            selection_efficiency_maximum=100,
            selection_efficiency_minmax=None,

            previsit_planning_constrains_info_on = False,
            high_previsit_PRI_constraint=1.0,
            medium_previsit_PRI_constraint=0.5,
            low_previsit_PRI_constraint=0.2,

            praxis_improves_evaluation_accuracy_on=False,
            evaluation_accuracy_minimum_praxis=0.10,
            evaluation_accuracy_maximum_praxis=0.02,

            realtime_usage_enhanced_registry_on=False,
            previsit_obviation_from_ehr_record=0.00005,
            activation_obviation_from_ehr_record=0.00005,

            patient_engagement_degree_unaware=0.0,
            patient_engagement_degree_aware=0.25,
            patient_engagement_degree_participating=0.5,
            patient_engagement_degree_contributing=0.75,
            patient_engagement_degree_owning=1.0,
clinician_engagement_degree_unaware=0.0,
            clinician_engagement_degree_aware=0.25,
            clinician_engagement_degree_participating=0.5,
            clinician_engagement_degree_contributing=0.75,
            clinician_engagement_degree_owning=1.0,

            seed=None
        ):

        self._set_care_centers(
            care_centers, previsit_planning, enhanced_registry
        )

        self._patients_gradual_on = patients_gradual_on
        self._patients_gradual_duration = patients_gradual_duration

        self._cohort_entrance_on = cohort_entrance_on
```

```
        self._cohort_entrance_per_month = cohort_entrance_per_month

        self._cohort_exit_on = cohort_exit_on
        self._age_out_annual_proportion = age_out_annual_proportion
        self._move_away_annual_proportion =
move_away_annual_proportion
        self._get_better_annual_proportion =
get_better_annual_proportion
        self._get_better_start_outcome = get_better_start_outcome
        self._get_worse_annual_proportion =
get_worse_annual_proportion
        self._get_worse_end_outcome = get_worse_end_outcome
        self._replace_departing_patients_on =
replace_departing_patients_on

        self._phenotype_count = phenotype_count
        self._initial_outcome_distr = initial_outcome_distr

        self._random_walk_on = random_walk_on
        self._random_walk_mean = random_walk_mean
        self._random_walk_sd = random_walk_sd

        self._relapse_on = relapse_on
        self._relapse_period = relapse_period
        self._relapse_amount = relapse_amount

        self._treatment_package_on = treatment_package_on
        self._treatment_package_count = treatment_package_count
        self._treatment_package_effect_mean =
treatment_package_effect_mean
        self._treatment_package_effect_sd =
treatment_package_effect_sd
        self._best_treatment_package_effect =
best_treatment_package_effect
        self._worst_treatment_package_effect =
worst_treatment_package_effect
        self._maybe_estimate_treatment_package_effects()

        self._clinician_assignment=clinician_assignment
        self._encounter_period = encounter_period

        self._patient_states = patient_states
        self._clinician_states = clinician_states
        self._encounter_aware_on = encounter_aware_on
        self._encounter_aware_likelihood = encounter_aware_likelihood

        self._encounter_activate_on = encounter_activate_on
        self._patient_activate_determiner = (

self._patient_activate_parameterization(patient_activate_determiner)
```

```
        )
        self._encounter_dispirit_on = encounter_dispirit_on
        self._patient_dispirit_determiner = (

self._patient_dispirit_parameterization(patient_dispirit_determiner)
        )

        self._patient_influence_on=patient_influence_on
        self._patient_network_edges=patient_network_edges

self._patient_influence_across_prop=patient_influence_across_prop
        self._patient_influence_become_aware_probability=(
            patient_influence_become_aware_probability)
        self._patient_influence_activation_probability=(
            patient_influence_activation_probability)

        self._clinician_influence_on = clinician_influence_on
        self._clinician_network_edges = clinician_network_edges
        self._clinician_influence_across_prop =
clinician_influence_across_prop
        self._clinician_influence_become_aware_probability = (
            clinician_influence_become_aware_probability
        )
        self._clinician_influence_activation_probability = (
            clinician_influence_activation_probability
        )
        self._clinician_dispirit_on = clinician_dispirit_on
        self._clinician_dispirit_probability =
clinician_dispirit_probability

        self._shared_knowledge_initial=shared_knowledge_initial
        self._shared_knowledge_decays_on=shared_knowledge_decays_on
        self._shared_knowledge_half_life=shared_knowledge_half_life

        self._patient_contributes_shared_knowledge_on = (
            patient_contributes_shared_knowledge_on)
        self._patient_shared_knowledge_contrib_determiner = (

self._commons_contrib(patient_shared_knowledge_contrib_determiner)
        )

        self._clinician_contributes_shared_knowledge_on = (
            clinician_contributes_shared_knowledge_on)
        self._clinician_shared_knowledge_contrib_determiner = (

self._commons_contrib(clinician_shared_knowledge_contrib_determiner)
        )

        self._enhanced_registry_initial =
self._calc_enhanced_registry_initial(
```

```
            enhanced_registry_initial,
enhanced_registry_initial_per_patient
        )

        self._enhanced_registry_affects_commons_on = (
            enhanced_registry_affects_commons_on
        )
        self._enhanced_registry_analysis_period = (
            enhanced_registry_analysis_period
        )
        self._enhanced_registry_records_per_commons_item = (
            enhanced_registry_records_per_commons_item
        )

        self._phenotype_response_info_affects_praxis_on = (
            phenotype_response_info_affects_praxis_on)
        self._potential_phenotype_response_info_from_SK_unit=(
            potential_phenotype_response_info_from_SK_unit)
        self._phenotype_realization=phenotype_realization

self._phenotype_realization_numeric=phenotype_realization_numeric

        self._patient_response_info_affects_praxis_on = (
            patient_response_info_affects_praxis_on)
        self._patient_response_info_initial =
patient_response_info_initial
        self._patient_response_info_half_life =
patient_response_info_half_life
        self._patient_response_info_increase =
patient_response_info_increase
        self._patient_response_info_increase_numeric = \
            patient_response_info_increase_numeric

        self._shared_knowledge_affects_patient_response_info_on = (
            shared_knowledge_affects_patient_response_info_on)
        self._patient_response_info_acceleration_from_SK_unit= (
            patient_response_info_acceleration_from_SK_unit)
        self._maximal_patient_response_info_acceleration_from_SK=(
            maximal_patient_response_info_acceleration_from_SK)

        self._praxis_improves_selection_efficiency_on = \
            praxis_improves_selection_efficiency_on
        if selection_efficiency_minmax is None:
            self._selection_efficiency_minimum =
selection_efficiency_minimum
            self._selection_efficiency_maximum =
selection_efficiency_maximum
        else:
            self._selection_efficiency_minimum =
selection_efficiency_minmax[0]
```

```
            self._selection_efficiency_maximum =
selection_efficiency_minmax[1]

        self._previsit_planning_constrains_info_on = \
            previsit_planning_constrains_info_on
        self._previsit_PRI_constraints = {
            'high': high_previsit_PRI_constraint,
            'medium': medium_previsit_PRI_constraint,
            'low':  low_previsit_PRI_constraint
        }

        self._praxis_improves_evaluation_accuracy_on = \
            praxis_improves_evaluation_accuracy_on
        self._evaluation_accuracy_minimum_praxis = \
            evaluation_accuracy_minimum_praxis
        if evaluation_accuracy_maximum_praxis == "min20":
            self._evaluation_accuracy_maximum_praxis = (
                evaluation_accuracy_minimum_praxis * 0.2
            )
        else:
            self._evaluation_accuracy_maximum_praxis = (
                evaluation_accuracy_maximum_praxis
            )

        self._realtime_usage_enhanced_registry_on = \
            realtime_usage_enhanced_registry_on
        self._previsit_obviation_from_ehr_record = \
            previsit_obviation_from_ehr_record
        self._activation_obviation_from_ehr_record = \
            activation_obviation_from_ehr_record

        if patient_engagement_degree_aware == "by_participating":
            patient_engagement_degree_aware = (
                (
                    patient_engagement_degree_unaware +
                    patient_engagement_degree_participating
                ) / 2
            )
        if patient_engagement_degree_contributing ==
"by_participating":
            patient_engagement_degree_contributing = (
                (
                    patient_engagement_degree_participating +
                    patient_engagement_degree_owning
                ) / 2
            )
        self._patient_engagement_degree = {
            'unaware': patient_engagement_degree_unaware,
            'aware': patient_engagement_degree_aware,
            'participating': patient_engagement_degree_participating,
```

```
            'contributing': patient_engagement_degree_contributing,
            'owning': patient_engagement_degree_owning,
        }

        if clinician_engagement_degree_aware == "by_participating":
            clinician_engagement_degree_aware = (
                (
                    clinician_engagement_degree_unaware +
                    clinician_engagement_degree_participating
                ) / 2
            )
        if clinician_engagement_degree_contributing ==
"by_participating":
            clinician_engagement_degree_contributing = (
                (
                    clinician_engagement_degree_participating +
                    clinician_engagement_degree_owning
                ) / 2
            )
        self._clinician_engagement_degree = {
            'unaware': clinician_engagement_degree_unaware,
            'aware': clinician_engagement_degree_aware,
            'participating':
clinician_engagement_degree_participating,
            'contributing': clinician_engagement_degree_contributing,
            'owning': clinician_engagement_degree_owning,
        }

        self._seed = seed

    def _set_care_centers(
        self, care_centers, previsit_planning, enhanced_registry
    ):
        """Set value for care centers, based on what was passed."""
        if isinstance(care_centers, float):
            care_centers = round(care_centers)
        if isinstance(care_centers, int):
            care_centers = [
                {
                    "patient_count": 200,
                    "clinician_count": 10,
                    "previsit_planning": previsit_planning,
                    "enhanced_registry": enhanced_registry,
                }
                for i in range(care_centers)
            ]
        if care_centers:
            self._validate_care_centers(care_centers)
            self._care_centers = care_centers
        else:
```

```
            self._care_centers = [{'patient_count': 200,
'clinician_count': 10}]
        self._previsit_planning = previsit_planning
        self._enhanced_registry = enhanced_registry

    def _validate_care_centers(self, care_centers):
        """Raise error if invalid specification of care centers"""
        def check_arguments(
            previsit_planning="low",
            enhanced_registry="no contribution",
            *,
            patient_count=None,
            clinician_count=None
        ):
            assert isinstance(patient_count, int), (
                f"Care center patient_count {patient_count} is not an
int"
            )
            assert isinstance(clinician_count, int), (
                f"Care center clinician_count {clinician_count} is not
an int"
            )
            assert previsit_planning in ['high', 'medium', 'low'], (
                    f"Care center previsit_planning
{previsit_planning} is "
                    "not 'high', 'medium', or 'low'"
                )
            valid_contrs = [
                'no contribution',
                'contribution',
                'contribution and real-time usage'
            ]
            assert enhanced_registry in valid_contrs, (
                "Care center enhanced_registry must be one of:
{}".format(
                    ", ".join(valid_contrs)
                )
            )

        for care_center in care_centers:
            check_arguments(**care_center)

    def _maybe_estimate_treatment_package_effects(self):
        """Estimate treatment package effects if best and worst
provided"""
        best = self._best_treatment_package_effect
        worst = self._worst_treatment_package_effect
        if best is not None and worst is not None:
            pkg_count = self.treatment_package_count
            mean, sd = self._estimate_treatment_package_effects(
```

```
                best, worst, pkg_count
            )
        else:
            mean = self._treatment_package_effect_mean
            sd = self._treatment_package_effect_sd

        self._estimated_treatment_package_effect_mean = mean
        self._estimated_treatment_package_effect_sd = sd

    def _commons_contrib(self, param_value):
        """Determine commons contribution determiner"""
        if isinstance(param_value, (int, float)):
            return
ContributionDeterminer.specified_numerically(param_value)
        elif isinstance(param_value, collections.abc.Mapping):
            return ContributionDeterminer(param_value)
        else:
            return param_value

    def _patient_activate_parameterization(self, param_value):
        """Patient activate determiner, speccified in one of 3 ways"""
        if isinstance(param_value, (int, float)):
            return
TransitionDeterminer.activate_specified_numerically(
                param_value
            )
        elif isinstance(param_value, collections.abc.Mapping):
            return TransitionDeterminer(dict=param_value)
        else:
            return param_value

    def _patient_dispirit_parameterization(self, param_value):
        """Patient dispirit determiner, specified in one of 3 ways"""
        if isinstance(param_value, (int, float)):
            return
TransitionDeterminer.dispirit_specified_numerically(
                param_value
            )
        elif isinstance(param_value, collections.abc.Mapping):
            return TransitionDeterminer(dict=param_value)
        else:
            return param_value

    def _calc_enhanced_registry_initial(self, initial,
initial_per_patient):
        """Determine initial quantity in enhanced registry"""
        if initial_per_patient is not None:
            return initial_per_patient * self._count_patients()
        else:
            return initial
```

```
    def _count_patients(self):
        """How many patients will there be?"""
        return sum(cc["patient_count"] for cc in self.care_centers)

    @staticmethod
    def _estimate_treatment_package_effects(best, worst, pkg_count):
        """Estimate mean and SD of treatment package effects"""
        # set numpy seed just for this estimation
        # it is set for real in model initialization
        np.random.seed(19950526)
        random_effects = np.random.normal(size=(pkg_count, 1000))
        mins = np.amin(random_effects, axis=0)
        maxs = np.amax(random_effects, axis=0)
        mean_range = np.mean(maxs - mins)
        estd_mean = (best + worst) / 2
        estd_sd = (best - worst) / mean_range
        return estd_mean, estd_sd

    @property
    def care_centers(self):
        """Specification of care centers.

        :rtype: list of dict
        """
        return self._care_centers
    @property
    def patients_gradual_on(self):
        """Are patients in the initial cohort added gradually?

        gradually = True; all at once = False

        :rtype: boolean
        """
        return self._patients_gradual_on
    @property
    def patients_gradual_duration(self):
        """Over how many weeks are all the initial patients added?

        :rtype: int
        """
        return self._patients_gradual_duration

    @property
    def cohort_entrance_on(self):
        """Do patients enter the cohort later, e.g. because they move
there?

        :rtype: boolean
        """
```

```
        return self._cohort_entrance_on
    @property
    def cohort_entrance_per_month(self):
        """How many patients are added every month, on average?

        :rtype: float
        """
        return self._cohort_entrance_per_month

    @property
    def cohort_exit_on(self):
        """Do patients leave the cohort, e.g. becuase they move away?

        :rtype: boolean
        """
        return self._cohort_exit_on
    @property
    def get_better_annual_proportion(self):
        """What proportion of good outcome patients leave the cohort
each year?

        :rtype: float
        """
        return self._get_better_annual_proportion
    @property
    def get_better_start_outcome(self):
        """What outcome is good enough to cause some patients to exit
cohort?

        :rtype: float
        """
        return self._get_better_start_outcome
    @property
    def get_worse_annual_proportion(self):
        """What proportion of poor outcome patients leave the cohort
each year?

        :rtype: float
        """
        return self._get_worse_annual_proportion
    @property
    def get_worse_end_outcome(self):
        """What outcome is poor enough to cause some patients to exit
cohort?

        :rtype: float
        """
        return self._get_worse_end_outcome
    @property
    def replace_departing_patients_on(self):
```

```
        return self._replace_departing_patients_on

    @property
    def phenotype_count(self):
        """How many distinct patient phenotypes?

        :rtype: int
        """
        return self._phenotype_count
    @property
    def initial_outcome_distr(self):
        """Specification of initial outcome probability distribution.

        Potential values:

            * 'uniform_0_1': uniform distribution between 0.0 and 1.0
            * 'uniform_08_1': uniform distribution between 0.8 and 1.0
            * 'uniform_0_03': uniform distribution between 0.0 and 0.3
            * 'beta_2_5': a beta distribution with alpha=2 and beta=5.
That distribution has a mode of 0.2 and a median of roughly 0.26
            * 'beta_3_4': a beta distribution with alpha=3 and beta=4
            * 'beta_3-5_3-5': a beta distribution with alpha and beta
both 3.5
            * 'beta_4_3': a beta distribution with alpha=4 and beta=3
            * 'beta_5_2': a beta distribution with alpha-5 and beta=2
            * a list of floats (e.g. [0.0, 0.4, 0.6]), indicating that
outcomes should cycle through the list, one patient at a time.

        :rtype: str
        """
        return self._initial_outcome_distr

    @property
    def random_walk_on(self):
        """Patient outcome varies from week to week?

        :rtype: boolean
        """
        return self._random_walk_on
    @property
    def random_walk_mean(self):
        """Mean of weekly random outcome variance.

        :rtype: float
        """
        return self._random_walk_mean
    @property
    def random_walk_sd(self):
        """Standard deviation of weekly random outcome variance.
```

```
        :rtype: float
        """
        return self._random_walk_sd

    @property
    def relapse_on(self):
        """Patients sometimes relapse?

        :rtype: boolean
        """
        return self._relapse_on
    @property
    def relapse_probability(self):
        """Weekly probability of relapse.

        :rtype: float
        """
        return 1.0 / self._relapse_period
    @property
    def relapse_amount(self):
        """Relapse amount, when it occurs.

        :rtype: float
        """
        return self._relapse_amount

    @property
    def treatment_package_on(self):
        """Treatment package affects patient outcomes?

        :rtype: boolean
        """
        return self._treatment_package_on
    @property
    def treatment_package_count(self):
        """How many treatment package alternatives exist?

        :rtype: int
        """
        return self._treatment_package_count
    @property
    def estimated_treatment_package_effect_mean(self):
        """Mean effectiveness of treatment package, across phenotypes.

        :rtype: float
        """
        return self._estimated_treatment_package_effect_mean
    @property
    def estimated_treatment_package_effect_sd(self):
        """Effectiveness standard deviation of treatment package,
```

```
across phenos

        :rtype: float
        """
        return self._estimated_treatment_package_effect_sd

    @property
    def clinician_assignment(self):
        """How are clinicians assigned to patients?

        Valid values:

            * 'random': each patient is assigned a clinician randomly
from the set of clinicians.
            * 'successive': the first patient is assigned the first
clinician, the second patient is assigned the second clinician, and so
on. After the last clinician is assigned, the first clinician is
assigned again, to the next patient, repeating in a cycle.

        :rtype: str
        """
        return self._clinician_assignment
    @property
    def encounter_period(self):
        """After how many weeks does a patient have a clinical
encounter?

        :rtype: int
        """
        return self._encounter_period

    @property
    def patient_states(self):
        """What initial state is assigned to a patient?

        Valid values:

            * 'all_unaware': each patient is initially unaware
            * 'all_aware': each patient is initially aware
            * 'all': first patient is unaware, the second is aware,
third is participating, fourth contributing, fifth owning, then the
sixth is aware again, and so on, in a cycle
            * 'all_except_unware': first patient is aware, second is
participating, third contributing, fourth owning, then the fifth is
aware again, and so on, in a cycle
            * 'random_choice': each patient is assigned an initial
state among the five possible states, randomly
            * list of states: the first patient is assigned the first
state in the list, the second patient is assigned the second state in
the list, and so on, cycling as needed
```

```
            * a dictionary of states and proportions

        :rtype: str
        """
        return self._patient_states
    @property
    def clinician_states(self):
        """What initial state is assigned to a clinician?

        Valid values:

            * 'all_unaware': each clinician is initially unaware
            * 'all_aware': each clinician is initially aware
            * 'all': first clinician is unaware, the second is aware,
third is participating, fourth contributing, fifth owning, then the
sixth is aware again, and so on, in a cycle
            * 'all_except_unware': first clinician is aware, second is
participating, third contributing, fourth owning, then the fifth is
aware again, and so on, in a cycle
            * 'random_choice': each clinician is assigned an initial
state among the five possible states, randomly
            * list of states: the first clinician is assigned the
first state in the list, the second clinician is assigned the second
state in the list, and so on, cycling as needed
            * a dictionary of states and proportions

        :rtype: str
        """
        return self._clinician_states
    @property
    def encounter_aware_on(self):
        """Might a clinician cause a patient to become aware?

        :rtype: boolean
        """
        return self._encounter_aware_on
    @property
    def encounter_aware_likelihood(self):
        """Probability of clinician causing patient to become aware.

        :rtype: float
        """
        return self._encounter_aware_likelihood

    @property
    def encounter_activate_on(self):
        """Might a clinician cause a patient to activate?

        :rtype: boolean
        """
```

```
        return self._encounter_activate_on
    @property
    def patient_activate_determiner(self):
        """Specification of probabilities of patient activating.

        :rtype: TransitionDeterminer
        """
        return self._patient_activate_determiner
    @property
    def encounter_dispirit_on(self):
        """Might a clinician cause a patient to activate?

        :rtype: boolean
        """
        return self._encounter_dispirit_on
    @property
    def patient_dispirit_determiner(self):
        """Specification of probabilities of patient dispiriting.

        :rtype: TransitionDeterminer
        """
        return self._patient_dispirit_determiner

    @property
    def patient_influence_on(self):
        """Might a patient influence another patient to change states?

        :rtype: boolean
        """
        return self._patient_influence_on

    @property
    def clinician_influence_on(self):
        """Might a clinician influence another clinician to change
states?

        :rtype: boolean
        """
        return self._clinician_influence_on

    @property
    def clinician_dispirit_on(self):
        """Might a clinician become dispirited?

        :rtype: boolean
        """
        return self._clinician_dispirit_on

    @property
    def shared_knowledge_initial(self):
```

```
        """How much shared knowledge is available initially?

        :rtype: float
        """
        return self._shared_knowledge_initial
    @property
    def shared_knowledge_decays_on(self):
        """Does shared knowledge decay over time?

        :rtype: boolean
        """
        return self._shared_knowledge_decays_on

    @property
    def patient_contributes_shared_knowledge_on(self):
        """Do patients contribute shared knowledge?

        :rtype: boolean
        """
        return self._patient_contributes_shared_knowledge_on
    @property
    def patient_shared_knowledge_contrib_determiner(self):
        """How often and how much shared knowledge do patients
contribute?

        :rtype: ContributionDeterminer
        """
        return self._patient_shared_knowledge_contrib_determiner

    @property
    def clinician_contributes_shared_knowledge_on(self):
        """Do clinicians contribute shared knowledge?

        :rtype: boolean
        """
        return self._clinician_contributes_shared_knowledge_on
    @property
    def clinician_shared_knowledge_contrib_determiner(self):
        """How often and how much shared knowledge do clinicians
contribute?

        :rtype: ContributionDeterminer
        """
        return self._clinician_shared_knowledge_contrib_determiner

    @property
    def enhanced_registry_initial(self):
        """How many records are in the enhanced registry at
beginning?"""
        return self._enhanced_registry_initial
```

```
    @property
    def enhanced_registry_affects_commons_on(self):
        """Does analysis of the enhanced registry periodically incr
commons?"""
        return self._enhanced_registry_affects_commons_on
    @property
    def enhanced_registry_analysis_period(self):
        """Analysis of enhanced registry every how many weeks?"""
        return self._enhanced_registry_analysis_period
    @property
    def enhanced_registry_records_per_commons_item(self):
        """Analysis of how many EHR records adds a single item to
commons?"""
        return self._enhanced_registry_records_per_commons_item

    @property
    def phenotype_response_info_affects_praxis_on(self):
        """Does phenotype response info affect praxis?

        :rtype: boolean
        """
        return self._phenotype_response_info_affects_praxis_on
    @property
    def potential_phenotype_response_info_from_SK_unit(self):
        """Each unit of shared knowledge results in how much phenotype
res info?

        :rtype: float
        """
        return self._potential_phenotype_response_info_from_SK_unit
    @property
    def phenotype_realization(self):
        """Engagement of patients or clinicians affects phenotype res
info?

        Valid values:

            * 'only_participation_counts': if patient is at least
participating, the full extent of increase is realized. Otherwise
nothing. Clinician engagement is ignored.
            * 'patient_only': the progress of patient engagement only.
Clinician engagement is ignored.
            * 'heavily_patient': both progress of patient engagement
and progress of clinician engagement are considered, as a weighted
average, heavily weighted toward patients
            * 'slightly_patient': both progress of patient engagement
and progress of clinician engagement are considered, as a weighted
average, slightly weighted toward patient progress
            * 'even_both': both progress of patient engagement and
progress of clinician engagement are considered, and evenly weighted.
```

```
            * 'slightly_clinician': both progress of patient
engagement and progress of clinician engagement are considered, as a
weighted average, slightly weighted toward clinician progress
            * 'heavily_clinician': both progress of patient engagement
and progress of clinician engagement are considered, as a weighted
average, heavily weighted toward clinician
            * 'clinician_only': the progress of clinician engagement
only. Patient engagement is ignored.
            * 'linear_once_participating': the mean progress of
patient engagement and clinician engagement, but with no progress
noted for agents who are only aware

        Ignored if **phenotype_realization_numeric** is supplied

        :rtype: str
        """
        return self._phenotype_realization

    @property
    def phenotype_realization_numeric(self):
        return self._phenotype_realization_numeric

    @property
    def patient_response_info_affects_praxis_on(self):
        """Does patient response info affect praxis?

        :rtype: boolean
        """
        return self._patient_response_info_affects_praxis_on
    @property
    def patient_response_info_initial(self):
        """What is initial level of patient response info?

        :rtype: float
        """
        return self._patient_response_info_initial

    @property
    def patient_response_info_increase(self):
        """Engagement of patient or clinician affects patient response
info?

        Value values:

            * 'only_participation_counts': if patient is at least
participating, the full extent of increase is realized. Otherwise
nothing. Clinician engagement is ignored.
            * 'patient_only': the progress of patient engagement alone
determines increase, with an unaware patient causing no increase, and
an owning patient causing full increase. Clinician engagement is
```

```
ignored.
            * 'heavily_patient': both progress of patient engagement
and progress of clinician engagement are considered, as a weighted
average, heavily weighted toward patients
            * 'slightly_patient': both progress of patient engagement
and progress of clinician engagement are considered, as a weighted
average, slightly weighted toward patient progress
            * 'even_both': both progress of patient engagement and
progress of clinician engagement are considered, and evenly weighted.
            * 'slightly_clinician': both progress of patient
engagement and progress of clinician engagement are considered, as a
weighted average, slightly weighted toward clinician progress
            * 'heavily_clinician': both progress of patient engagement
and progress of clinician engagement are considered, as a weighted
average, heavily weighted toward clinician
            * 'clinician_only': the progress of clinician engagement
only. Patient engagement is ignored.
            * 'linear_once_participating': the mean progress of
patient engagement and clinician engagement, but with no progress
noted for agents who are only aware

        :rtype: str
        """
        return self._patient_response_info_increase

    @property
    def patient_response_info_increase_numeric(self):
        return self._patient_response_info_increase_numeric
    @property
    def shared_knowledge_affects_patient_response_info_on(self):
        """Does shared knowledge affect patient response info?

        :rtype: boolean
        """
        return self._shared_knowledge_affects_patient_response_info_on
    @property
    def patient_response_info_acceleration_from_SK_unit(self):
        """Single unit of shared knowledge accels patient response
info by?

        :rtype: float
        """
        return self._patient_response_info_acceleration_from_SK_unit
    @property
    def maximal_patient_response_info_acceleration_from_SK(self):
        """Maximal acceleration of patient response info because of
shd know?

        :rtype: float
        """
```

```
        return 
self._maximal_patient_response_info_acceleration_from_SK

    @property
    def praxis_improves_selection_efficiency_on(self):
        """Does praxis improve selection efficiency?

        :rtype: boolean
        """
        return self._praxis_improves_selection_efficiency_on
    @property
    def selection_efficiency_minimum(self):
        """How much selection efficiency for new patient in solo 
practice?

        :rtype: float
        """
        return self._selection_efficiency_minimum
    @property
    def selection_efficiency_maximum(self):
        """How much selection efficiency for full praxis?

        :rtype: float
        """
        return self._selection_efficiency_maximum
    @property
    def previsit_planning_constrains_info_on(self):
        """Does previsit planning constrain response info used in 
praxis?

        :trype: boolean
        """
        return self._previsit_planning_constrains_info_on
    @property
    def previsit_PRI_constraints(self):
        """Dictionary of previsit level and constraints on PRI 
values"""
        return self._previsit_PRI_constraints
    @property
    def praxis_improves_evaluation_accuracy_on(self):
        """Does praxis improve evaluation accuracy?

        :rtype: boolean
        """
        return self._praxis_improves_evaluation_accuracy_on
    @property
    def evaluation_accuracy_minimum_praxis(self):
        """How much evaluation accuracy for new patient in solo 
practice?
```

```
        :rtype: float
        """
        return self._evaluation_accuracy_minimum_praxis
    @property
    def evaluation_accuracy_maximum_praxis(self):
        """How much evluation accuracy for full praxis?

        :rtype: float
        """
        return self._evaluation_accuracy_maximum_praxis

    @property
    def realtime_usage_enhanced_registry_on(self):
        """Might the EHR be used in real time to obviate pre-visit
planning?"""
        return self._realtime_usage_enhanced_registry_on
    @property
    def previsit_obviation_from_ehr_record(self):
        """To what extent does a singl EHR record obviate pre-visit
planning?"""
        return self._previsit_obviation_from_ehr_record
    @property
    def activation_obviation_from_ehr_record(self):
        """To what extent does a singl EHR record obviate
activation?"""
        return self._activation_obviation_from_ehr_record

    @property
    def patient_engagement_degree(self):
        """How does the state of the patient map to engagement
level?"""
        return self._patient_engagement_degree
    @property
    def clinician_engagement_degree(self):
        """How does the state of the clinician map to engagement
level?"""
        return self._clinician_engagement_degree

    @property
    def seed(self):
        """Seed for random number generator.

        :rtype: int
        """
        return self._seed

    # the following all behave like simple static properties but are
    # in fact calculated on the fly.

    # could memoize this
```

```
    @property
    def patient_response_info_decay_prop_per_week(self):
        """By what proportion does patient response info decay weekly?

        :rtype: float
        """
        return 1 - 0.5 ** (1.0 /
self._patient_response_info_half_life)

    def shared_knowledge_decay_prop_per_week(self):
        """By what proportion does shared knowledge decline each week?

        :rtype: float
        """
        return 1 - 0.5 ** (1.0 / self._shared_knowledge_half_life)

    @functools.lru_cache(maxsize=None)
    def age_out_weekly_proportion(self):
        """What propertion of patients age out each week?

        :rtype: float
        """
        return _annual_to_weekly(self._age_out_annual_proportion)

    @functools.lru_cache(maxsize=None)
    def move_away_weekly_proportion(self):
        """What proportion of patients move away each week?

        :rtype: float
        """
        return _annual_to_weekly(self._move_away_annual_proportion)

    @functools.lru_cache(maxsize=None)
    def clinician_become_aware_weekly_prob_per_aware_infl(self):
        """What is the weekly prob per clinician influence of becoming
aware?

        :rtype: float
        """
        return _annual_to_weekly(
            self._clinician_influence_become_aware_probability
        )

    @functools.lru_cache(maxsize=None)
    def clinician_activates_weekly_prob_per_pos_influence(self):
        """What is the weekly prob per clinician influence of
activation?

        :rtype: float
        """
```

```
        return _annual_to_weekly(
            self._clinician_influence_activation_probability
        )

    @functools.lru_cache(maxsize=None)
    def clinician_dispirit_weekly_probability(self):
        """What proportion of clinicians dispirit each week?

        :rtype: float
        """
        return _annual_to_weekly(self._clinician_dispirit_probability)

    @functools.lru_cache(maxsize=None)
    def patient_become_aware_weekly_prob_per_aware_infl(self):
        """What is the weekly prob per patient influence of becoming
aware?

        :rtype: float
        """
        return _annual_to_weekly(
            self._patient_influence_become_aware_probability)

    @functools.lru_cache(maxsize=None)
    def patient_activates_weekly_prob_per_pos_influence(self):
        """What is the weekly prob per patient influence of
activation?

        :rtype: float
        """
        return
_annual_to_weekly(self._patient_influence_activation_probability)

    def serialize_to_file(self, path_prefix):
        """Write a file of all the model parameters.

        :param str path_prefix: prefix of file path that will be
written
        """
        with open('{}_model_parameters.txt'.format(path_prefix), 'w')
as f:
            def _print(param, description):
                print('{}: {}'.format(description, param), file=f)
            def _print_lf():
                print('', file=f)

            _print(self.care_centers, 'Care centers')
            _print(
                self.patients_gradual_on, 'Patients added to cohort
gradually?')
            _print(self.patients_gradual_duration,
```

```
                'Duration (in weeks) of gradual add')
            _print_lf()

            _print(self.cohort_entrance_on, 'Patients may join the
cohort?')
            _print(self.cohort_entrance_per_month,
                'How many patients join the cohort every month?')
            _print_lf()

            _print(self.cohort_exit_on, 'Patients may exit cohort?')
            _print(self._age_out_annual_proportion,
                'Annual proportion of patients who age out')
            _print(self._move_away_annual_proportion,
                'Annual proportion of patients who move away')
            _print(self.get_better_annual_proportion,
                'Annual proportion of patients with outcome 1.0 who
exit cohort')
            _print(self.get_better_start_outcome,
                'Minimum outcome level at which patient might exit
cohort')
            _print(self.get_worse_annual_proportion,
                'Annual proportion of patients with outcome 0.0 who
exit cohort')
            _print(self._get_worse_end_outcome,
                'Maximum outcome level at which patient might exit
cohort')
            _print(
                self.replace_departing_patients_on,
                'Replace departing patients?'
            )
            _print_lf()


            _print(self.phenotype_count, 'Phenotype count')
            _print(self.initial_outcome_distr, 'Initial outcome
distribution')
            _print_lf()

            _print(self.random_walk_on, 'Random walk on?')
            _print(self.random_walk_mean, 'Random walk mean')
            _print(self.random_walk_sd, 'Random walk standard
deviation')
            _print_lf()

            _print(self.relapse_on, 'Relapse on?')
            _print(self._relapse_period, 'Average relapse period
(weeks)')
            _print(self.relapse_amount, 'On relapse, outcome falls
by')
            _print_lf()
```

```
        _print(self.treatment_package_on, 'Treatment package on?')
        _print(self.treatment_package_count, 'Treatment package
count')
        _print(
            self._treatment_package_effect_mean,
            'Mean effect of effectiveness of treatment package')
        _print(
            self._treatment_package_effect_sd,
            'Standard deviation of effectivenesses of treatment
package')
        _print(
            self._best_treatment_package_effect,
            'Best treatment package effect'
        )
        _print(
            self._worst_treatment_package_effect,
            'Worst treatment package effect'
        )
        _print_lf()

        _print(self.clinician_assignment, 'Clinician assignment')
        _print(self.encounter_period, 'Encounter period (weeks)')
        _print_lf()

        _print(self.patient_states, 'Patient states')
        _print(self.clinician_states, 'Clinician states')
        _print(
            self.encounter_aware_on,
            'Encounter with clinician can cause patient to become
aware?')
        _print(
            self.encounter_aware_likelihood,
            'Encounter with clinician causes awareness how
often?')
        _print_lf()

        _print(self.encounter_activate_on, 'Patients can
activate?')
        print('Activation probabilities:', file=f)
        self.patient_activate_determiner.print(f)
        _print_lf()

        _print(self.encounter_dispirit_on, 'Patients can
dispirit?')
        print('Dispirit probabilities:', file=f)
        self.patient_dispirit_determiner.print(f)
        _print_lf()

        _print(self.patient_influence_on,
```

```
              'Patients influence other patients?')
        _print(
            self._patient_network_edges,
            'Each patient influences how many other patients?')
        _print(
            self._patient_influence_across_prop,
            'What proportion of patient influence links cross care
centers?'
        )
        _print(
            self._patient_influence_become_aware_probability,
            'Annual probability of becoming aware, per aware
influencer')
        _print(
            self._patient_influence_activation_probability,
            'Annual probability of activation, per influencer of
greater '+
            'activation')
        _print_lf()

        _print(self._clinician_influence_on,
              'Clinicians influence other clinicians?')
        _print(
            self._clinician_network_edges,
            'Each clinician influences how many other
clinicians?')
        _print(
            self._clinician_influence_across_prop,
            'What proportion of clinician infl links cross care
centers?'
        )
        _print(
            self._clinician_influence_become_aware_probability,
            'Annual probability of becoming aware, per aware
influencer')
        _print(
            self._clinician_influence_activation_probability,
            'Annual probability of activation, per influencer of
greater ' +
            'activation')
        _print_lf()

        _print(self.clinician_dispirit_on, 'Clinicians can
dispirit?')
        _print(
            self._clinician_dispirit_probability,
            'Annual probability of a clinician dispiriting')
        _print_lf()

        _print(
```

```
                self.shared_knowledge_initial,
                'Initial value for shared knowledge')
            _print(self.shared_knowledge_decays_on, 'Shared knowledge
decays?')
            _print(
                self._shared_knowledge_half_life, 'Shared knowledge
half life')
            _print_lf()

            _print(
                self._patient_contributes_shared_knowledge_on,
                'Patients contribute to shared knowledge?')
            print('Patient contribution to shared knowledge:', file=f)
            self.patient_shared_knowledge_contrib_determiner.print(f)
            _print_lf()

            _print(
                self._clinician_contributes_shared_knowledge_on,
                'Clinicians contribute to shared knowledge?')
            print('Clinicians contribution to shared knowledge:',
file=f)

self.clinician_shared_knowledge_contrib_determiner.print(f)
            _print_lf()

            _print(
                self.phenotype_response_info_affects_praxis_on,
                'Phenotype response info affects praxis?')
            _print(
                self.potential_phenotype_response_info_from_SK_unit,
                'How much does a single unit of shared knowledge
contribute '
                'to potential phenotype info?'
            )
            _print(
                self.phenotype_realization,
                'Function for combining patient engagement and
clinician '
                'engagement to determine realization of potential
phenotype '
                'response info'
            )
            _print(
                self.phenotype_realization_numeric,
                "Alternative specification of phentotype realization,
as a "
                "float from 0.0 (all clinician) to 1.0 (all patient)."
            )
            _print_lf()
```

```
            _print(
                self.patient_response_info_affects_praxis_on,
                'Patient response info affects praxis?')
            _print(
                self.patient_response_info_initial,
                'Initial value for patient response info')
            _print(
                self._patient_response_info_half_life,
                'Patient response info half life, if no increase')
            _print(
                self.patient_response_info_increase,
                'Praxis response info increase function')
            _print(
                self.patient_response_info_increase_numeric,
                'Praxis response info increase as a number')
            _print_lf()

            _print(

self.shared_knowledge_affects_patient_response_info_on,
                'Shared knowledge affects patient response info?')
            _print(
                self.patient_response_info_acceleration_from_SK_unit,
                'How much does a single unit of shared knowledge ' +
                'accelerate patient response info?')
            _print(

self.maximal_patient_response_info_acceleration_from_SK,
                'Even with much shared knowledge, what is the maximal
' +
                'patient response info acceleration?')
            _print_lf()

            _print(
                self.previsit_planning_constrains_info_on,
                'Previsit planning constrains patient response info
and '
                'phenotype response info?'
            )
            _print_lf()

            _print(
                self.praxis_improves_selection_efficiency_on,
                'Praxis improves selection efficiency?')
            _print(
                self.selection_efficiency_minimum,
                'Selection efficiency with minimal praxis')
            _print(
                self.selection_efficiency_maximum,
                'Selection efficiency with maximum praxis')
```

```
            _print_lf()

            _print(
                self.praxis_improves_evaluation_accuracy_on,
                'Praxis improves evaluation accuracy?')
            _print(
                self.evaluation_accuracy_minimum_praxis,
                'Evaluation accuracy with minimal praxis')
            _print(
                self.evaluation_accuracy_maximum_praxis,
                'Evaluation accuracy with maximal praxis')
            _print_lf()

            _print(self.patient_engagement_degree, 'Patient engagement
degree')
            _print(
                self.clinician_engagement_degree,
                'Clinician engagement degree'
            )

            _print(self.seed, 'Seed')
            _print_lf()



    def as_dict(self):
        """Return value of all parameters, as a dictionary"""
        # To do: add not-so-simple parameters, including
        # age_out_annual_proportion
        def attr_value(p):
            attr = getattr(self, f"_{p}")
            try:
                return attr.as_list()
            except AttributeError:
                return attr

        return {p: attr_value(p) for p in self.parameters}


#
# Model helper classes, one instance per model instance
#

class Clock:
    """A simple clock that keeps track of which week it is."""
    def __init__(self):
        self._current_week = 0

    @property
    def current_week(self):
```

```
        """Return the current week number.

        :rtype: int
        """
        return self._current_week

    def advance(self):
        """Advance the clock by one week."""
        self._current_week += 1


class SharedKnowledge(Stock):
    """The current level of shared knowledge, available to all agents.

    :param LearningNetworkModel model: the model that for which this
shared
        knowledge applies
    """
    def __init__(self, model):
        super().__init__(model.parameters.shared_knowledge_initial)
        self._model = model

    def change(self):
        """Change shared knowledge, as another week passed.

        Note that contributing patients or clinicians increase shared
        knowledge via increment(), so not accounted for in this
method.

        :returns: amount of shared knowledge change
        :rtype: float
        """

        if self._model.parameters.shared_knowledge_decays_on:
            amount = - self._decay()
            self._model.trace.trace_shared_knowledge_change(
                amount, self.level, 'decay')
            return amount
        else:
            return 0.0

    def increment(self, amount):
        """Increase shared knowledge from a single agent contribution.

        :param float amount: amount of shared knowledge increase.
Negative
            amount decreases.
        """
        self._level += amount
```

```
    def _decay(self):
        """How much does shared knowledge decay this week?"""
        return (
            self._level *

self._model.parameters.shared_knowledge_decay_prop_per_week())

    def add_analysis_results(self, items):
        """Increment commons by items amount of analysis results"""
        self.increment(items)
        self._model.trace.trace_shared_knowledge_change(
            items, self.level, 'enhanced_registry_analysis'
        )


class EnhancedRegistry(Stock):
    """The current number of encounters in the enhanced registry"""
    def __init__(self, model):
        params = model.parameters
        super().__init__(params.enhanced_registry_initial)
        self._model = model
        self._weeks_since_last_analysis = 0
        self._records_per_commons_item = (
            params.enhanced_registry_records_per_commons_item
        )

    def add_encounter(self):
        """Add a single encounter to the enhanced registry"""
        self.increment(1)

    @property
    def model(self):
        """What model is associated with this enhanced registry?"""
        return self._model

    @property
    def record_count(self):
        """How many records in the enhanced registry"""
        return self.level

    def step(self):
        """Another week has passed. Maybe analyze?"""
        self._maybe_analyze()

    def _maybe_analyze(self):
        """If it is time to run an analysis, analyze"""
        params = self.model.parameters
        if params.enhanced_registry_affects_commons_on:
            self._weeks_since_last_analysis += 1
            period = params.enhanced_registry_analysis_period
```

```
            if self._weeks_since_last_analysis >= period:
                self._analyze()
                self._weeks_since_last_analysis = 0

    def _analyze(self):
        """Analyze records in the enhanced registry and update
commons"""
        self.model.commons.add_analysis_results(
            self.record_count //

self.model.parameters.enhanced_registry_records_per_commons_item
        )


#
# Utility functions
#

def _annual_to_weekly(annual_prop):
    """Convert an annual proportion or probability to a weekly one."""
    return 1 - (1 - annual_prop) ** (1.0/52.0)

def _weekly_to_monthly(weekly_results):
    """Return a monthly summary of the weekly results."""

    # Note that the monthly summary is still indexed weekly
    monthly_summary = []
    days_in_month = 365.0 / 12.0
    days_elapsed_so_far = 0
    prior_months_result = None
    for result in weekly_results:
        monthly_summary.append(prior_months_result)
        days_elapsed_so_far += 7
        if days_elapsed_so_far > days_in_month:
            days_elapsed_so_far = days_elapsed_so_far - days_in_month
            prior_months_result = result
    return monthly_summary

def _weeks_per_month():
    """How many weeks per month?"""
    days_per_month = 365.25 / 12
    days_per_week = 7.0
    return days_per_month / days_per_week

# Edges

def _edge_count(node_count):
    """How many bidirectional edges given this number of nodes?"""
    return node_count * (node_count - 1) / 2
```

```
def edge_probs(counts, edge_avg, cross_prop):
    """Calc probabilities (within, across) of edge between two
agents"""
    # might could optimize via memoization
    return _edges_within_cross(*_prescribed_edges(counts, edge_avg,
cross_prop))

def _edges_within_cross(
    indicated_within, indicated_cross, potential_within,
potential_cross
):
    """Calc probabilities (within, across) given counts"""
    if potential_within > 0 and potential_cross > 0:
        return {
            "within_CC": indicated_within / potential_within,
            "cross_CC": indicated_cross / potential_cross
        }
    elif potential_within > 0:
        return {"within_CC": indicated_within / potential_within,
"cross_CC": 0}
    elif potential_cross > 0:
        return {"within_CC": 0, "cross_CC": indicated_cross /
potential_cross}
    else:
        return {"within_CC": 0, "cross_CC": 0}

def _prescribed_edges(counts, edge_avg, cross_prop):
    """counts of indicated and potential edges, within and cross"""
    total_count = sum(counts)
    indicated_total = edge_avg * total_count / 2
    potential_total = _edge_count(total_count)

    if indicated_total > potential_total:
        raise LearningNetworkModelException(
            "{} edges per agent impossible for {} agents".format(
                edge_avg, total_count))

    indicated_cross = cross_prop * indicated_total
    indicated_within = indicated_total - indicated_cross
    potential_within = sum(_edge_count(c) for c in counts)
    potential_cross = potential_total - potential_within

    if indicated_within > potential_within:
        indicated_cross += indicated_within - potential_within
        indicated_within = potential_within
    elif indicated_cross > potential_cross:
        indicated_within += indicated_cross - potential_cross
        indicated_cross = potential_cross

    return (
```

```
        indicated_within, indicated_cross, potential_within,
potential_cross
    )

def edge_probs_later(prior_counts, posterior_counts, edge_avg,
cross_prop):
    """Calc probabilities (within, across) of added edges, with prior
counts"""
    prior_ind_within, prior_ind_cross, prior_pot_within,
prior_pot_cross = (
        _prescribed_edges(prior_counts, edge_avg, cross_prop)
    )
    post_ind_within, post_ind_cross, post_pot_within, post_pot_cross =
(
        _prescribed_edges(posterior_counts, edge_avg, cross_prop)
    )
    return _edges_within_cross(
        post_ind_within - prior_ind_within,
        post_ind_cross - prior_ind_cross,
        post_pot_within - prior_pot_within,
        post_pot_cross - prior_pot_cross
    )

def softmin1(n):
    """Return the min of n and 1, softened as n approches or exceeds
1."""
    # This right side of the lofistic function is one of several
    # alternatives for a softmin, particularly appropriate if n could
be
    # a good bit more # than 1
    return 2 / (1 + math.exp(-2 * n)) - 1

#
# The history of everything that occurs in a simulation run
#

class Trace:
    """Record of everything that occurs in the simulation.

    :param Clock clock: the clock of the sim
    :param ModelParameters model_paramters: the model parameters for
the model.
        Optional: if not supplied, default model parameters are
assumed.
    """
    def __init__(self, clock, model_parameters=None, model=None):
        self._clock = clock
        self._patient_traces = []
        self._clinician_traces = []
        self._encounter_traces = []
```

```
        self._transition_traces = []
        self._treatment_package_traces = []
        self._relapse_traces = []
        self._shared_knowledge_traces = []
        self._shared_knowledge_change_traces = []
        self._cohort_change_traces = []
        self._influence_edge_traces = []
        self._model_parameters = (
            model_parameters if model_parameters else
ModelParameters()
        )
        self._model = model

    def trace_patients(self, patients):
        """Add the current statuses of the patients to sim history.

        :param patients: the patients whose statuses to be recorded.
Typically
            this is all of them, the list of all patients.
        :type patients: list of :class:`Patient`
        """
        self._patient_traces.append({p: p.current_status() for p in
patients})

    def trace_clinicians(self, clinicians):
        """Add the current statuses of the clinicians to sim history

        :param clinicians: the clinicians whose statuses to be
recorded.
            Typically this is all of them, the list of all clinicians.
        :type clinicians: list of :class:`Clinician`
        """
        self._clinician_traces.append(
            {c: c.current_status() for c in clinicians})

    def trace_encounter(self, patient, clinician):
        """Record a clinical encounter between patient and clinician.

        :param Patient patient: the patient who is having the
clinician
            encounter this week
        :param Clinician clinician: the clinician who is having the
clinical
            encounter this week with the patient
        """
        self._encounter_traces.append(
            {'week': self._current_week(), 'patient': patient,
             'clinician': clinician})

    def trace_transition(self, agent, transition, from_state,
```

```
to_state):
        """Record that a state transition occured.

        Either a patient or a clinician has transitioned from one 
state to
        another. Record the details of that transition.

        :param LearningNetworkAgent agent: the patient or clinician 
who is
            transitioning states
        :param str transition: the name of the transition, either
            'become_aware' or 'activate' or 'dispirit'
        :param str from_state: the state that the agent is leaving, 
one of
            'unaware', 'aware', 'participating', 'contributing', or 
'owning'
        :param str to_state: the state that the agent is entering, one 
of
            'unaware', 'aware', 'participating', 'contributing', or 
'owning'
        """
        self._transition_traces.append(
            {'agent': agent, 'week': self._current_week(),
             'from': from_state, 'to': to_state, 'transition': 
transition})

    def trace_treatment_package(
            self, patient, from_treatment_package, 
to_treatment_package):
        """Record that the patient's treatment package changed.

        Either the patient is previously untreated, and is now 
prescribed a
        treatment package, or the treatment is changed from one 
treatment
        package to another.

        :param Patient patient: the patient whose treatment is 
changing
        :param from_treatment_package: the prior treatment package, or 
None
            if the patient was previously untreated
        :type from_treatment_package: TreatmentPackage or None
        :param TreatmentPackage to_treatment_package: the newly 
prescribed
            treatment package for this patient
        """
        self._treatment_package_traces.append(
            {'patient': patient, 'week': self._current_week(),
             'from': from_treatment_package, 'to': 
```

```
to_treatment_package})

    def trace_relapse(self, patient, outcome_prior_to_relapse):
        """Record that the patient relapsed.

        The patient has relapsed, with a dramatic lowering of his
outcome.
        Record that change.

        :param Patient patient: patient who relapsed
        :param float outcome_prior_to_relapse: the outcome of the
patient,
            before he relapsed
        """
        self._relapse_traces.append(
            {'patient': patient, 'week': self._current_week(),
             'outcome_prior_to_relapse': outcome_prior_to_relapse})

    def trace_shared_knowledge(self, new_level):
        """Record the current state of shared knowledge.

        Every week sees a new amount of changed knowledge. Record it.

        :param float new_level: the new amount of shared knowledge
        """
        self._shared_knowledge_traces.append(
            {'week': self._current_week(), 'shared_knowledge':
new_level})

    def trace_shared_knowledge_change(self, change_amount, new_level,
agent):
        """Record that shared knowledge changes.

        Shared knowledge changes every time a patient or clinician
        contributes, and every week, with a gradual decay. Record this
        change to shared knowledge.

        :param float change_amount: the increase (or decrease, if
negative)
            in shared knowledge
        :param float new_level: the new amount of shared knowledge,
after the
            change occurs
        :param agent: who changed the shared knowledge, either a
clinician or
            a patient or the string 'decay' or the string
            'enhanced_registry_analysis'
        :type agent: :class:`Clinician` or :class:`Patient` or str
        """
        self._shared_knowledge_change_traces.append(
```

```
            {'week': self._current_week(),
             'change_amount': change_amount,
             'shared_knowledge_after_change': new_level,
             'agent_of_change': agent})

    def trace_cohort_entrance(self, patient, reason, outcome):
        """Record that a patient has entered the cohort.

        A patient enters the cohort, either one of the patients who
enrolled
        initially, or a patient who was added to the cohort later,
e.g.
        because he develops the condition. Record this addition.

        :param Patient patient: patient who joined the cohort
        :param str reason: the reason the patient joined, either
'initial' or
            'added later'
        :param float outcome: the outcome of the patient when joining
the
            cohort
        """
        self._cohort_change_traces.append(
            {'week': self._current_week(),
             'patient': patient,
             'action': 'entrance',
             'outcome': outcome,
             'reason': reason})

    def trace_cohort_exit(self, patient, reason, outcome):
        """Record that a patient has left the cohort.

        A patient leaves the cohort, either because he ages out, or
because he
        moves away, or because his condition improves to the point of
no
        longer needing treatment, or because his condition worsens and
        now requires more intensive treatment. Record this change.

        :param Patient patient: the patient who leaves the cohort
        :param str reason: the reason the patient leaves, one of 'aged
out',
            'moved away', 'got better', or 'got worse'
        :param float outcome: the outcome of the patient when exiting
        """
        self._cohort_change_traces.append(
            {'week': self._current_week(),
             'patient': patient,
             'action': 'exit',
             'outcome': outcome,
```

```
            'reason': reason})

    def trace_influence_edge(self, agent1, agent2):
        """Record an influence edge between the two agents.

        The two patients or two clinicians influence each other,
because of
        some social connection, a personal or professional
relationship.
        Record this influence.

        :param LearningNetworkAgent agent1: one of the two agents
        :param LearningNetworkAgent agent2: the other agent
        """
        self._influence_edge_traces.append(
            {
                'week': self._current_week(),
                'agent 1': agent1,
                'agent 2': agent2
            }
        )

    def patients(self):
        """Return the week-by-week patient traces as a dataframe.

        :returns: a pandas dataframe of all calls to
            :meth:`Trace.trace_patients`.
        :rtype: pandas.DataFrame

        :Example:

        >>> example_trace.patients()
            clinician   outcome     patient     perceived_outcome
praxis  state   treatment_package   week
        0   Clinician(4)    0.615198    Patient(10)     NaN     1
unaware     None    0
        1   Clinician(1)    0.695271    Patient(11)     NaN     1
aware   None    0
        2   Clinician(6)    0.570543    Patient(12)     NaN     1
participating   None    0
        3   Clinician(7)    0.909770    Patient(13)     NaN     1
contributing    None    0
        4   Clinician(1)    0.915555    Patient(14)     NaN     1
owning  None    0
        5   Clinician(2)    0.435628    Patient(15)     NaN     1
unaware     None    0
        6   Clinician(4)    0.961298    Patient(16)     NaN     1
aware   None    0
        7   Clinician(8)    0.239337    Patient(17)     NaN     1
participating   None    0
```

```
        ...

        """
        return pd.DataFrame(
            [{**{'week': week, 'patient': patient}, **pvalues}
             for week_values, week in zip(
                self._patient_traces, itertools.count(0))
             for patient, pvalues in week_values.items() ])

    def clinicians(self):
        """Return the week-by-week clinician traces as a dataframe.

        :returns: a pandas dataframe of all calls to
            :meth:`Trace.trace_clinicians`
        :rtype: pandas.DataFrame

        :Example:

        >>> example_trace.clinicians()
            clinician   state   week
        0   Clinician(0)    aware   0
        1   Clinician(1)    aware   0
        2   Clinician(2)    aware   0
        3   Clinician(3)    aware   0
        4   Clinician(4)    aware   0
        5   Clinician(5)    aware   0
        6   Clinician(6)    aware   0
        7   Clinician(7)    aware   0
        ...

        """
        return pd.DataFrame(
            [{**{'week': week, 'clinician': clinician}, **cvalues}
             for week_values, week in zip(
                self._clinician_traces, itertools.count(0))
             for clinician, cvalues in week_values.items()])

    def encounters(self):
        """Return the encounters as a dataframe.

        :returns: a pandas dataframe of all calls to
            :meth:`Trace.trace_encounter`
        :rtype: pandas.DataFrame

        :Example:

        >>> example_trace.encounters()
            clinician   patient     week
        0   Clinician(4)    Patient(120)    10
        1   Clinician(6)    Patient(172)    10
```

```
        2   Clinician(3)    Patient(90)     10
        3   Clinician(9)    Patient(184)    10
        4   Clinician(7)    Patient(37)     10
        5   Clinician(4)    Patient(185)    10
        6   Clinician(2)    Patient(198)    10
        7   Clinician(6)    Patient(137)    10
        ...

        """
        return pd.DataFrame(self._encounter_traces)

    def transitions(self):
        """Return the transitions as a dataframe.

        :returns: a pandas dataframe of all calls to
            :meth:`Trace.trace_transition`
        :rtype: pandas.DataFrame

        :Example:

        >>> example_trace.transition()
            agent   from    to  transition  week
        0   Patient(45)     unaware     aware   become_aware    4
        1   Patient(20)     unaware     aware   become_aware    5
        2   Patient(90)     unaware     aware   become_aware    6
        3   Patient(25)     unaware     aware   become_aware    9
        4   Patient(85)     unaware     aware   become_aware    10
        ...

        """
        return pd.DataFrame(self._transition_traces)

    def treatment_packages(self):
        """Return the treatment packages as a dataframe.

        :returns: a pandas dataframe of all calls to
            :meth:`Trace.trace_treatment_package`
        :rtype: pandas.DataFrame

        :Example:

        >>> example_trace.treatment_packages()
            from    patient     to  week
        0   none    Patient(120)    TreatmentPackage(1)     10
        1   none    Patient(172)    TreatmentPackage(3)     10
        2   none    Patient(90)     TreatmentPackage(3)     10
        3   none    Patient(184)    TreatmentPackage(3)     10
        4   none    Patient(37)     TreatmentPackage(7)     10
        5   none    Patient(185)    TreatmentPackage(2)     10
        6   none    Patient(198)    TreatmentPackage(3)     10
```

```
    7   none    Patient(137)    TreatmentPackage(3)     10
    ...

    """
    return pd.DataFrame(self._treatment_package_traces)

def relapses(self):
    """Return the relapses as a dataframe.

    :returns: a pandas dataframe of all calls to
        :meth:`Trace.trace_relapse`
    :rtype: pandas.DataFrame

    :Example:

    >>> example_trace.relapses()
        outcome_prior_to_relapse    patient     week
    0   0.573608    Patient(166)    1
    1   0.270561    Patient(169)    1
    2   0.102705    Patient(203)    1
    3   0.345142    Patient(160)    1
    4   0.958036    Patient(93)     1
    5   0.071380    Patient(136)    1
    6   0.433286    Patient(64)     1

    """
    return pd.DataFrame(self._relapse_traces)

def shared_knowledge(self):
    """Return the shared knowledge as a dataframe.

    :returns: a pandas dataframe of all calls to
        :meth:`Trace.trace_shared_knowledge`
    :rtype: pandas.DataFrame

    :Example:

    >>> example_trace.shared_knowledge()
        shared_knowledge    week
    0   0.0     0
    1   4.0     1
    2   9.5     2
    3   15.0    3
    4   20.5    4
    5   22.5    5
    6   28.0    6

    """
    return pd.DataFrame(self._shared_knowledge_traces)
```

```
    def shared_knowledge_changes(self):
        """Return the shared knowledge changes as a dataframe.

        :returns: a pandas dataframe of all calls to
            :meth:`Trace.trace_shared_knowledge_change`
        :rtype: pandas.DataFrame

        :Example:

        >>> example_trace.shared_knowledge_changes()
            agent_of_change     change_amount
shared_knowledge_after_change   week
        0   Patient(18)     1.0     1.0     1
        1   Patient(202)    0.5     1.5     1
        2   Patient(169)    1.5     3.0     1
        3   Patient(128)    1.0     4.0     1
        4   Patient(163)    1.0     5.0     2
        5   Patient(22)     0.5     5.5     2
        6   Patient(119)    1.5     7.0     2
        7   Patient(108)    1.0     8.0     2

        """
        return pd.DataFrame(self._shared_knowledge_change_traces)

    def cohort_changes(self):
        """Return the cohort changes as a dataframe.

        :returns: a pandas dataframe of all calls to
            :meth:`Trace.trace_cohort_entrance` and
            :meth:`Trace.trace_cohort_exit`
        :rtype: pandas.DataFrame

        :Example:

        >>> example_trace.cohort_changes()
            action  outcome     patient     reason  week
        0   entrance    0.686102    Patient(10)     initial     0
        1   entrance    0.513291    Patient(11)     initial     0
        2   entrance    0.884145    Patient(12)     initial     0
        3   entrance    0.617901    Patient(13)     initial     0
        4   entrance    0.884346    Patient(14)     initial     0
        5   entrance    0.921991    Patient(15)     initial     0
        6   entrance    0.819285    Patient(16)     initial     0
        7   entrance    0.085391    Patient(17)     initial     0

        """
        return pd.DataFrame(self._cohort_change_traces)

    def influence_edges(self):
        """Return all the edges between agents, as a dataframe.
```

```
        :returns: a pandas dataframe of all calls to
            :meth:`Trace.trace_influence_edge`
        :rtype: pandas.DataFrame

        :Example:

        >>> example_trace.influence_edges()
            agent 1     agent 2
        0   Patient(18)     Patient(14)
        1   Patient(24)     Patient(22)
        2   Patient(30)     Patient(29)
        3   Patient(32)     Patient(19)
        4   Patient(32)     Patient(28)
        5   Patient(35)     Patient(15)
        6   Patient(36)     Patient(26)
        7   Patient(37)     Patient(23)

        """
        return pd.DataFrame(self._influence_edge_traces)

    def count_patient_transition_of(self, transition_sought):
        """Count the number of transitions of some transition type.

        How many activate transitions  have been recorded? Or how many
        dispirits?

        :param str transition_sought: either 'activate' or 'dispirit'
or
            'become_aware'
        :returns: the count of this type of transition
        :rtype: int
        """
        ts = self.transitions()
        if ts.empty:
            return 0
        else:
            return sum(ts['transition']== transition_sought)

    def patients_by_clinician(self, clinician, week):
        """Return list of patients for this clinician in week.

        Which patients are treated by this clinician, on this week?
Note
        that this is not just the patients who have clinical
encounters
        with this clinician on this week, but all the patients who are
        under her care.

        :param Clinician clinician: the clinician whose patients are
```

```
sought
        :param int week: the week number that the clinician is
responsible
            for the patients
        :returns: patients who are under the care of this clinician
        :rtype: list of :class:`Patient`

        """
        return [p for p, pvalues in self._patient_traces[week].items()
                  if pvalues['clinician'] == clinician]

    def patient_display_results(self):
        """Return the patient history in a display format.

        Used by user interfaces to retrieve the results of patient
outcomes
        week-by-week, for display to users. The format is a nested
dict.
        The outer dict is by patients, keyed by patient name. For each
patient,
        there is a dict of care center, patient condition by week, and
praxis
        by week. The by week values are both lists.

        :returns: dict of patient results, suitable for display.
        :rtype: dict

        :Example:

        >>> example_trace.patient_display_results()
        {
            'Patient(11)': {
                "care_center": 4,
                "condition": [0.6982027756386627, 0.7099504139436428,
0.7052466763163927, 0.7120904073352328, 0.7145846686455587],
                "praxis": [0.1, 0.2, 0.3, 0.4, 0.5],
            },
            'Patient(54)': {
                "care_center": 5,
                "condition": [None, None, 0.46692491566760674,
0.4788947289892505, 0.48575758881462905]],
                "praxis": [None, None, 0.3, 0.4, 0.5],
            },
            'Patient(97)': {
                "care_center": 1,
                "condition": [0.5592375593714402, 0.5635922948400581,
0.5602376120466876, 0.070011320878679, None],
                "praxis": [0.1, 0.2, 0.3, 0.4, None],
            },
            ....
```

```
        }
        """
        patients = self._find_all_patients()
        return {
            str(patient): {
                "care_center": patient.care_center.id,
                "condition": [
                    self._outcome_or_none(week, patient)
                    for week in self._patient_traces
                ],
                "praxis": [
                    self._praxis_or_none(week, patient)
                    for week in self._patient_traces
                ]
            }
            for patient in patients
        }

    def _find_all_patients(self):
        """Return all patients, including those that came and went."""
        patients = set()
        for week in self._patient_traces:
            for patient in week.keys():
                patients.add(patient)
        return list(patients)

    def _outcome_or_none(self, week_results, patient):
        """Return patients's outcome in week_results, or None if not
there."""
        try:
            return week_results[patient]['outcome']
        except KeyError:
            return None

    def _praxis_or_none(self, week_results, patient):
        """Return patients's outcome in week_results, or None if not
there."""
        try:
            return week_results[patient]['praxis']
        except KeyError:
            return None

    def patient_aggregate_display_results(self):
        """Return medians and other aggregates of the history in disp
format.

        Used by user interfaces to retrieve various aggregates of
patient
        data week-by-week, e.g. median, praxes, etc. The praxis
aggregate
```

```
        finds the minimum value of praxis across all patients, the 25
percentile
        value, the median, the 75 percentile, and the maximum,
assembling these
        five quantiles into a dict.

        :returns: a nested dict. The outer dict has keys for each care
center ID
            (as a sring) plus "all". The value of each is a dict, with
keys for
            the different types of aggregations:
            'median_outcomes', 'median_perceived_outcomes',
            'median_reported_outcomes', and 'praxes'. Each value in
the dict
            is a list of the week-by-week numeric aggregation, except
for
            praxis, which is a list of dicts.
        :rtype: dict
        """
        median_outcomes = self.median_outcomes()
        median_perceived_outcomes = self.median_perceived_outcomes()
        median_reported_outcomes = {
            cc: _weekly_to_monthly(outcomes)
            for cc, outcomes in median_perceived_outcomes.items()
        }
        praxes = self.aggregate_praxes()
        return {
            cc: {
                'median_outcomes': median_outcomes[cc],
                'median_perceived_outcomes':
median_perceived_outcomes[cc],
                'median_reported_outcomes':
median_reported_outcomes[cc],
                'praxes': praxes[cc]
            }
            for cc in median_outcomes.keys()
        }

    def median_outcomes(self):
        """Return the median outcomes, as a dict of lists.

        Used by user interfaces to retrieve median outcomes, across
patients.

        :returns: dict of list of median outcomes. Each key of the
dict is
            either a care center (string) or 'all'. Each value is a
list of
            median outcomes, for a particular week
        :rtype: list
```

```
        """
        return self._weekly_stats_by_care_center(stats.median, 0,
'outcome')

    def _weekly_stats_by_care_center(self, fn, empty_val, attr):
        """Apply a function to patient stats. Return by care
center."""
        alls = [
            fn([pvalues[attr] for pvalues in patient_week.values()])
            for patient_week in self._patient_traces
        ]
        ccs = {
            str(cc): [
                self._apply_not_empty(
                    fn,
                    empty_val,
                    [
                        pvalues[attr]
                        for patient, pvalues in patient_week.items()
                        if patient.care_center.id == cc
                    ]
                )
                for patient_week in self._patient_traces
            ]
            for cc in self._care_center_ids()
        }
        ccs['all'] = alls
        return ccs

    @staticmethod
    def _apply_not_empty(fn, empty_val, vals):
        """Apply fn to empty vals unless vals is empty"""
        if vals:
            return fn(vals)
        else:
            return empty_val

    def _care_center_ids(self):
        """Return list of IDs for all care centers."""
        return [cc.id for cc in self._model._care_centers]

    def median_perceived_outcomes(self):
        """Return the median perceived outcomes, as a dict of
lists."""
        def _median(patient_week, id=None):
            """Find the median perceived outcome of all patients in
week."""
            perceived_outcomes = [
                pvalues['perceived_outcome']
                for patient, pvalues in patient_week.items()
```

```
                if 'perceived_outcome' in pvalues and (
                    id is None or patient.care_center.id == id
                )
            ]
            if perceived_outcomes:
                return stats.median(perceived_outcomes)
            else:
                return None

        alls = [_median(patient_week) for patient_week in
self._patient_traces]
        ccs = {
            str(cc): [
                _median(patient_week, cc)
                for patient_week in self._patient_traces
            ]
            for cc in self._care_center_ids()
        }
        ccs['all'] = alls
        return ccs

    def aggregate_praxes(self):
        """Return box and whisker data for the praxis, for each
week."""
        def _box_and_whiskers(vals):
            """Return min, 1 quartile, median, 3 quartile, max of
vals."""
            npvals = np.array(vals)
            q1, q2, q3 = np.percentile(npvals, [25, 50, 75])
            return {
                'min': npvals.min().item(), 'q1': q1, 'q2': q2, 'q3':
q3,
                'max': npvals.max().item()}

        return self._weekly_stats_by_care_center(
            _box_and_whiskers,
            {'min': 0, 'q1': 0, 'q2':0, 'q3': 0, 'max': 0},
            'praxis'
        )

    def count_patients_in_state(self, week, state):
        """Count patients in state at week.

        How many patients are in this state on this week?

        :param int week: the week to count patients
        :param str state: the state to look for, one of
            'unaware', 'aware', 'participating', 'contributing', or
'owning'
        :returns: the count of patients who are in that state
```

```
        :rtype: int
        """
        return sum(1 for pvalues in
self._patient_traces[week].values()
                     if pvalues['state'] == state)

    def count_patients_in_states_over_time(self):
        """Return count of patients in each state, on each week.

        How many patients are in each state, for each week of the
simulation?

        :returns: a dict of a list over time. The dict is indexed by
care
            center and 'all'. Each element of the list is a dict keyed
            by state, with values of the number of patients in that
state on
            that week
        :rtype: dict of list of dicts
        """
        return
self._count_agents_in_states_over_time(self._patient_traces)

    def count_clinicians_in_states_over_time(self):
        """Return count of clinicians in each state, on each week.

        How many clinicians are in each state, for each week of the
simulation?

        :returns: a dict of a list over time. The dict is indexed by
care
            center and 'all'. Each element of the list is a dict keyed
            by state, with values of the number of clinicians in that
state
            on that week
        :rtype: dict of list of dicts
        """
        return
self._count_agents_in_states_over_time(self._clinician_traces)

    def _count_agents_in_states_over_time(self, trace):
        """Return counts of agents, in dict keyed by care center"""
        alls = self._count_agents_in_states_over_time_cc(trace, 'all')
        ccs = {
            str(cc): self._count_agents_in_states_over_time_cc(trace,
cc)
            for cc in self._care_center_ids()
        }
        ccs ['all'] = alls
        return ccs
```

```
    def _count_agents_in_states_over_time_cc(self, traces, cc):
        """Return count of agents in each state, on each week."""
        states = EngagementLadder.states()
        state_counts = [{st: 0 for st in states} for wk in traces]
        for weekly_data, weekly_state_count in zip(traces,
state_counts):
            for patient, pvalues in weekly_data.items():
                if cc == 'all' or patient.care_center.id == cc:
                    weekly_state_count[pvalues['state']] += 1
        return state_counts

    def shared_knowledge_display_results(self):
        """Return shared knowledge over time, in a form suitable for
display.

        :returns: the amount of shared knowledge, by week, for each
week in
            the simulation
        :rtype: list
        """
        return [week_results['shared_knowledge']
                for week_results in self._shared_knowledge_traces]

    def cumulative_average_change_in_condition(self):
        """Determine cumulative average change in patient condition
over sim"""
        # to do: DRY this with similar logic in
        # learning_networks_app._filled_cumulative_change()
        patient_results = self.patient_display_results()
        conds = self._create_patient_array(patient_results)
        cond_delta = self._weekly_change(conds)
        return np.nanmean(cond_delta, axis=0).sum()

    @staticmethod
    def _create_patient_array(patient_results, index="condition"):
        """Create condition array, translating Nones to NANs"""
        return np.array(
            [pvalues[index] for pvalues in patient_results.values()],
            dtype=float
        )

    @staticmethod
    def _weekly_change(conds):
        """Find the weekly change in condition for each patient"""
        return np.diff(conds)

    def cumulative_average_change_in_praxis(self):
        """Determine cumulative average change in praxis over sim"""
        # to do: DRY this with similar logic in learning_networks_app
```

```
        patient_results = self.patient_display_results()
        praxes = self._create_patient_array(patient_results,
index='praxis')
        praxis_delta = self._weekly_change(praxes)
        return np.nanmean(praxis_delta, axis=0).sum()

    def cumulative_increase_in_commons(self):
        """Determine cumulative increase in commons over sim"""
        # to do: DRY this one too
        commons = self.shared_knowledge_display_results()
        return int(commons[-1] - commons[0])

    def serialize_to_files(self, path_prefix):      # pragma: no cover
        """Write the various trace tables as CSV files.

        Serialize the complete history of the simulation to 10 files.
        Also serialize all model parameters, to its own file.

        :param str path_prefix: prefix of file path that will be
written
        """
        self._serialize_to_file(self.patients, path_prefix,
'patients')
        self._serialize_to_file(self.clinicians, path_prefix,
'clinicians')
        self._serialize_to_file(self.transitions, path_prefix,
'transitions')
        self._serialize_to_file(self.encounters, path_prefix,
'encounters')
        self._serialize_to_file(
            self.treatment_packages, path_prefix,
'treatment_packages')
        self._serialize_to_file(self.relapses, path_prefix,
'relapses')
        self._serialize_to_file(
            self.shared_knowledge, path_prefix, 'shared_knowledge')
        self._serialize_to_file(
            self.shared_knowledge_changes, path_prefix,
            'shared_knowledge_changes')
        self._serialize_to_file(
            self.cohort_changes, path_prefix, 'cohort_changes')
        self._serialize_to_file(
            self.influence_edges, path_prefix, 'influence_edges')
        self._model_parameters.serialize_to_file(path_prefix)

    def _serialize_to_file(self, table_generator, path_prefix,
table_name):
        """Write a single trace table as a CSV file."""
        table_generator().to_csv(      # pragma: no cover
            '{}_{}.csv'.format(path_prefix, table_name), index=False)
```

```
    def _current_week(self):
        """What is the week right now?"""
        return self._clock.current_week

class LearningNetworkModelException(Exception):
    """An error for some problem in the learning model."""
    def __init__(self, message):
        self.message = message
```
