# Supplementary material for: An agent-based model to advance the science of collaborative learning health systems
Source: PLoS One. 2025 Sep 9;20(9):e0332054. doi: 10.1371/journal.pone.0332054 (PMC12419628; doi:10.1371/journal.pone.0332054)
Supplement: S4 Supplement — (PDF) [file pone.0332054.s004.pdf]

R code for sensitivity analysis

```
rm(list=ls(all.names = TRUE))

library("sensitivity")
library("ggplot2")
library("dplyr")
library("forcats")
library("officer")
library("flextable")

# https://search.r-project.org/CRAN/refmans/sensitivity/html/pcc.html

dat_df <- data.frame(read.csv("c:/Users/david/Dropbox/Data/clusters/39/results.csv",
na.strings="NA"))

the_variables <- c("encounter_period",
           "encounter_aware_likelihood",
           "patient_activate_determiner",
           "patient_dispirit_determiner",
           "patient_network_edges",
           "patient_influence_across_prop",
           "patient_influence_become_aware_probability",
```

```
"patient_influence_activation_probability",
"clinician_network_edges",
"clinician_influence_across_prop",
"clinician_influence_become_aware_probability",
"clinician_influence_activation_probability",
"clinician_dispirit_probability",
"shared_knowledge_initial",
"shared_knowledge_half_life",
"patient_shared_knowledge_contrib_determiner",
"clinician_shared_knowledge_contrib_determiner",
"enhanced_registry_initial_per_patient",
"enhanced_registry_analysis_period",
"enhanced_registry_records_per_commons_item",
"potential_phenotype_response_info_from_SK_unit",
"phenotype_realization_numeric",
"patient_response_info_half_life",
"patient_response_info_increase_numeric",
"patient_response_info_acceleration_from_SK_unit",
"maximal_patient_response_info_acceleration_from_SK",
"selection_efficiency_maximum",
"evaluation_accuracy_minimum_praxis",
"patient_engagement_degree_participating",
"clinician_engagement_degree_participating"
)

the_outcomes <- c("median_patient_health_condition", #1
```

```
        "patient_unaware", #2
        "patient_aware", #3
        "patient_participating", #4
        "patient_contributing", #5
        "patient_owning", #6
        "clinician_unaware", #7
        "clinician_aware", #8
        "clinician_participating", #9
        "clinician_contributing", #10
        "clinician_owning", #11
        "commons", #12
        "cumulative_average_change_in_health", #13
        "cumulative_average_change_in_praxis", #14
        "cumulative_increase_in_commons" #15
        )

xx <- dat_df[c(the_variables)]

the_outcome <- 15

if(the_outcome == 1){yy <- c(dat_df$median_patient_health_condition)
title_text <- "median_patient_health_condition"}

if(the_outcome == 2){yy <- c(dat_df$patient_unaware)
title_text <- "patient_unaware"}
```

```
if(the_outcome == 3){yy <- c(dat_df$patient_aware)

title_text <- "patient_aware"}


if(the_outcome == 4){yy <- c(dat_df$patient_participating)

title_text <- "patient_participating"}


if(the_outcome == 5){yy <- c(dat_df$patient_contributing)

title_text <- "patient_contributing"}


if(the_outcome == 6){yy <- c(dat_df$patient_owning)

title_text <- "patient_owning"}


if(the_outcome == 7){yy <- c(dat_df$clinician_unaware)

title_text <- "clinician_unaware"}


if(the_outcome == 8){yy <- c(dat_df$clinician_aware)

title_text <- "clinician_aware"}


if(the_outcome == 9){yy <- c(dat_df$clinician_participating)

title_text <- "clinician_participating"}


if(the_outcome == 10){yy <- c(dat_df$clinician_contributing)

title_text <- "clinician_contributing"}


if(the_outcome == 11){yy <- c(dat_df$clinician_owning)

title_text <- "clinician_owning"}
```

```
if(the_outcome == 12){yy <- c(dat_df$commons)
title_text <- "commons"}

if(the_outcome == 13){yy <- c(dat_df$cumulative_average_change_in_health)
title_text <- "cumulative_average_change_in_health"}

if(the_outcome == 14){yy <- c(dat_df$cumulative_average_change_in_praxis)
title_text <- "cumulative_average_change_in_praxis"}

if(the_outcome == 15){yy <- c(dat_df$cumulative_increase_in_commons)
title_text <- "cumulative_increase_in_commons"}

# Bonferroni Corrections
alpha <- 0.05
adj_conf <- 1 - alpha / length(the_variables)

the_new_prccs <- pcc(xx,
        yy,
        rank = TRUE,
        semi = FALSE,
        logistic = FALSE,
        nboot = 100,
        #conf = 0.95)
        conf=adj_conf)
```

```
print(the_new_prccs)
temp_df <- as.data.frame(the_new_prccs$PRCC)
print(max(temp_df$original))
print(min(temp_df$original))

obi <- data.frame(the_variables,
        the_new_prccs$PRCC$original,
        the_new_prccs$PRCC$`std. error`,
        the_new_prccs$PRCC$`min. c.i.`,
        the_new_prccs$PRCC$`max. c.i.`)
colnames(obi) <- c("variable",
         "prcc",
         "std_error",
         "lower_ci",
         "upper_ci")

title_dummy <- paste(title_text, ", N=", length(yy), " replicates", sep="")

luke <- obi %>%
  mutate(variable = fct_reorder(variable, prcc)) %>%
  ggplot(aes(x=variable, y=prcc)) +
  geom_bar(stat="identity", fill="#f68060", alpha=.6, width=.4) +
  geom_errorbar(aes(ymin = lower_ci, ymax = upper_ci),
        width = .2, linewidth = .6, color = "black") +
  labs(x = "", y = "PRCC", title = title_dummy) +
  geom_hline(yintercept = 0, linetype = "dashed") +
```

```
  ggtitle(title_dummy) +
  coord_flip(ylim = c(-1, 1)) +
  xlab("") +
  theme(panel.background = element_rect(fill = 'white', color = 'white'),
        panel.grid.major = element_line(color = 'lightgray', linetype = 'solid'),
        axis.text=element_text(size=14),
        axis.title=element_text(size=16, face="bold"),
        plot.title = element_text(size = 18, face = "bold"))

print(luke)
```
