# Supplementary material for: An agent-based model to advance the science of collaborative learning health systems
Source: PLoS One. 2025 Sep 9;20(9):e0332054. doi: 10.1371/journal.pone.0332054 (PMC12419628; doi:10.1371/journal.pone.0332054)
Supplement: Attachment — Submitted filename: PONE-D-25-08428_R1.docx [file pone.0332054.s006.docx]

PONE-D-25-08428_R1

An agent-based model to advance the science of collaborative learning health systems

1. If the authors have adequately addressed your comments raised in a previous round of review and you feel that this manuscript is now acceptable for publication, you may indicate that here to bypass the “Comments to the Author” section, enter your conflict of interest statement in the “Confidential to Editor” section, and submit your "Accept" recommendation.
   N/A
2. Is the manuscript technically sound, and do the data support the conclusions?

Partly

3. Has the statistical analysis been performed appropriately and rigorously?
   Yes
4. Have the authors made all data underlying the findings in their manuscript fully available?
   Yes
5. Is the manuscript presented in an intelligible fashion and written in standard English?
   Yes
6. Review Comments to the Author

The manuscript used an agent-based model (ABM) to simulate Learning Health Networks (LHNs) and demonstrate their applicable advantages within the healthcare space by quantifying heuristic theory. Their manuscript describes the process of constructing an ABM, applying a sensitivity analysis to identify the most influential components within it, and creating a simulated experiment based on these components. The authors acknowledge that this model is a first step of more advanced LHN ABMs, with limitations that accompany this nascent research field. The manuscript is well-written and well-organized, and the authors have made the original code of their model easily available as a supplement.

# Major Comments

Line 384: Does this ABM operate under a single iteration, or produce summary results from multiple iterations? The mention of replicates in the sensitivity analysis implies the latter, but I believe the manuscript would be strengthened with an explicit clarification. Additionally, if multiple iterations were used, a convergence study to demonstrate that the number of iterations used is sufficiently stable would be highly beneficial.

Line 532: While this section does mention the figure with relevant data, the text does not describe it in any way. Even if the simulated experiment’s results are largely repetitive, they should be nonetheless described in the manuscript so that the discussion of Figure 5 has something to refer to.

Lines 325/498/503/526/538: While all figures are present and readable, most of them lack a descriptive figure legend to make them independently understandable.

Line 436: Please add an R/Rmd file to the submission that contains the code used for this analysis and other components of the manuscript that used R.

Line 453: Please add a table of the factorial experiment’s parameters to simplify explaining how low/medium/high levels specifically differ from each other.

Figure 2A-C: Is it possible to provide p-values of the sensitivity analysis? The authors should also consider if it is appropriate to apply a Bonferroni Correction to this analysis.

Figure 4: Please increase the grid tick/grid line intervals to make it easier to find specific coordinates mentioned in the manuscript.

Figure 5: Figure 5 shows some blurriness, but it is unclear if that is simply due to the review PDF compressing it or if Figure 5 needs to be uploaded in a higher resolution.

# Minor Comments

Line 163: Please state the language and version used to create the model in the manuscript, as well as packages used.

Line 209/210: double-bracketing is slightly jarring to read; can the sentence be restructured at all to only need a single set?

Line 587/589: This sentence should end in a question mark.

Line 690/711: I advise giving the limitations and future studies sections their own sub-header to better structure the discussions section.

Figure 5: The practicality of using Excel to create bar-charts quickly is entirely understandable, but if you have time available I would recommend recreating them in same tool as Figure 3/4 for visual consistency.
