# Supplementary material for: An agent-based model to advance the science of collaborative learning health systems
Source: PLoS One. 2025 Sep 9;20(9):e0332054. doi: 10.1371/journal.pone.0332054 (PMC12419628; doi:10.1371/journal.pone.0332054)
Supplement: Attachment — Submitted filename: PONE-D-25-08428_R2 Response Letter.docx [file pone.0332054.s008.docx]

Response to Reviewers

PONE-D-25-08428_R2

Dear Dr. Rao;

Thank you and thanks to the reviewers for these suggestions to improve “An agent-based model to advance the science of collaborative learning health systems.” Below, we detail our responses to the specific critiques.

Reviewer 1:

Line 384: Does this ABM operate under a single iteration, or produce summary results from multiple iterations? The mention of replicates in the sensitivity analysis implies the latter, but I believe the manuscript would be strengthened with an explicit clarification.

- The model incorporates stochasticity at each timestep, making it necessary to compute averages and ensembles of model runs. The replicates in the sensitivity analysis relate partially to this stochasticity and partially to ensuring adequate sampling of the distributions of the varying parameters. We have clarified this beginning on line 301: “Incorporating stochasticity at each time-step of the model reflects the role of chance in actual systems and makes it necessary to analyze ensembles of model runs.”

Additionally, if multiple iterations were used, a convergence study to demonstrate that the number of iterations used is sufficiently stable would be highly beneficial.

- Thank you for pointing out this important omission. We adjusted the number of replicates in the sensitivity analyses based on stability and statistical significance of the resulting PRCCs, finding that the confidence intervals and point estimates of the PRCCs became static after 1000 replicates. We have clarified this beginning on line 473: “Trials demonstrated PRCC stability for 800-900 replicates; we used 1000 replicates for the results described below.”.

Line 532: While this section does mention the figure with relevant data, the text does not describe it in any way. Even if the simulated experiment’s results are largely repetitive, they should be nonetheless described in the manuscript so that the discussion of Figure 5 has something to refer to.

- We have added text, beginning on line 609, to describe Fig 5a, b, and c.

Lines 325/498/503/526/538: While all figures are present and readable, most of them lack a descriptive figure legend to make them independently understandable.

- We have added descriptive figure legends to Fig 2, Fig 3, Fig 4, and Fig5.

Line 436: Please add an R/Rmd file to the submission that contains the code used for this analysis and other components of the manuscript that used R.

- We have included the snippet of R code used to generate the PRCCs and corresponding CIs in the Supplementary Materials (ABM of CLHS Supplement 4 R code for senstitivity analysis.pdf).

Line 453: Please add a table of the factorial experiment’s parameters to simplify explaining how low/medium/high levels specifically differ from each other.

- This is now Table 2, starting line 515.

| Parameter | Low | Medium | High |
| --- | --- | --- | --- |
| LHN Functioning (settings for selection efficiency and evaluation accuracy that result in LHN effect on health status of…) | sel eff: 10  eval acc: 0.001 | sel eff: 100  eval acc: 0.01 | sel eff: 1000  eval acc: 0.01 |
| Pre-visit Planning (the maximum possible effective individual response information and the phenotype response information) | 0.2 | 0.5 | 1.0 |
| Enhanced Registry (an ordinal scale denoting on/off for automated data upload and real-time PVP) | No automated data upload | Automated upload but no real-time PVP | Automated upload and real-time PVP |

Figure 2A-C: Is it possible to provide p-values of the sensitivity analysis? The authors should also consider if it is appropriate to apply a Bonferroni Correction to this analysis.

- Using the pcc function in the R sensitivity library, we computed the PRCC 95% confidence intervals based on 100 bootstrap replicates, applying Bonferroni correction. We have updated Figure 2 and added the following text to the Figure 2 caption: “Bonferroni-corrected 95% confidence intervals computed using 100 bootstrap replicates.”.

Figure 4: Please increase the grid tick/grid line intervals to make it easier to find specific coordinates mentioned in the manuscript.

- We have added additional tick marks and gridlines, and cleaned up related axis properties, to improve readability.

Figure 5: Figure 5 shows some blurriness, but it is unclear if that is simply due to the review PDF compressing it or if Figure 5 needs to be uploaded in a higher resolution.

- We have re-created this figure to improve readability.

Line 163: Please state the language and version used to create the model in the manuscript, as well as packages used.

- We have added the following to the Methods section beginning on line 415: “The model was written in Python version 3.10 utilizing the following libraries: pandas (version 1.4.1), scipy (1.9.0), transitions (0.8.11), mesa (0.9.0), flask (2.1.3), flask-restful (0.3.9), and filelock (3.8.0). The PRCC analysis was performed using R, version 4.5.1 utilizing the sensitivity library.”

Line 209/210: double-bracketing is slightly jarring to read; can the sentence be restructured at all to only need a single set?

- This sentence now reads “…better coproduction [11] (in the ABM, praxis, explained below) between…”

Line 587/589: This sentence should end in a question mark.

- This sentence now ends in a question mark.

Line 690/711: I advise giving the limitations and future studies sections their own sub-header to better structure the discussions section.

- We added these sub-headings

Figure 5: The practicality of using Excel to create bar-charts quickly is entirely understandable, but if you have time available I would recommend recreating them in same tool as Figure 3/4 for visual consistency.

- We have re-created this figure to improve readability.

Reviewer 2:

The manuscript presents better following incorporation of reviewer feedback and will be a useful resource for health service planners. However, there are yet some areas which could be improved/refined.

More details/rationale on the selection of the 30 parameters would be helpful.

- We have added text regarding the rationale for developing the set of parameters described in Table 1. (Line 381) “We attempted to design parameters that could parsimoniously describe variation in LHNs relevant to the heuristic theory of the chronic care model and the actor-oriented organizational architecture. These 30 parameters are only a subset of all the possible influences on an LHN or of all the ways that an LHN could vary. We did not include variables characterizing broader macro-systems, such as cost-effectiveness population demographics, or payor mix.”
- We describe the selection criteria for the sensitivity analysis: (Line 428) “Parameters included in the sensitivity analysis were developed from the theoretical elements of LHNs, as described above and in Table 1. Other parameters in the model were developed to enable simulation of different health conditions (e.g., number of phenotypes, number of treatments, severity, course, relapse probability) or to simulate different initial conditions (e.g., patients joining the network over time vs already all enrolled, patients entering and leaving the cohort vs closed cohort). These parameters were held constant in the sensitivity analysis.”

Also, clear implementation steps for healthcare services/systems to implement the ABM would be useful.

- Thank you for this suggestion. We have added text to the Future Studies section regarding using the ABM to develop and implement actionable interventions (Line 811).

The authors could also explore additional outcomes such as cost effectiveness, equity, etc., so as to broaden the model's utility.

- We agree that these additional outcomes would broaden the model’s utility and have included this suggestion in limitations (Line 798).
